# Neoadjuvant immunotherapy with or without chemotherapy in locally advanced oral squamous cell carcinoma: Randomized, two-arm, phase 2 trial

**DOI:** 10.1016/j.xcrm.2025.101930

**Published:** 2025-01-30

**Authors:** Hai-Ming Liu, Xue-Peng Xiong, Zi-Li Yu, Zhe Shao, Gai-Li Chen, Yu-Tong Liu, Xin-Xin Wang, Qiu-Yun Fu, Xiao-Xia Cheng, Jing Li, Jia-Li Zhang, Bo Li, Hong-Yun Gong, Ya-Hua Zhong, Wei Zhang, Jun Jia, Bing Liu, Gang Chen

**Affiliations:** 1 State Key Laboratory of Oral & Maxillofacial Reconstruction and Regeneration, Key Laboratory of Oral Biomedicine Ministry of Education, Hubei Key Laboratory of Stomatology, School & Hospital of Stomatology, Wuhan University, Wuhan 430079, China; 2 Department of Oral and Maxillofacial Surgery, School and Hospital of Stomatology, Wuhan University, Wuhan 430079, China; 3 Department of Radiation and Medical Oncology, Hubei Key Laboratory of Tumor Biological Behaviors, Hubei Cancer Clinical Study Center, Zhongnan Hospital of Wuhan University, Wuhan 430071, China; 4 Department of Oral Pathology, School and Hospital of Stomatology, Wuhan University, Wuhan 430079, China; 5 Department of Oral Radiology, School and Hospital of Stomatology, Wuhan University, Wuhan 430079, China; 6 Cancer Center, Renmin Hospital of Wuhan University, Wuhan 430060, China; 7 TaiKang Center for Life and Medical Sciences, Wuhan University, Wuhan 430071, China; 8 Frontier Science Center for Immunology and Metabolism, Wuhan University, Wuhan 430071, China

**Keywords:** oral squamous cell carcinoma, neoadjuvant therapy, PD-1 inhibitor, chemotherapy, pathological response

## Abstract

Patients with locally advanced oral squamous cell carcinoma (OSCC) have poor outcomes with standard care. Neoadjuvant therapy is shown to be effective for these patients. In the randomized, two-arm, phase 2, non-comparative trial, we investigate the efficacy and safety of the neoadjuvant programmed cell death 1 (PD-1) inhibitor camrelizumab with or without docetaxel-cisplatin-5-fluorouracil (TPF) chemotherapy in patients with resectable locally advanced OSCC. Patients with stage III–IVA OSCC receive neoadjuvant therapy with three cycles of camrelizumab (arm Cam) with or without two cycles of TPF chemotherapy (arm Cam+TPF), followed by surgery and adjuvant therapy. Major pathological response (MPR) is achieved in both arm Cam (5/34, 14.7%) and arm Cam+TPF (26/34, 76.4%). With a median follow-up of 32 months, the 2-year event-free survival (EFS) rate of arm Cam and Cam+TPF is 52.9% and 91.2%, respectively. This work demonstrates feasibility and safety for immunochemotherapy in the neoadjuvant setting for OSCC. This study was registered at ClinicalTrials.gov (NCT04649476).

## Highlights

• Neoadjuvant camrelizumab plus TPF chemotherapy are feasible in locally advanced OSCC
• The combination regimen is well tolerated and associated with durable survival benefits
• Pathological partial response is a surrogate primary endpoint for neoadjuvant therapy
• Non-responders to neoadjuvant immunochemotherapy necessitate close monitoring

## Graphical abstract

**Figure:**
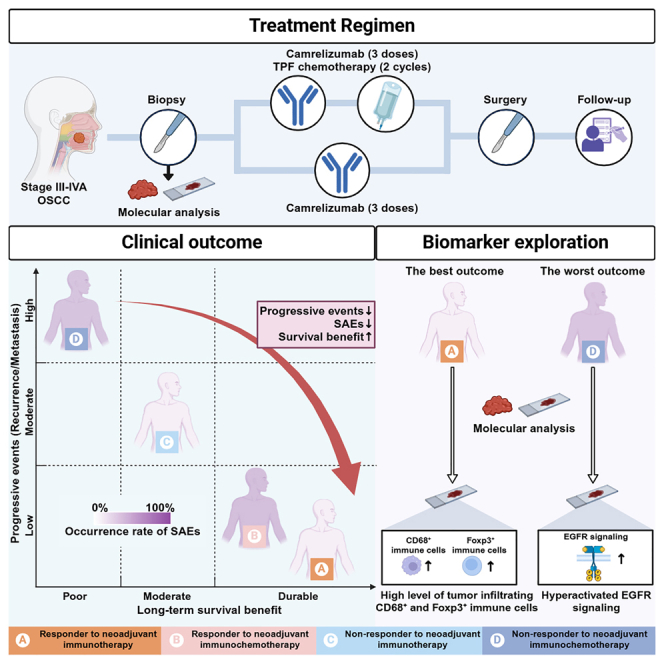

## Teaser

Patients with locally advanced oral squamous cell carcinoma often experience poor outcomes with standard care. Liu et al. conduct a randomized, two-arm, phase 2, non-comparative trial to evaluate the feasibility and safety profile of neoadjuvant camrelizumab, either alone or in combination with TPF chemotherapy, in patients with resectable locally advanced OSCC.

## Introduction

Oral squamous cell carcinoma (OSCC), a prevalent subset of head and neck squamous cell carcinoma (HNSCC), is diagnosed with locally advanced disease in more than 60% of patients.^1^ Surgical resection followed by adjuvant radiotherapy with or without chemotherapy has been recommended as the standard of care for patients with locally advanced OSCC. However, the prognosis of this subset of patients remains poor, with a high risk of recurrence or metastasis. Their 5-year overall survival (OS) rate is still less than 50%.^2^^,^^3^^,^^4^^,^^5^ A novel therapeutic modality is needed for these patients.

Neoadjuvant therapy, designed to reduce the burden of locoregional disease before surgery, has emerged as a promising treatment strategy. Research indicates that neoadjuvant chemotherapy can elicit pathological responses in patients with locally advanced OSCC. Specifically, major pathological response (MPR) rates have been reported at 27.7% for neoadjuvant docetaxel-cisplatin-5-fluorouracil (TPF) and 33% for cisplatin-5-fluorouracil (PF) chemotherapy. Impressive clinical regression rates of 80.6% and 82% have been observed for TPF and PF chemotherapy, respectively, in patients with locally advanced OSCC.^6^^,^^7^ However, it is noteworthy that no significant difference in survival advantage has been identified between patients who received neoadjuvant chemotherapy and those who did not, indicating that current neoadjuvant chemotherapy regimens may not enhance long-term survival outcomes.

Recent advancements in immune checkpoint inhibitors have shown promising results in the management of recurrent and metastatic HNSCC, including OSCC.^8^ The successes have prompted investigations into their potential use in the neoadjuvant setting. Clinical trials have demonstrated the efficacy and safety of neoadjuvant mono-immunotherapy in HNSCC, using agents such as pembrolizumab^9^^,^^10^ and nivolumab.^11^^,^^12^^,^^13^ Reported major pathological response rates for pembrolizumab range from 5% to 7%, while those for nivolumab range from 5.9% to 17%. Despite these relatively low MPR rates, long-term outcomes appear favorable. For instance, Uppaluri et al. demonstrated that neoadjuvant pembrolizumab therapy decreased the 1-year relapse rate among patients with locally advanced human papilloma virus (HPV)-unrelated HNSCC with high-risk pathology (positive margins and/or extranodal extension).^9^ Additionally, Wise-Draper et al. found that patients who achieved a pathological response to neoadjuvant pembrolizumab therapy had significantly improved disease-free survival (DFS) rates in the first year (93% vs. 72%).^10^ Knochelmann et al. reported a 2-year OS rate of 83% among 12 patients with resectable stage II-IVA OSCC.^11^ Ferris et al. reported 2-year recurrence-free survival rates of 88.2% for patients with HPV-positive HNSCC (stage III–IV),^12^ while Schoenfeld et al. reported 1-year progression-free survival and OS rates of 85% and 89% for patients with stage II–IVA OSCC, respectively.^13^ Subsequently, studies have been dedicated to enhancing the MPR rate by combining neoadjuvant immunotherapy with other treatment regimens including durvalumab plus tremelimumab,^14^ nivolumab plus ipilimumab,^13^^,^^15^ and nivolumab plus lirilumab.^16^ A recent study reported that the combination of anti-programmed cell death 1 (PD-1) and anti-angiogenesis therapy in locally advanced OSCC improved the MPR rate to 40%.^17^ This highlights the need to explore additional approaches to enhance the MPR rate of neoadjuvant immunotherapy.

In addition to the direct cytotoxic effects on cancer cells, emerging evidence indicated that chemotherapy may also modulate the immune system, including weakening the activity of T regulatory cells and enhancing the presentation of tumor antigens, suggesting a possibility for the combination of neoadjuvant immunotherapy and chemotherapy. Yang et al. recently reported an MPR rate of 76.0% in 27 patients with locally advanced HNSCC (93.3% oropharyngeal squamous cell carcinoma [OPSCC] and 6.7% OSCC).^18^ In single-arm phase 1 and phase 2 trial, the MPR rate was reported as 60% and 63% when the combination of anti-PD-1 (toripalimab or camrelizumab) and chemotherapy (albumin paclitaxel and cisplatin) was administered in patients with locally advanced HNSCC.^19^^,^^20^ However, another single-arm phase 1b clinical trial has reported that the combination of anti-PD-1 (toripalimab) and chemotherapy (gemcitabine and cisplatin) achieved an MPR rate of only 44.5% in 18 patients with locally advanced HNSCC (30.3% OPSCC and 69.7% OSCC).^21^ These findings highlight variability in the efficacy of different neoadjuvant immunotherapy and chemotherapy combinations and suggest that response may differ between OSCC and OPSCC. Therefore, further evidence is needed to substantiate the efficacy of neoadjuvant immunochemotherapy in locally advanced OSCC.

Camrelizumab is a fully humanized anti-PD-1 monoclonal antibody that binds to a different site on PD-1 compared to nivolumab and pembrolizumab.^22^^,^^23^^,^^24^ Its efficacy as a monotherapy in HNSCC remains unexplored. In this study, we report a randomized, two-arm, phase 2, non-comparative trial of neoadjuvant PD-1 inhibitor camrelizumab alone (arm Cam) or combined with TPF chemotherapy (arm Cam+TPF), followed by surgery and adjuvant therapy in 68 patients with stage III–IVA OSCC. The primary objectives are to assess the efficacy, survival benefit, and safety profiles of neoadjuvant immunochemotherapy and immunotherapy in patients with locally advanced OSCC.

## Results

### Patient characteristics

**Figure 1:**
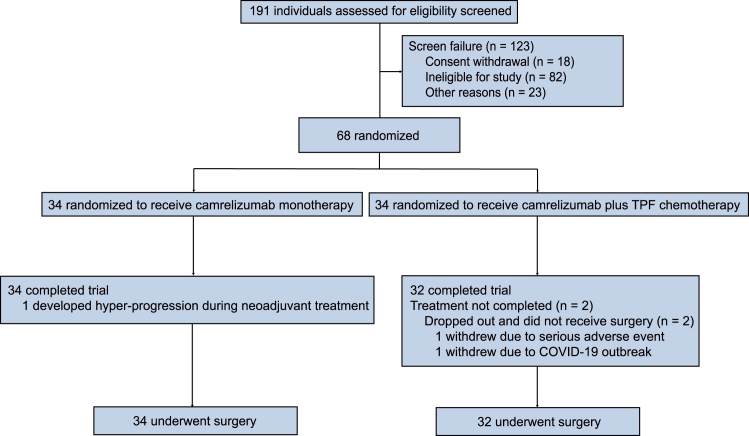
Trial flow diagram A total of 68 patients were enrolled in this trial, with 34 patients completing the full treatment protocol in arm Cam, and 32 patients completing the full treatment protocol in arm Cam+TPF.

From March 2021 to July 2022, 191 patients were assessed for eligibility, and 68 evaluable patients were enrolled (Figure 1; Figure S1). Thirty-four patients were randomized to arm Cam and 34 to arm Cam+TPF. The baseline characteristics of the 68 enrolled patients are summarized in Table 1. The average age was 50.7 years (range, 32–68 years), and the majority of patients were male (59, 87%), current or former smokers (46, 68%), and had Eastern Cooperative Oncology Group performance status of 0 (44, 65%). The most common primary tumor site was tongue (36, 53%). Thirty-two patients (47%) had clinically or radiographically node-positive disease, and 29 patients (43%) had stage IVA disease (pre-therapy). Programmed cell death-ligand 1 (PD-L1) combined positive score (CPS) at baseline was 1%–19% in 30 patients (44%) and 20% or higher in 9 patients (13%).

**Table 1:** Baseline characteristics of the 68 enrolled patients

Characteristic	Overall (*n* = 68)	Arm Cam Camrelizumab (*n* = 34)	Arm Cam + TPF Camrelizumab + TPF (*n* = 34)
Age, mean (range)	50.7 (32.0–68.0)	52.0 (32.0–67.0)	49.5 (33.0–68.0)

**Gender, *n* (%)**			

Male	59 (86.8)	28 (82.4)	31 (91.2)
Female	9 (13.2)	6 (17.6)	3 (8.8)

**Smoking history, *n* (%)**			

Current or former	46 (67.6)	24 (70.6)	22 (64.7)
Never	22 (32.4)	10 (29.4)	12 (35.3)

**Alcohol use history, *n* (%)**			

Current or former	33 (48.5)	15 (44.1)	18 (52.9)
Never	35 (51.5)	19 (55.9)	16 (47.1)

**ECOG performance status, *n* (%)**			

0	44 (64.7)	18 (52.9)	26 (76.5)
1	24 (35.3)	16 (47.1)	8 (23.5)

**Tumor site, *n* (%)**			

Oral tongue	36 (52.9)	18 (52.9)	18 (52.9)
Gingiva	8 (11.8)	5 (14.7)	3 (8.8)
Floor of mouth	8 (11.8)	4 (11.8)	4 (11.8)
Buccal mucosa	16 (23.5)	7 (20.6)	9 (26.5)

**Clinical T-stage**^a^**, *n* (%)**			

T2	15 (22.1)	6 (17.6)	9 (26.5)
T3	37 (54.4)	19 (55.9)	18 (52.9)
T4	16 (23.5)	9 (26.5)	7 (20.6)

**Clinical N-stage**^a^**, *n* (%)**			

N0	36 (53.0)	23 (67.6)	13 (38.2)
N1	16 (23.5)	6 (17.7)	10 (29.4)
N2	16 (23.5)	5 (14.7)	11 (32.4)

**Clinical disease stage, *n* (%)**			

III	39 (57.4)	21 (61.8)	18 (52.9)
IVA	29 (42.6)	13 (38.2)	16 (47.1)

**PD-L1 combined positive score, *n* (%)**			

<1	29 (42.6)	16 (47.1)	13 (38.2)
1–19	30 (44.2)	12 (35.3)	18 (53.0)
≥20	9 (13.2)	6 (17.6)	3 (8.8)

Abbreviation: ECOG, Eastern Cooperative Oncology Group

a American Joint Committee on Cancer, 8^th^ Edition staging.

### Treatment characteristics

Among the 68 enrolled patients, 33 patients completed full treatment protocol in arm Cam, and 32 patients completed full treatment protocol in arm Cam+TPF. One patient in arm Cam received two cycles of neoadjuvant immunotherapy but experienced disease hyperprogression. Although this patient underwent surgical treatment, he developed distant metastasis and passed away 5 months after surgery. In arm Cam+TPF, two patients did not complete the full treatment protocol. One received one cycle of camrelizumab plus TPF chemotherapy and dropped out due to serious adverse event. The other patient received one cycle of camrelizumab but declined further treatment due to the COVID-19 outbreak, subsequently undergoing surgery and receiving postoperative radiotherapy. This patient remains alive with no signs of tumor recurrence or metastasis. Following neoadjuvant therapy, 66 patients underwent radical resection of the primary tumor along with neck lymph node dissection. The surgical procedures for patients in both arm Cam and arm Cam+TPF are detailed in Tables S1 and S2. Notably, the types of surgical procedures for the Cam+TPF group were not altered according to therapeutic effects. The proportions of patients undergoing glossectomy, mandibular resection, neck dissection, and flap reconstruction were generally consistent between the two arms. Three patients in arm Cam+TPF, including two with cancer involving the bilateral floor of the mouth and one with cancer involving the left mandibular gingiva, underwent extensive segmental mandibulectomy and received fibular myocutaneous flap reconstruction.

60 patients received postoperative risk-adaptive adjuvant therapy within 4–6 weeks after surgery. Among them, 44 patients (20 in arm Cam and 24 in arm Cam+TPF) received adjuvant radiotherapy. Additionally, 11 patients in arm Cam (5 with extracapsular extension, 3 with poorly differentiated pathology, and 3 with facial invasion) and 5 patients in arm Cam+TPF (3 with extracapsular extension and 2 with facial invasion) received concurrent radiotherapy with platinum-based chemotherapy. Three patients in arm Cam+TPF who had extracapsular extension rejected to receive postoperative adjuvant therapy (see Tables S3 and S4). Although patients with high-risk pathological factors received intensified postoperative adjuvant therapy, a relatively high incidence of treatment failure (recurrence/metastasis/death) was still observed.

### Efficacy outcomes

**Figure 2:**
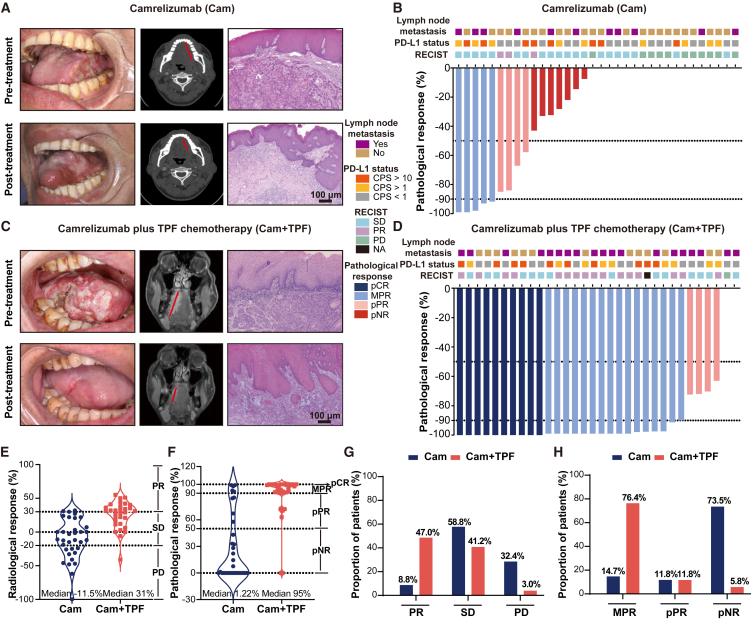
Patients’ tumor response in the subgroups (A) Major pathological response in a 53-year-old man (T3N2bM0) who received neoadjuvant camrelizumab mono-immunotherapy. Images of the left tongue (left), radiological images (middle), and pathological images (right) before (top) and after (bottom) neoadjuvant therapy were shown; scale bar, 100 μm; red line indicates the longest diameter of the tumor. (B) Waterfall plot showing patients’ pathological response and radiological response in arm Cam. (C) Major pathological response in a 56-year-old man (T3N0M0) who received neoadjuvant camrelizumab immunotherapy plus TPF chemotherapy. Images of the right tongue (left), radiological images (middle), and pathological images (right) before (top) and after (bottom) neoadjuvant therapy were shown; scale bar, 100 μm; red line indicates the longest diameter of the tumor. (D) Waterfall plot showing patients’ pathological response and radiological response in arm Cam+TPF. (E) Percentage of radiological response rate in resected tumor specimens. Median percentage: arm Cam −11.5% (range −100% to 32%) and arm Cam+TPF 31% (range −41% to 55%). (F) Percentage of pathological response rate in resected tumor specimens. Median percentage: arm Cam 1.22% (range 0%–99%) and arm Cam+TPF 95% (range 0%–100%). (G) Proportion of patients with radiological response in the subgroups. (H) Proportion of patients with pathological response in the subgroups. CPS, combined positive score; PR, partial response; SD, stable disease; PD, progressive disease; NA, not applicable; pCR, pathological complete response; MPR, major pathological response; pPR, pathological partial response; pNR, pathological non-response.

Figures 2A and 2C depict representative examples of two patients with T3N2bM0 and T3N0M0 OSCC involving the tongue in arm Cam (top) and arm Cam+TPF (bottom), respectively. These images demonstrate striking responses to the respective treatment regimens. The waterfall plots of radiological and pathological response for individual patients in arm Cam and arm Cam+TPF are shown in Figures 2B and 2D. As shown in Table S5, among the intention-to-treat population, 5 patients (5/34, 14.7%, 95% confidence interval [CI] 6.4%–30.1%) achieved MPR, and no patients achieved pathological complete response (pCR) in arm Cam. In arm Cam+TPF, the MPR was observed in 26 out of 34 patients (76.4%, 95% CI 60.0%–87.5%), including 10 patients (29.4%) who achieved pCR. The R0 resection rate was 100% in both arm Cam and arm Cam+TPF.

Regarding radiological response, we evaluated both primary tumor and lymph node metastasis. In arm Cam, 3 patients (8.8%) achieved a partial response (PR) according to the Response Evaluation Criteria in Solid Tumors (RECIST), while in arm Cam+TPF, 16 patients (47.0%) achieved PR. The overall response rates of these metastatic lymph nodes (LNs) in arm Cam and Cam+TPF were both 2.9%. Notably, the clinical response rates for these LNs were poorer compared to the primary tumor. A correlation between pathological and radiological response rates was observed (Figure S2A). Specifically, all patients with progressive disease (PD as per RECIST) corresponded to pathological non-response (pNR), and all patients achieved PR (PR as per RECIST) corresponded to pathological PR (pPR), MPR, and pCR. However, patients who achieved stable disease (SD as per RECIST) corresponded to all types of pathological response including pNR, pPR, MPR, and pCR, indicating a wide range of pathological responses in patients with SD on radiological imaging (Figure S2B).

These data indicate that neoadjuvant camrelizumab combined with TPF chemotherapy exhibited a superior radiological and pathological response rate (Figures 2E and 2F; Table S5). Subgroup analysis revealed that majority of patients receiving this combination therapy achieved PR and MPR (Figures 2G and 2H). As shown in Table S6, most patients in arm Cam did not experience significant changes in tumor staging, while 27 patients (79%) in the arm Cam+TPF experienced clinical to pathological downstaging post neoadjuvant therapy. It was observed that patients with positive PD-L1 expression (PD-L1 CPS > 1) had higher response rates in both two arms or in arm Cam, but the difference was not significant (Figures S3A and S3B).

### Safety

Treatment-related adverse events (TRAEs) observed in the study are summarized in Table 2. In arm Cam, the most common TRAEs of any grade were reactive cutaneous capillary endothelial proliferation (RCCEP, 29, 85%), followed by anemia (7, 21%). In arm Cam+TPF, the most common TRAEs were alopecia (26, 76%), followed by anemia (23, 68%) and RCCEP (22, 65%). Two patients (6%) in arm Cam experienced grade 3–4 TRAEs. Eight patients (23%) in arm Cam+TPF experienced grade 3–4 TRAEs, with six of these patients having more than one grade 3–4 TRAEs. The grade 3–4 TRAEs observed in arm Cam+TPF were neutropenia (7, 21%), leukopenia (5, 15%), lymphopenia (3, 9%), and diarrhea (1, 3%). One patient in arm Cam+TPF experienced multiple episodes of vomiting during neoadjuvant treatment, and this cascade of events ultimately led to cardiac insufficiency, followed by anuria. Unfortunately, the patient subsequently passed away due to multiple organ dysfunction. Surgery-related adverse events were reported for 6 of 34 patients (17.6%) in arm Cam and 5 of 32 patients (15.6%) in arm Cam+TPF, most of which were grade 1 or 2 (Table S7). The most frequent surgery-related adverse events were fever (9%) in arm Cam and flap swelling (9%) in arm Cam+TPF. Two patients (1 in arm Cam and 1 in arm Cam+TPF) experienced flap vascular crisis after surgery. These results collectively indicated that both neoadjuvant camrelizumab alone and neoadjuvant camrelizumab plus TPF chemotherapy were considered feasible in patients with locally advanced OSCC.

**Table 2:** Treatment-related adverse events (common terminology criteria for adverse events version 5.0) in 68 enrolled participants

	Arm Cam (*n* = 34) *n* (%)			Arm Cam + TPF (*n* = 34) *n* (%)		
	Grade 1 or 2	Grade 3	Grade 4	Grade 1 or 2	Grade 3	Grade 4
RCCEP	29 (85)	0	0	22 (65)	0	0
Anemia	7 (21)	0	0	23 (68)	0	0
Mucositis oral	6 (18)	0	0	8 (24)	0	0
Creatinine increased	5 (15)	0	0	17 (50)	0	0
Lymphopenia	4 (12)	0	0	16 (47)	3 (9)	0
GGT increased	4 (12)	0	0	6 (18)	0	0
ALT increased	3 (9)	1 (3)	0	12 (35)	0	0
Lactate dehydrogenase increased	2 (6)	0	0	10 (29)	0	0
AST increased	2 (6)	0	0	8 (24)	0	0
Neutrophilia	2 (6)	0	0	0	0	0
Diarrhea	1 (3)	0	0	8 (24)	0	1 (3)
Leukocytosis	1 (3)	0	0	0	0	0
Creatine kinase increased	0	1 (3)	0	10 (29)	0	0
Leukopenia	0	0	0	9 (26)	4 (12)	1 (3)
Neutropenia	0	0	0	7 (21)	4 (12)	3 (9)
Thrombocytopenia	0	0	0	3 (9)	0	0
Monocyte decreased	0	0	0	2 (6)	0	0
Monocyte increased	0	0	0	1 (3)	0	0
Red blood cell decreased	0	0	0	2 (6)	0	0
Alopecia	0	0	0	26 (76)	0	0
Vomiting	0	0	0	10 (29)	0	0
Creatine kinase isoenzyme increased	0	0	0	1 (3)	0	0
Diminution of vision	0	0	0	1 (3)	0	0

Abbreviation: RCCEP, reactive cutaneous capillary endothelial proliferation; GGT,gamma-glutamyl transpeptidase; ALT, alanine aminotransferase; AST, aspartate aminotransferase.

### Survival outcomes

**Figure 3:**
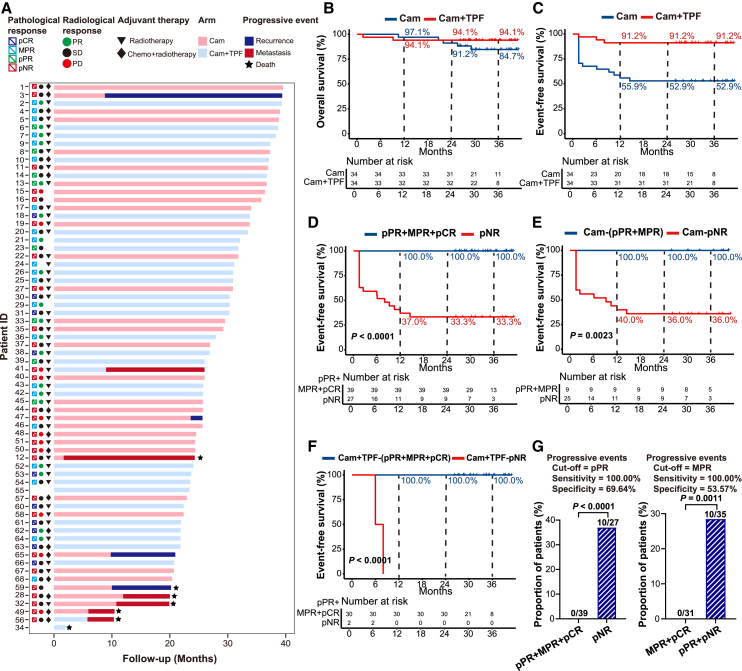
Follow-up and survival outcomes of patients (A) Swimmer plot showing patients’ survival outcomes. (B) Kaplan-Meier curve of overall survival for the patients in arm Cam and arm Cam+TPF. (C) Kaplan-Meier curve of event-free survival for the patients in arm Cam and arm Cam+TPF. (D) Kaplan-Meier curve of event-free survival for the pathological responders (pPR+MPR+pCR) and non-responders (pNR). Log rank *p* value was used to estimate the significance among groups. (E) Kaplan-Meier curve of event-free survival for the pathological responders (pPR+MPR) and non-responders (pNR) in arm Cam. Log rank *p* value was used to estimate the significance among groups. (F) Kaplan-Meier curve of event-free survival for the pathological responders (pPR+MPR+pCR) and non-responders (pNR) in arm Cam+TPF. Log rank *p* value was used to estimate the significance among groups. (G) Proportion of patients who had progressive events in different subgroups. *p* value was determined by two-sided Fisher’s exact test. PR, partial response; SD, stable disease; PD, progressive disease; pCR, pathological complete response; MPR, major pathological response; pPR, pathological partial response; pNR, pathological non-response.

At the time of data analysis, the median follow-up time was 32 months. From 2.5 months to 28.1 months after treatment initiation in arm Cam, 4 patients (4/34, 11.7%) experienced locoregional recurrences, and 1 patient succumbed to the disease. Four patients (4/34, 11.7%) experienced distant metastasis and succumbed to the disease. In arm Cam+TPF, 2 patients (2/34, 5.8%) experienced distant metastasis within 6–8 months after treatment initiation, and one of them later died (Figure 3A). Another patient in arm Cam+TPF died because of multiple organ dysfunction before surgery. The 2-year OS rate in arm Cam and Cam+TPF was 91.2% (95% CI 82.1%–100.0%) and 94.1% (95% CI 86.5%–100.0%) (Figure 3B), respectively. The 2-year DFS rate and 2-year event-free survival (EFS) rate in arm Cam+TPF were 93.9% (95% CI 86.1%–100.0%) and 91.2% (95% CI 82.1%–100.0%) (Figures S4A and 3C). These data demonstrate that the incorporation of TPF chemotherapy with camrelizumab in arm Cam+TPF led to durable survival benefits in patients with locally advanced OSCC.

It was noticed that all the patients experiencing progressive events were pathological non-responders (Figure 3A). Consistently, the comparative analysis of outcomes among patients achieving different pathological responses revealed that those who pathologically responded to neoadjuvant immunotherapy or immunochemotherapy (pPR or MPR, pCR) had significantly higher EFS rates compared to non-responders (pNR) (Figure S4B). It was further observed that if pPR was set as the cutoff value, patients who achieved a pathological response (pPR or MPR, pCR) had a significant improvement in EFS (Figure 3D). By virtue of the two-arm design, we found that patients with pPR, either in arm Cam (Figures 3E and S4C) or arm Cam+TPF (Figures 3F and S4D), had similar EFS to patients achieving MPR in the first 3 years. These findings suggest that a pathological response (pCR, MPR, and pPR) to neoadjuvant therapy leads to a significant improvement in EFS. Notably, further analysis revealed that using pPR as the optimal cutoff value resulted in the same predictive performance in event probability between the two divided populations compared with MPR (Figure 3G). According to RECIST criteria, patients who suffered PD all had a terrible prognosis, while patients who achieved PR had a favorable prognosis (Figure S5A). As aforementioned, patients who achieved SD may have distinct pathological responses, so it was not sufficient enough to confer long-term survival benefit (Figure S5B). According to PD-L1 expression, patients seemed to benefit from positive PD-L1 expression in term of EFS, but the difference was not significant (Figures S6A and S6B). Taken together, these results demonstrate that pathological responders tend to have better EFS compared to non-responders. Additionally, achieving a pPR to neoadjuvant immunotherapy or immunochemotherapy may serve as a surrogate primary study endpoint, as it shows the same predictive value as MPR in patients with locally advanced OSCC.

### High metastatic rate and poor prognosis of non-responders to neoadjuvant immunochemotherapy

Notably, there were still a few individuals who exhibited resistance to neoadjuvant immunochemotherapy. Even worse, non-responders in arm Cam+TPF suffered an extremely higher risk of postoperative metastasis (2/2, 100%). Moreover, non-responders in arm Cam+TPF suffered even worse prognosis compared with patients who received standard of care in terms of DFS (2-year DFS 0% vs. 63%–73%^15^^,^^25^) and OS (2-year OS 50% vs. 60%–68%^6^^,^^25^^,^^26^). Therefore, our study emphasizes the clinical importance of close monitoring and early intervention of the non-responders who underwent neoadjuvant immunochemotherapy in case of progressive events.

**Figure 4:**
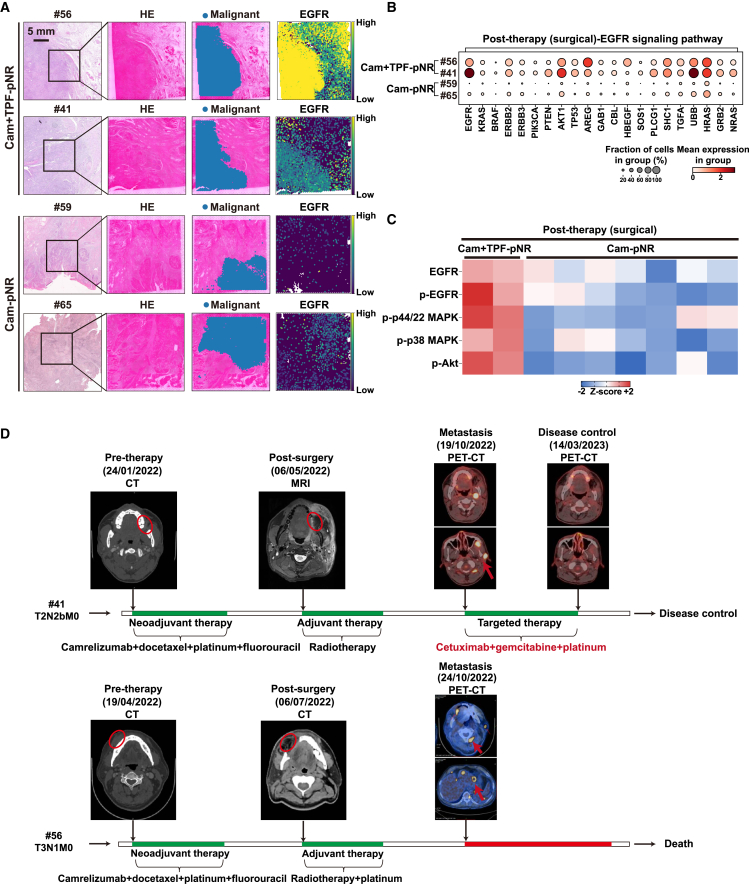
Worse prognosis of non-responders in arm Cam+TPF (A) Spatial heatmaps showing expression intensity of *EGFR*, including Cam+TPF-pNR group (*n* = 2) and Cam-pNR group (*n* = 2); scale bar, 5 mm. (B) Dot plot showing expression levels of genes related to EGFR signaling pathway, including Cam+TPF-pNR group (*n* = 2) and Cam-pNR group (*n* = 2). (C) Heatmap showing the quantitative immunohistochemical results of post-therapy EGFR and downstream proteins in non-responders in arm Cam+TPF compared with arm Cam, including Cam+TPF-pNR group (*n* = 2) and Cam-pNR group (*n* = 7). (D) Follow-up of the two non-responders in arm Cam+TPF. Red circle indicates primary tumor, red arrow indicates tumor metastasis. EGFR, epidermal growth factor receptor; CT, computed tomography; MRI, magnetic resonance imaging; PET-CT, positron emission tomography and computed tomography.

To investigate the underlying mechanisms and identify potential signatures of non-responders to neoadjuvant immunochemotherapy, we performed an analysis using spatial transcriptomics. The results revealed elevated epidermal growth factor receptor (EGFR) signaling pathway at the tumor borders, primarily in tumor cells, in the two non-responders (#41 and #56) in arm Cam+TPF compared to the non-responders (#59 and #65) in arm Cam (Figures 4A and 4B). Consistently, strong staining of EGFR, phosphorylated EGFR (p-), and downstream molecules were observed in both the biopsy and surgical specimens (Figures S7 and S8). Further quantitative analysis showed that the aforementioned molecules were obviously overexpressed in non-responders in arm Cam+TPF compared with arm Cam before and after the therapy (Figures 4C and S9).

However, the two non-responders in arm Cam+TPF experienced distinctive prognosis due to the difference in following treatment. After being diagnosed with metastasis after surgery, patient #41 received the therapy of cetuximab plus gemcitabine and platinum. Strikingly, the size of metastatic lesion rapidly reduced, and the disease was effectively controlled. However, patient #56 refused to receive salvage therapy and unfortunately passed away (red circle indicates primary lesion and red arrow indicates metastatic lesion) (Figure 4D). These aforementioned results suggest that EGFR hyperactivation may play an important role in resistance to neoadjuvant immunochemotherapy.

### Decision-making strategy

To facilitate pre-treatment screening of patients toward precision immunotherapy in the future, the patients in our clinical trial were classified into four groups based on their long-term survival, progressive events (recurrence/metastasis), and severe adverse events (grade 3–5 adverse events, SAEs) (Figure S10): A, responders to neoadjuvant immunotherapy; B, responders to neoadjuvant immunochemotherapy; C, non-responders to neoadjuvant immunotherapy; and D, non-responders to neoadjuvant immunochemotherapy. Among the four groups of patients, we think group A is the most preferred, followed by group B, while group D is the most undesired. An ideal screening process is that we first select patients who might respond to neoadjuvant mono-immunotherapy (group A), and then the remaining patients all turn into neoadjuvant immunochemotherapy, but before that, we should exclude the potential non-responders to neoadjuvant immunochemotherapy (group D). For this purpose, we conducted exploratory subgroup analyses to find the potential signatures of patients in group A according to baseline clinical characteristics and a panel of checkpoint molecules and immunocytes examined in pre-therapy biopsy tissues. There was no significant difference between groups based on clinical characteristics (Table S8). Patients with a high infiltration level of CD68^+^ cells and Foxp3^+^ cells in tumor tissues are likely to benefit from neoadjuvant mono-immunotherapy (Table S9). As aforementioned, patients with hyperactivated EGFR are potential non-responders to neoadjuvant immunochemotherapy.

## Discussion

In this study, we reported a randomized, two-arm clinical trial exploring the efficacy and safety of neoadjuvant immunochemotherapy and neoadjuvant immunotherapy in patients with locally advanced OSCC. Consistent with previous studies, neoadjuvant immunotherapy was effective in only a small portion of patients, and some individuals experienced disease progression during the neoadjuvant immunotherapy period.^11^ In contrast, neoadjuvant immunochemotherapy yielded an extremely higher MPR rate (76.4%) and led to durable survival benefit (2-year OS 94.1%, 2-year EFS 91.2%) with acceptable safety profiles. Additionally, compared with the patients who received standard of care, neoadjuvant immunochemotherapy led to improved OS (2-year OS 94.1% vs. 60%–68%^6^^,^^25^^,^^26^), indicating the potential value for further clinical expansion.

Prior studies have indicated that combining neoadjuvant immunotherapy with chemotherapy can enhance response rates in HNSCC. For instance, Zhang et al. reported an MPR rate of 74.1% and a pCR rate of 37% among patients with HNSCC, with 93.3% of cases classified as OPSCC and 6.7% as OSCC.^18^ Similarly, Wu et al. found an MPR rate of 63% and a pCR rate of 55.6% in HNSCC, with 66.7% of cases as OPSCC and 33.3% as OSCC.^20^ The higher pCR rates observed in these studies may be attributed to the higher proportion of OPSCC cases, which are generally more sensitive to chemotherapy.

Our trial revealed an inconsistent objective response rate (ORR) between the primary sites and metastatic LNs. The ORR at primary sites was as high as 47%, whereas it was significantly lower at 2.9% for metastatic LNs. This pattern aligns with findings from other studies, which reported MPR rates of up to 60% in primary tumors compared to only 17% in lymph nodes.^19^ Additionally, a separate study on neoadjuvant anti-PD-1 therapy in HNSCC indicated better pathological responses in LNs than at primary sites.^27^ The current literature lacks a systematic evaluation of the regression rates in primary tumors versus metastatic LNs in neoadjuvant therapy for HNSCC. Future research should prioritize the assessment of metastatic LNs in the context of neoadjuvant therapies to better understand these discrepancies and optimize treatment strategies.

Previously, the IMCISION study indicated that only patients who achieved MPR to neoadjuvant nivolumab plus ipilimumab experienced improved outcomes in terms of EFS and OS based on limited enrolled patients.^15^ In our study, by virtue of the two-arm design, we found that patients with pPR, either in the arm of neoadjuvant immunotherapy or neoadjuvant immunochemotherapy, had a similar 2-year EFS compared with MPR (100% vs. 100%), whereas those with pNR had poor EFS (33.3%). These results suggested that, different from previously reported MPR, pathological response (pCR, MPR, and pPR) to neoadjuvant immunotherapy or neoadjuvant immunochemotherapy was sufficient to confer excellent EFS in patients with OSCC. A recent single-arm study conducted by Wise-Draper revealed that patients with pathological response (tumor regression rate >20%) to neoadjuvant pembrolizumab had a significantly higher DFS in the first year compared to patients who did not respond (93% vs. 72%).^10^ But there were still a few patients who developed progressive events after surgery using the aforementioned criteria. Compared with the single-arm study, we identified that pPR (tumor regression rate > 50%) was more sufficient to confer excellent EFS in either neoadjuvant immunotherapy or immunochemotherapy.

Similar to other studies, it is easily understood that therapy resistance could occur in both the neoadjuvant immunotherapy group and the neoadjuvant immunochemotherapy group.^9^^,^^10^^,^^19^ However, our study revealed that non-responders to neoadjuvant immunochemotherapy suffered an extremely higher risk of metastasis and worse outcomes compared to patients who received standard of care. These results emphasize the clinical importance of close monitoring and early intervention of the non-responders who underwent neoadjuvant immunochemotherapy. Due to the limited sample size, the finding should be examined further in other cohorts. This also reminds us to screen the patients who will respond or resist to the therapy at an early stage. According to our signature analysis, the results suggest that patients with a high infiltration level of CD68^+^ cells and Foxp3^+^ cells in tumor tissues are likely to benefit from neoadjuvant mono-immunotherapy, and patients with hyperactivated EGFR signaling are potential non-responders to neoadjuvant immunochemotherapy.

In the neoadjuvant camrelizumab plus TPF chemotherapy arm, the most common grade 3–4 adverse events were leukopenia and neutropenia. These are known side effects of TPF chemotherapy^6^^,^^7^^,^^28^ and can be effectively managed through the administration of recombinant human granulocyte stimulating factor. The higher incidence rate of grade 3–4 adverse events did not increase the probability of surgical complications or cause any delays in surgery. Despite a relatively higher incidence rate of SAEs observed in arm neoadjuvant immunochemotherapy, our results still provide evidence that this treatment is considered feasible in patients with locally advanced OSCC. Of note, RCCEP was the most common side effect observed in either two arms. This is thought to be related to the potential pro-angiogenic function of the drug.^29^^,^^30^

In summary, neoadjuvant camrelizumab plus TPF chemotherapy led to durable survival benefit with a high pathological response rate and acceptable safety profile in locally advanced OSCC, and pPR appeared as a surrogate primary endpoint. However, non-responders to neoadjuvant immunochemotherapy seemed to have a terrible prognosis and require close monitoring and early intervention, which should be reported timely to benefit the future clinical trials and practices. Also, further evaluation of survival benefit of neoadjuvant immunochemotherapy in randomized, large-scale and multi-center clinical trials is warranted in the future.

### Limitations of the study

This trial has several limitations, including an imbalance in N stage between the groups, a small sample size, and a non-comparative two-arm design. First, we acknowledge the imbalance in N stage distribution, which may be attributed to the relatively small number of participants. Notably, the patient cohort in arm Cam+TPF had more advanced N stages, typically associated with poorer outcomes. Second, while our findings suggest that the combination of anti-PD-1 camrelizumab and TPF chemotherapy may offer superior efficacy, definitive conclusions should be drawn from randomized controlled studies, as this study lacks an appropriate control group. It is important to note that neoadjuvant therapy is not currently included in the standard treatment for resectable oral cancer. Considering the current study design and small sample size, we cannot draw definitive conclusions, especially regarding EFS and OS. Ultimately, definitive conclusions regarding neoadjuvant immunotherapy will dependent on well-designed and rigorously controlled randomized clinical trials.

## Resource availability

### Lead contact

Further information and requests for resources and reagents should be directed to and will be fulfilled by the lead contact, Gang Chen (geraldchan@whu.edu.cn).

### Materials availability

This study did not generate new unique reagents.

### Data and code availability

• The raw sequence data reported in this paper have been deposited in the Genome Sequence Archive (GSA) at the National Genomics Data Center, China National Center for Bioinformation/Beijing Institute of Genomics, and are publicly accessible at https://ngdc.cncb.ac.cn/gsa-human. Spatial transcriptomic data for patients #41 and #56 are available from GSA (HRA009397). Spatial transcriptomic data for patients #59 and #65 are available from GSA (HRA009391). All other relevant detailed clinical data could be available upon reasonable request from the lead contact.
• No custom computer codes are reported in this paper. The code for data analysis is available upon request.
• Any additional information required to reanalyze the data reported in this work paper is available from the lead contact upon request.

## Acknowledgments

This study was supported by 10.13039/501100001809National Natural Science Foundation of China (82341023 and 81922038), School and Hospital of Stomatology, Wuhan University (LYZX202001), the Innovative Research Team of High-level Local Universities in Shanghai (SHSMU-ZLCX20212300), the 10.13039/501100012226Fundamental Research Funds for the Central Universities (2042022dx0003 and 2042023kfyq02), and Jiangsu Hengrui Pharmaceuticals. We thank the patients and their families and all members of the collaborative group.

## Author contributions

G.C., B.Liu, and J.J. designed the study. G.C. was the principle investigator. H.-M.L., J.-L.Z., W.Z., and B.Li conducted the statistical design and analyses. X.-P.X., Z.-L.Y., Z.S., and J.J. recruited patients. X.-P.X., Z.S., J.J., B.Liu, G.-L.C., H.-Y.G., and Y.-H.Z. treated patients. H.-M.L., X.-P.X., Z.-L.Y., G.-L.C., Z.S., Y.-T.L., X.-X.W., Q.-Y.F., X.-X.C., W.Z., and J.L. collected and analyzed the data. Z.-L.Y., G.-L.C., H.-Y.G., W.Z., and Y.-H.Z. drafted the manuscript. Y.-H.Z., W.Z., and G.C. wrote the manuscript. All authors approved the manuscript.

## Declaration of interests

The authors declare no competing interests.

## STAR★Methods

### Key resources table

**Table:** 

REAGENT or RESOURCE	SOURCE	IDENTIFIER
**Antibodies**		

EGFR	Cell Signaling Technology	Cat #4267; RRID: AB_2246311
*p*-EGFR	Cell Signaling Technology	Cat #2234; RRID: AB_331701
*p*-p44/42 MAPK	Cell Signaling Technology	Cat #4370; RRID: AB_2315112
*p*-p38 MAPK	Cell Signaling Technology	Cat #4511; RRID: AB_2139682
*p*-Akt	Cell Signaling Technology	Cat #4060; RRID: AB_2315049
CD3ε	Cell Signaling Technology	Cat #85061; RRID: AB_2721019
CD4	Abcam	Cat #ab133616; RRID: AB_2750883
CD8α	Cell Signaling Technology	Cat #85336; RRID: AB_2800052
CD14	Abcam	Cat #ab182032; RRID: AB_3086692
CD68	Cell Signaling Technology	Cat #76437; RRID: AB_2799882
CD11b	Cell Signaling Technology	Cat #49420; RRID: AB_2799357
CD11c	Abcam	Cat #ab52632; RRID: AB_2129793
CD20	Abcam	Cat #ab64088; RRID: AB_1139386
Foxp3	Cell Signaling Technology	Cat #98377; RRID: AB_2747370
CD56	Cell Signaling Technology	Cat #3576; RRID: AB_2149540
TIM-3	Cell Signaling Technology	Cat #45208; RRID: AB_2716862
TIGIT	Abcam	Cat #ab243903; RRID: AB_2943164
PD-1	Cell Signaling Technology	Cat #43248; RRID: AB_2728836
PD-L1	Cell Signaling Technology	Cat #29122; RRID: AB_2798970

**Biological samples**		

Pre-treatment biopsy samples	This study	N/A
Post-treatment resected samples	This study	N/A

**Deposited data**		

Spatial transcriptomic data	This study	GSA: HRA009397, HRA009391

**Software and algorithms**		

GraphPad Prism (Version 8.0)	GraphPad Software, La Jolla California, USA	www.graphpad.com
R (Version 4.1.2)	R Development Core Team, 2008	www.r-project.org
Python (Version 3.9)	Python Software Foundation. Python Language Reference	www.python.org
Aperio digital pathology	Leica, Wetzlar, Germany	www.leicabiosystems.com

**Other**		

Nano Zoomer Digital Pathology scanner	Hamamatsu	Cat #C13239-01

### Experimental models and study participant details

#### Human subjects and ethical approval

Eligible patients, both male and female, were aged 18 to 70 years old and had not received prior treatment. They were newly diagnosed histologically proven oral squamous cell carcinoma, clinically staged as III-IVA (T1-2N1-2M0 or T3-4aN0-2M0 according to American Joint Committee on Cancer, AJCC 8th edition). All patients underwent chest CT and whole-body bone scintigraphy, ensuring a comprehensive assessment to exclude the presence of distant metastasis. All eligible patients had ECOG performance status of 0–1 with normal function of major organs and tolerance to the prescribed treatment regimen. The main exclusion criteria were as follows: patients who had experienced immune-related adverse events; those who had previously received antibody or drug targeting T cells or immune checkpoint pathways; and those requiring systemic steroid treatment due to autoimmune diseases. The detailed inclusion and exclusion criteria could be found in supplemented Method S1. After screening, sixty-eight eligible patients were randomly assigned (1:1) to receive camrelizumab (arm Cam) or camrelizumab plus TPF chemotherapy (arm Cam+TPF).

The study was done in accordance with the International Conference on Harmonization Guidelines on Good Clinical Practice and the Declaration of Helsinki. Written informed consent was provided by all study participants. Ethics approval was obtained from the Ethics Committee of the School and Hospital of Stomatology, Wuhan University ([2020] Ethics No.2).

#### Randomization and masking

Eligible patients were randomly assigned (1:1) to receive camrelizumab (arm Cam) or camrelizumab plus TPF chemotherapy (arm Cam+TPF). The randomization sequence was generated by an independent statistician using PASS 15.0 (NCSS LLC., Kaysville, U.T., USA). The treatment allocation was implemented via opaque, sealed envelopes. Patients, clinicians and study investigators were not masked to treatment assignments.

#### Sample size justification

The study was an exploratory study with two parallel groups with 1:1 randomization. The MPR rate of the camrelizumab group is expected to be 10%.^9^^,^^10^ We estimated a sample size of 27 cases would ensure that the precision of 95% confidence interval for the observed MPR is between 2.5% and 24.9%. Considering 20% dropout, the required sample size is 34 cases. According to two relevant single-arm clinical trials published in 2020 that investigated neoadjuvant immunotherapy combined with chemotherapy in non-small cell lung cancer (NSCLC),^31^^,^^32^ the reported MPR rates for squamous cell carcinoma in these studies were 68.7% and 80%. Given both non-small cell lung cancer (squamous cell carcinoma subtype) and oral squamous cell carcinoma fall under the same histological category, we hypothesized the MPR rate of the camrelizumab combined with TPF chemotherapy group is 70%. The precision of 95% confidence interval of the observed MPR is expected to be between 52.5% and 84.0% if 27 cases were enrolled in the group. Considering 20% dropout, the required sample size is 34 cases. Thus, the required total sample size is 68 cases.

### Method details

#### Study design

This investigator-initiated, single-center, open-label, two-arm, randomized, non-comparative phase II trial was done at the School and Hospital of Stomatology, Wuhan University (NCT04649476). The whole treatment schedule is shown in Figure S1. Enrolled patients were randomized to either arm Cam (neoadjuvant immunotherapy) or arm Cam+TPF (neoadjuvant immunotherapy combined with chemotherapy) to receive indicated treatment. In brief, all patients received 200 mg camrelizumab intravenously on days 1, 15 and 29. For TPF chemotherapy, patients received 75 mg/m^2^ docetaxel (T), 75 mg/m^2^ cisplatin (P) and 750 mg/m^2^ 5-fluorouracil (F) intravenously on days 1–5 and days 22–26. All patients received surgery 14 days after the last dose of camrelizumab. The former and final pre-operative imaging was performed 1–3 days before the randomization and surgery, which was used for radiological evaluation. The resection range of surgery was determined based on the pre-treatment tumor bed. Tattoo ink was applied to mark at least four points along the margins of the primary tumors, specifically on the longest and shortest axes. These markings were made 0.5 cm away from the palpable lesion margins. The resection range was determined 1.0 cm away from the markers. The deep margins were primarily determined based on CT imaging. By evaluating the anatomical locations reached by the lesion through CT imaging (for example, oral tongue cancer, whether it involves the lingual septum), the deep surgical margins were guided by the anatomical boundaries during the surgery. Intraoperative rapid frozen section biopsy of the tumor margins was performed to ensure complete resection of the tumor. Patients received postoperative risk-adaptive adjuvant therapy within 4–6 weeks after surgery. Patients with high-risk pathology features such as positive tumor margin (less than 5 mm or tumor cells observed on the margin), extracapsular lymph node metastasis, facial invasion (cheek) were supplemented with platinum-based chemotherapy concurrently with radiotherapy. All adverse events experienced by patients were collected from the first study intervention, throughout the whole study process, and at least 90 days after the last treatment.

#### Endpoints and clinical study assessment

The primary endpoint was MPR rate. Secondary endpoints were radiological response, 2-year event-free survival (EFS) rate, 2-year OS rate and treatment-related adverse events (TRAEs). The evaluation process of pathological response rate was shown in Figure S11. The entire tumor bed and all sampled lymph nodes were examined histologically in patients who had pathological complete response (pCR), which was defined as the absence of viable tumor in all slides. MPR was defined as the presence of 10% or less viable residual tumor in the resected tumor specimens. Pathological partial response (pPR) was defined as presence of more than 10% and less than 50% viable residual tumor and pathological non-response (pNR) was defined as presence of more than 50% viable residual tumor in the resected tumor specimens. The radiological response was evaluated by radiographic examinations and defined by Response Evaluation Criteria in Solid Tumors (RECIST) version 1.1. Adverse events were assessed according to the National Cancer Institute Common Terminology Criteria for Adverse Events (CTCAE) version 5.0 and Clavien-Dindo classification of surgical complications. Event-free survival was defined as the time from randomization to the first occurrence of disease progression or recurrence, or death from any cause. Disease-free survival was defined as the time from surgery to disease recurrence or death from any cause.

#### Tumor histology

To detect the expression of programmed cell death-ligand 1 (PD-L1), immunohistochemical staining was carried out using anti-PD-L1 (CST, 405.9A11, 29122S, 1:200). Combined positive score (CPS) was defined as the total number of PD-L1-stained cells (including tumor cells, tumor-associated lymphocytes, and macrophages) divided by the total number of viable tumor cells, multiplied by 100.

Tumor biopsy and surgical samples were collected and examined for a panel of checkpoint molecules, immunocyte subsets and epidermal growth factor receptor (EGFR) signaling proteins using immunohistochemistry, including EGFR (CST, D38B1, 4267S, 1:400), *p*-EGFR (Tyr1068) (CST, 2234S, 1:200), *p*-p44/42 MAPK (Erk1/2) (Thr202/Tyr204) (CST, D13.14.4E, 4370S, 1:200), *p*-p38 MAPK (Thr180/Tyr182) (CST, D3F9, 4511S, 1:200), *p*-Akt (Ser473) (CST, D9E, 4060S, 1:200), CD3ε (CST, D7A6E, 85061S, 1:200), CD4 (Abcam, ab133616, 1:400), CD8α (CST, D8A8Y, 85336S, 1:400), CD14 (Abcam, ab182032, 1:400), CD68 (CST, D4B9C, 76437S, 1:400), CD11b (CST, D6X1N, 49420S, 1:200), CD11c (Abcam, ab52632, 1:200), CD20 (Abcam, ab64088, 1:200), Foxp3 (CST, D2W8E, 98377S, 1:100), CD56 (CST, 123C3, 3576S, 1:100), TIM-3 (CST, D5D5R, 45208S, 1:50), TIGIT (Abcam, ab243903, 1:200), PD-1 (CST, EH33, 43248S, 1:100). Slides were scanned at ×20 magnification on Nano Zoomer Digital Pathology scanner (Hamamatsu) and analyzed using Aperio Digital Pathology software (Leica).

#### Spatial transcriptomics data generation

We conducted spatial transcriptomics using Visium technology from 10x Genomics on tissue samples from patients #41, #56, #59, and #65. RNA was carefully extracted from 10 μm sections of formalin-fixed paraffin-embedded (FFPE) tissue using the Qiagen RNeasy FFPE Kit. The quality of the extracted RNA was evaluated using the Agilent RNA 6000 Pico Kit, selecting blocks with a DV200 value above 50% for further analysis. Each tissue section was precisely positioned within the capture area of a Visium Spatial Gene Expression Slide (PN-1000188, 10x Genomics). The tissue sections underwent deparaffinization, staining, and decrosslinking, followed by hybridization, ligation, release, and extension processes. Spatial gene expression libraries from FFPE samples were constructed using the Visium Human Transcriptome Probe Kit (PN-1000363) and the Visium FFPE Reagent Kit (PN-1000363), following the prescribed protocols. These libraries were sequenced using the Illumina NovaSeq 6000 systems, targeting a minimum of 75,000 read pairs per spot and the detection of approximately 2,000 genes per spot. Subsequent analysis was carried out using the Scanpy package in python.

### Quantification and statistical analysis

All the statistical analyses were conducted using GraphPad Prism v.8.0 (GraphPad Software, Inc., San Diego, CA, USA) and R version 4.1.2 (R Foundation for Statistical Computing, Vienna, Austria). Statistical parameters were reported in the figure legend. Continuous variables were expressed as median (range) and compared between the two groups using the Student’s t test or Mann-Whitney test. Categorical variables were expressed as frequency (percentage) and compared between the two groups using chi-square test or Fisher exact test. Survival was estimated using the Kaplan-Meier method and *p* value was calculated through log-rank test. All reported *p*-value were 2-tailed, and the statistical significance was defined as *p* < 0.05.

### Additional resources

This clinical trial has been registered on https://clinicaltrials.gov (NCT04649476).

## Supplemental information

Document S1. Figures S1–S11 and Method S1

Table S1. Surgical procedures of patients in arm Cam and Cam+TPF, related to Figure 2

Table S2. Detailed information of surgical procedures of patients in arm Cam and Cam+TPF, related to Figure 2

Table S3. High-risk pathology features of patients in arm Cam and Cam+TPF, related to Figure 2 and Figure 3

Table S4. Type of adjuvant therapy in arm Cam and Cam+TPF, related to Figure 2 and Figure 3

Table S5. Tumor responses of patients, related to Figure 2 and Figure 3

Table S6. Clinical to pathological downstaging post neoadjuvant therapy, related to Figure 2 and Figure 3

Table S7. Surgery-related adverse events (clavien-dindo) in 66 enrolled participants, related to Table 2

Table S8. Correlation between clinical factors and response (arm Cam), related to Figure 2 and Figure 3

Table S9. Correlation between immunohistochemical indexes and response (arm Cam), related to Figure 2 and Figure 3

Document S2. Article plus supplemental information
