# Supplementary material for: Neoadjuvant immunotherapy with or without chemotherapy in locally advanced oral squamous cell carcinoma: Randomized, two-arm, phase 2 trial
Source: Cell Rep Med. 2025 Jan 30;6(2):101930. doi: 10.1016/j.xcrm.2025.101930 (PMC11866509; doi:10.1016/j.xcrm.2025.101930)
Supplement: Document S2. Article plus supplemental information [file mmc11.pdf]

Cell Reports
Medicine

Article

Neoadjuvant immunotherapy with or without chemotherapy in locally advanced oral squamous cell carcinoma: Randomized, two-arm, phase 2 trial

Graphical abstract

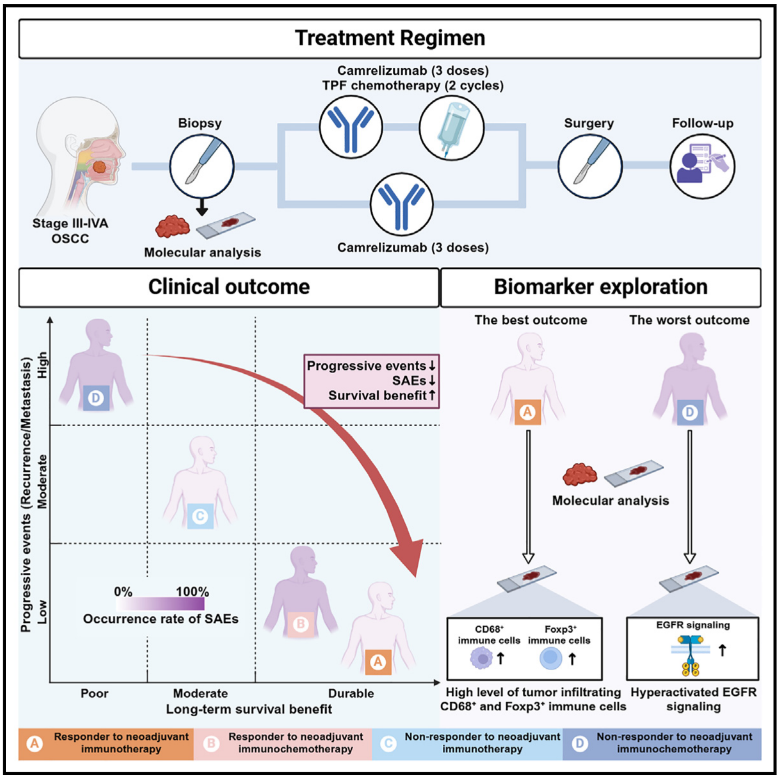

Highlights

- Neoadjuvant camrelizumab plus TPF chemotherapy are feasible in locally advanced OSCC
- The combination regimen is well tolerated and associated with durable survival benefits
- Pathological partial response is a surrogate primary endpoint for neoadjuvant therapy
- Non-responders to neoadjuvant immunochemotherapy necessitate close monitoring

Authors

Hai-Ming Liu, Xue-Peng Xiong, Zi-Li Yu, ..., Jun Jia, Bing Liu, Gang Chen

Correspondence

wzhang88@whu.edu.cn (W.Z.),
junjia@whu.edu.cn (J.J.),
liubing9909@whu.edu.cn (B.L.),
geraldchan@whu.edu.cn (G.C.)

In brief

Patients with locally advanced oral squamous cell carcinoma often experience poor outcomes with standard care. Liu et al. conduct a randomized, two-arm, phase 2, non-comparative trial to evaluate the feasibility and safety profile of neoadjuvant camrelizumab, either alone or in combination with TPF chemotherapy, in patients with resectable locally advanced OSCC.

Liu et al., 2025, Cell Reports Medicine *6*, 101930
February 18, 2025 © 2025 The Author(s). Published by Elsevier Inc.
https://doi.org/10.1016/j.xcrm.2025.101930

CellPress

Cell Reports Medicine

CellPress
OPEN ACCESS

Article

Neoadjuvant immunotherapy with or without chemotherapy in locally advanced oral squamous cell carcinoma: Randomized, two-arm, phase 2 trial

Hai-Ming Liu,[1,9] Xue-Peng Xiong,[1,2,9] Zi-Li Yu,[1,2,9] Zhe Shao,[1,2,9] Gai-Li Chen,[3,9] Yu-Tong Liu,[1] Xin-Xin Wang,[1] Qiu-Yun Fu,[1] Xiao-Xia Cheng,[2] Jing Li,[2] Jia-Li Zhang,[1,4] Bo Li,[1,5] Hong-Yun Gong,[6] Ya-Hua Zhong,[3] Wei Zhang,[1,2,*] Jun Jia,[1,2,*] Bing Liu,[1,2,*] and Gang Chen[1,2,7,8,10,*]

[1]State Key Laboratory of Oral & Maxillofacial Reconstruction and Regeneration, Key Laboratory of Oral Biomedicine Ministry of Education, Hubei Key Laboratory of Stomatology, School & Hospital of Stomatology, Wuhan University, Wuhan 430079, China
[2]Department of Oral and Maxillofacial Surgery, School and Hospital of Stomatology, Wuhan University, Wuhan 430079, China
[3]Department of Radiation and Medical Oncology, Hubei Key Laboratory of Tumor Biological Behaviors, Hubei Cancer Clinical Study Center, Zhongnan Hospital of Wuhan University, Wuhan 430071, China
[4]Department of Oral Pathology, School and Hospital of Stomatology, Wuhan University, Wuhan 430079, China
[5]Department of Oral Radiology, School and Hospital of Stomatology, Wuhan University, Wuhan 430079, China
[6]Cancer Center, Renmin Hospital of Wuhan University, Wuhan 430060, China
[7]TaiKang Center for Life and Medical Sciences, Wuhan University, Wuhan 430071, China
[8]Frontier Science Center for Immunology and Metabolism, Wuhan University, Wuhan 430071, China
[9]These authors contributed equally
[10]Lead contact
*Correspondence: wzhang88@whu.edu.cn (W.Z.), junjia@whu.edu.cn (J.J.), liubing9909@whu.edu.cn (B.L.), geraldchan@whu.edu.cn (G.C.)
https://doi.org/10.1016/j.xcrm.2025.101930

SUMMARY

Patients with locally advanced oral squamous cell carcinoma (OSCC) have poor outcomes with standard care. Neoadjuvant therapy is shown to be effective for these patients. In the randomized, two-arm, phase 2, non-comparative trial, we investigate the efficacy and safety of the neoadjuvant programmed cell death 1 (PD-1) inhibitor camrelizumab with or without docetaxel-cisplatin-5-fluorouracil (TPF) chemotherapy in patients with resectable locally advanced OSCC. Patients with stage III–IVA OSCC receive neoadjuvant therapy with three cycles of camrelizumab (arm Cam) with or without two cycles of TPF chemotherapy (arm Cam+TPF), followed by surgery and adjuvant therapy. Major pathological response (MPR) is achieved in both arm Cam (5/34, 14.7%) and arm Cam+TPF (26/34, 76.4%). With a median follow-up of 32 months, the 2-year event-free survival (EFS) rate of arm Cam and Cam+TPF is 52.9% and 91.2%, respectively. This work demonstrates feasibility and safety for immunochemotherapy in the neoadjuvant setting for OSCC. This study was registered at ClinicalTrials.gov (NCT04649476).

INTRODUCTION

Oral squamous cell carcinoma (OSCC), a prevalent subset of head and neck squamous cell carcinoma (HNSCC), is diagnosed with locally advanced disease in more than 60% of patients.[1] Surgical resection followed by adjuvant radiotherapy with or without chemotherapy has been recommended as the standard of care for patients with locally advanced OSCC. However, the prognosis of this subset of patients remains poor, with a high risk of recurrence or metastasis. Their 5-year overall survival (OS) rate is still less than 50%.[2–5] A novel therapeutic modality is needed for these patients.

Neoadjuvant therapy, designed to reduce the burden of locoregional disease before surgery, has emerged as a promising treatment strategy. Research indicates that neoadjuvant chemotherapy can elicit pathological responses in patients with locally advanced OSCC. Specifically, major pathological response (MPR) rates have been reported at 27.7% for neoadjuvant docetaxel-cisplatin-5-fluorouracil (TPF) and 33% for cisplatin-5-fluorouracil (PF) chemotherapy. Impressive clinical regression rates of 80.6% and 82% have been observed for TPF and PF chemotherapy, respectively, in patients with locally advanced OSCC.[6,7] However, it is noteworthy that no significant difference in survival advantage has been identified between patients who received neoadjuvant chemotherapy and those who did not, indicating that current neoadjuvant chemotherapy regimens may not enhance long-term survival outcomes.

Recent advancements in immune checkpoint inhibitors have shown promising results in the management of recurrent and metastatic HNSCC, including OSCC.[8] The successes have prompted investigations into their potential use in the neoadjuvant setting. Clinical trials have demonstrated the efficacy and safety of neoadjuvant mono-immunotherapy in HNSCC, using agents such as pembrolizumab[9,10] and nivolumab.[11–13] Reported major pathological response rates for pembrolizumab range from 5% to 7%, while those for nivolumab range from

© 2025 The Author(s). Published by Elsevier Inc.
This is an open access article under the CC BY license (http://creativecommons.org/licenses/by/4.0/).

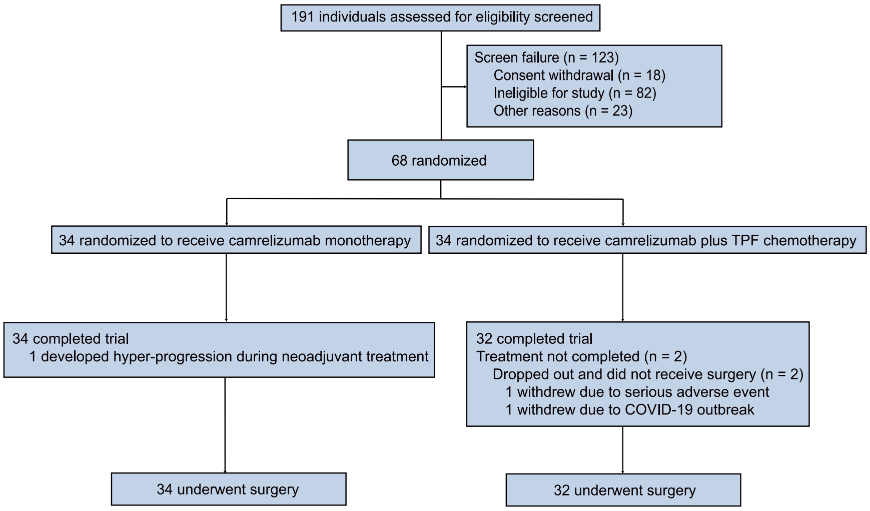

Figure 1. Trial flow diagram

A total of 68 patients were enrolled in this trial, with 34 patients completing the full treatment protocol in arm Cam, and 32 patients completing the full treatment protocol in arm Cam+TPF.

5.9% to 17%. Despite these relatively low MPR rates, long-term outcomes appear favorable. For instance, Uppaluri et al. demonstrated that neoadjuvant pembrolizumab therapy decreased the 1-year relapse rate among patients with locally advanced human papilloma virus (HPV)-unrelated HNSCC with high-risk pathology (positive margins and/or extranodal extension).[9] Additionally, Wise-Draper et al. found that patients who achieved a pathological response to neoadjuvant pembrolizumab therapy had significantly improved disease-free survival (DFS) rates in the first year (93% vs. 72%).[10] Knochelmann et al. reported a 2-year OS rate of 83% among 12 patients with resectable stage II-IVA OSCC.[11] Ferris et al. reported 2-year recurrence-free survival rates of 88.2% for patients with HPV-positive HNSCC (stage III–IV),[12] while Schoenfeld et al. reported 1-year progression-free survival and OS rates of 85% and 89% for patients with stage II–IVA OSCC, respectively.[13] Subsequently, studies have been dedicated to enhancing the MPR rate by combining neoadjuvant immunotherapy with other treatment regimens including durvalumab plus tremelimumab,[14] nivolumab plus ipilimumab,[13,15] and nivolumab plus lirilumab.[16] A recent study reported that the combination of anti-programmed cell death 1 (PD-1) and anti-angiogenesis therapy in locally advanced OSCC improved the MPR rate to 40%.[17] This highlights the need to explore additional approaches to enhance the MPR rate of neoadjuvant immunotherapy.

In addition to the direct cytotoxic effects on cancer cells, emerging evidence indicated that chemotherapy may also modulate the immune system, including weakening the activity of T regulatory cells and enhancing the presentation of tumor antigens, suggesting a possibility for the combination of neoadjuvant immunotherapy and chemotherapy. Yang et al. recently reported an MPR rate of 76.0% in 27 patients with locally advanced HNSCC (93.3% oropharyngeal squamous cell carcinoma [OPSCC] and 6.7% OSCC).[18] In single-arm phase 1 and phase 2 trial, the MPR rate was reported as 60% and 63% when the combination of anti-PD-1 (toripalimab or camrelizumab) and chemotherapy (albumin paclitaxel and cisplatin) was administered in patients with locally advanced HNSCC.[19,20] However, another single-arm phase 1b clinical trial has reported that the combination of anti-PD-1 (toripalimab) and chemotherapy (gemcitabine and cisplatin) achieved an MPR rate of only 44.5% in 18 patients with locally advanced HNSCC (30.3% OPSCC and 69.7% OSCC).[21] These findings highlight variability in the efficacy of different neoadjuvant immunotherapy and chemotherapy combinations and suggest that response may differ between OSCC and OPSCC. Therefore, further evidence is needed to substantiate the efficacy of neoadjuvant immunochemotherapy in locally advanced OSCC.

Camrelizumab is a fully humanized anti-PD-1 monoclonal antibody that binds to a different site on PD-1 compared to nivolumab and pembrolizumab.[22–24] Its efficacy as a monotherapy in HNSCC remains unexplored. In this study, we report a randomized, two-arm, phase 2, non-comparative trial of neoadjuvant PD-1 inhibitor camrelizumab alone (arm Cam) or combined with TPF chemotherapy (arm Cam+TPF), followed by surgery and adjuvant therapy in 68 patients with stage III–IVA OSCC. The primary objectives are to assess the efficacy, survival benefit, and safety profiles of neoadjuvant immunochemotherapy and immunotherapy in patients with locally advanced OSCC.

RESULTS

Patient characteristics

From March 2021 to July 2022, 191 patients were assessed for eligibility, and 68 evaluable patients were enrolled (Figure 1; Figure S1). Thirty-four patients were randomized to arm Cam and 34 to arm Cam+TPF. The baseline characteristics of the 68 enrolled patients are summarized in Table 1. The average age was 50.7 years (range, 32–68 years), and the majority of patients were male (59, 87%), current or former smokers (46, 68%), and had Eastern Cooperative Oncology Group performance status of 0 (44, 65%). The most common primary tumor site was tongue (36, 53%). Thirty-two patients (47%) had clinically or radiographically node-positive disease, and 29 patients (43%) had stage IVA disease (pre-therapy). Programmed cell death-ligand 1 (PD-L1) combined positive score (CPS) at baseline was 1%–19% in 30 patients (44%) and 20% or higher in 9 patients (13%).

Treatment characteristics

Among the 68 enrolled patients, 33 patients completed full treatment protocol in arm Cam, and 32 patients completed full treatment protocol in arm Cam+TPF. One patient in arm Cam received two cycles of neoadjuvant immunotherapy but experienced

Table 1. Baseline characteristics of the 68 enrolled patients

Characteristic	Overall (*n* = 68)	Arm Cam Camrelizumab (*n* = 34)	Arm Cam + TPF Camrelizumab + TPF (*n* = 34)
Age, mean (range)	50.7 (32.0–68.0)	52.0 (32.0–67.0)	49.5 (33.0–68.0)
Gender, *n* (%)			
Male	59 (86.8)	28 (82.4)	31 (91.2)
Female	9 (13.2)	6 (17.6)	3 (8.8)
Smoking history, *n* (%)			
Current or former	46 (67.6)	24 (70.6)	22 (64.7)
Never	22 (32.4)	10 (29.4)	12 (35.3)
Alcohol use history, *n* (%)			
Current or former	33 (48.5)	15 (44.1)	18 (52.9)
Never	35 (51.5)	19 (55.9)	16 (47.1)
ECOG performance status, *n* (%)			
0	44 (64.7)	18 (52.9)	26 (76.5)
1	24 (35.3)	16 (47.1)	8 (23.5)
Tumor site, *n* (%)			
Oral tongue	36 (52.9)	18 (52.9)	18 (52.9)
Gingiva	8 (11.8)	5 (14.7)	3 (8.8)
Floor of mouth	8 (11.8)	4 (11.8)	4 (11.8)
Buccal mucosa	16 (23.5)	7 (20.6)	9 (26.5)
Clinical T-stage[a], *n* (%)			
T2	15 (22.1)	6 (17.6)	9 (26.5)
T3	37 (54.4)	19 (55.9)	18 (52.9)
T4	16 (23.5)	9 (26.5)	7 (20.6)
Clinical N-stage[a], *n* (%)			
N0	36 (53.0)	23 (67.6)	13 (38.2)
N1	16 (23.5)	6 (17.7)	10 (29.4)
N2	16 (23.5)	5 (14.7)	11 (32.4)
Clinical disease stage, *n* (%)			
III	39 (57.4)	21 (61.8)	18 (52.9)
IVA	29 (42.6)	13 (38.2)	16 (47.1)
PD-L1 combined positive score, *n* (%)			
<1	29 (42.6)	16 (47.1)	13 (38.2)
1–19	30 (44.2)	12 (35.3)	18 (53.0)
≥20	9 (13.2)	6 (17.6)	3 (8.8)

Abbreviation: ECOG, Eastern Cooperative Oncology Group
[a]American Joint Committee on Cancer, 8th Edition staging.

disease hyperprogression. Although this patient underwent surgical treatment, he developed distant metastasis and passed away 5 months after surgery. In arm Cam+TPF, two patients did not complete the full treatment protocol. One received one cycle of camrelizumab plus TPF chemotherapy and dropped out due to serious adverse event. The other patient received one cycle of camrelizumab but declined further treatment due to the COVID-19 outbreak, subsequently undergoing surgery and receiving postoperative radiotherapy. This patient remains alive with no signs of tumor recurrence or metastasis. Following neoadjuvant therapy, 66 patients underwent radical resection of the primary tumor along with neck lymph node dissection. The surgical procedures for patients in both arm Cam and arm Cam+TPF are detailed in Tables S1 and S2. Notably, the types of surgical procedures for the Cam+TPF group were not altered according to therapeutic effects. The proportions of patients undergoing glossectomy, mandibular resection, neck dissection, and flap reconstruction were generally consistent between the two arms. Three patients in arm Cam+TPF, including two with cancer involving the bilateral floor of the mouth and one with cancer involving the left mandibular gingiva, underwent extensive segmental mandibulectomy and received fibular myocutaneous flap reconstruction.

60 patients received postoperative risk-adaptive adjuvant therapy within 4–6 weeks after surgery. Among them, 44 patients (20 in arm Cam and 24 in arm Cam+TPF) received adjuvant radiotherapy. Additionally, 11 patients in arm Cam (5 with extracapsular extension, 3 with poorly differentiated pathology, and 3 with facial invasion) and 5 patients in arm Cam+TPF (3 with extracapsular extension and 2 with facial invasion) received concurrent radiotherapy with platinum-based chemotherapy. Three patients in arm Cam+TPF who had extracapsular extension rejected to receive postoperative adjuvant therapy (see Tables S3 and S4). Although patients with high-risk pathological factors received intensified postoperative adjuvant therapy, a relatively high incidence of treatment failure (recurrence/metastasis/death) was still observed.

Efficacy outcomes

Figures 2A and 2C depict representative examples of two patients with T3N2bM0 and T3N0M0 OSCC involving the tongue in arm Cam (top) and arm Cam+TPF (bottom), respectively. These images demonstrate striking responses to the respective treatment regimens. The waterfall plots of radiological and pathological response for individual patients in arm Cam and arm Cam+TPF are shown in Figures 2B and 2D. As shown in Table S5, among the intention-to-treat population, 5 patients (5/34, 14.7%, 95% confidence interval [CI] 6.4%–30.1%) achieved MPR, and no patients achieved pathological complete response (pCR) in arm Cam. In arm Cam+TPF, the MPR was observed in 26 out of 34 patients (76.4%, 95% CI 60.0%–87.5%), including 10 patients (29.4%) who achieved pCR. The R0 resection rate was 100% in both arm Cam and arm Cam+TPF.

Regarding radiological response, we evaluated both primary tumor and lymph node metastasis. In arm Cam, 3 patients (8.8%) achieved a partial response (PR) according to the Response Evaluation Criteria in Solid Tumors (RECIST), while in arm Cam+TPF, 16 patients (47.0%) achieved PR. The overall response rates of these metastatic lymph nodes (LNs) in arm Cam and Cam+TPF were both 2.9%. Notably, the clinical response rates for these LNs were poorer compared to the primary tumor. A correlation between pathological and radiological response rates was observed (Figure S2A). Specifically, all patients with progressive disease (PD as per RECIST) corresponded to pathological non-response (pNR), and all patients achieved PR (PR as per RECIST) corresponded to pathological PR (pPR), MPR, and pCR. However, patients who achieved

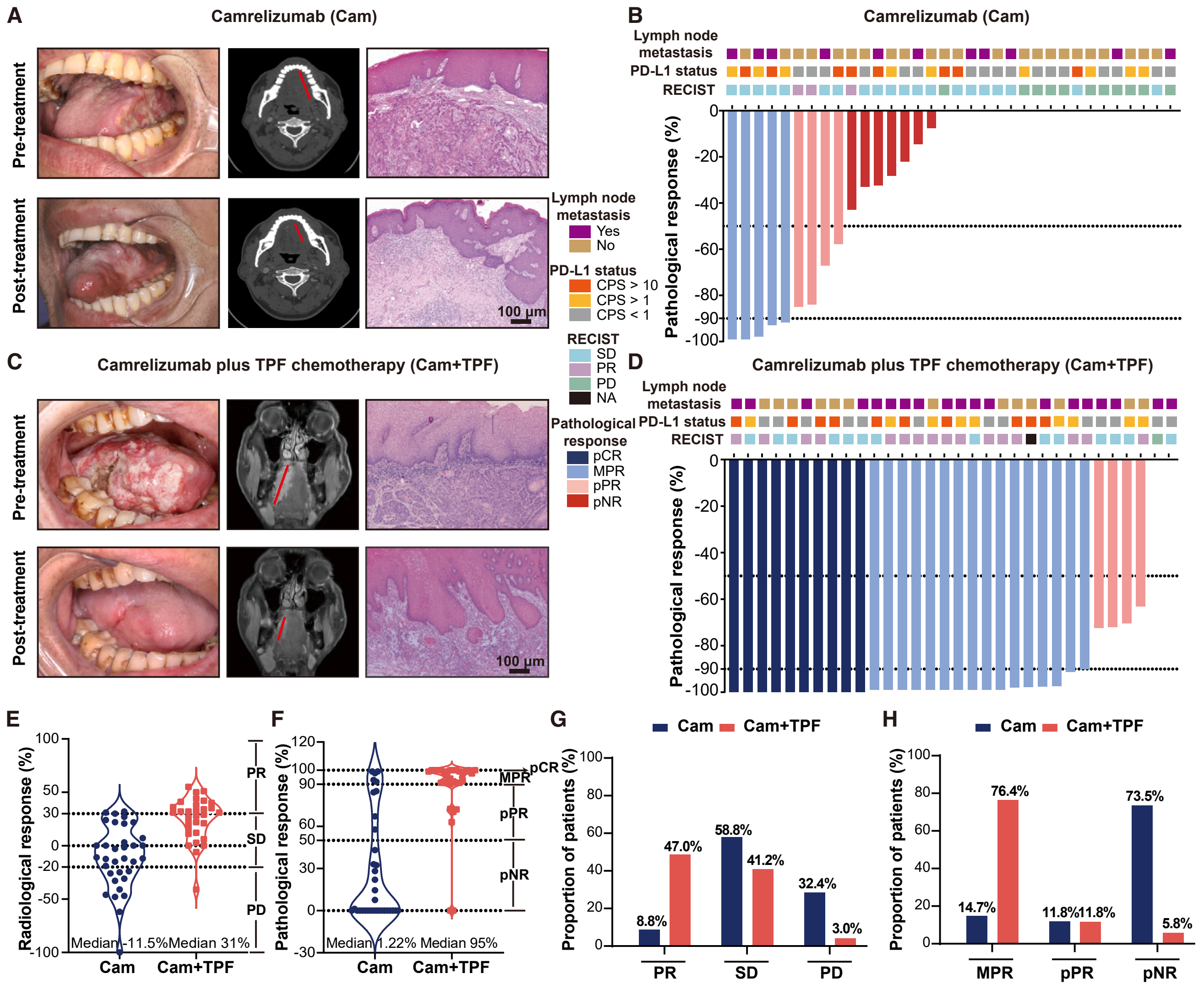

Figure 2. Patients' tumor response in the subgroups

(A) Major pathological response in a 53-year-old man (T3N2bM0) who received neoadjuvant camrelizumab mono-immunotherapy. Images of the left tongue (left), radiological images (middle), and pathological images (right) before (top) and after (bottom) neoadjuvant therapy were shown; scale bar, 100 μm; red line indicates the longest diameter of the tumor.

(B) Waterfall plot showing patients' pathological response and radiological response in arm Cam.

(C) Major pathological response in a 56-year-old man (T3N0M0) who received neoadjuvant camrelizumab immunotherapy plus TPF chemotherapy. Images of the right tongue (left), radiological images (middle), and pathological images (right) before (top) and after (bottom) neoadjuvant therapy were shown; scale bar, 100 μm; red line indicates the longest diameter of the tumor.

(D) Waterfall plot showing patients' pathological response and radiological response in arm Cam+TPF.

(E) Percentage of radiological response rate in resected tumor specimens. Median percentage: arm Cam −11.5% (range −100% to 32%) and arm Cam+TPF 31% (range −41% to 55%).

(F) Percentage of pathological response rate in resected tumor specimens. Median percentage: arm Cam 1.22% (range 0%–99%) and arm Cam+TPF 95% (range 0%–100%).

(G) Proportion of patients with radiological response in the subgroups.

(H) Proportion of patients with pathological response in the subgroups. CPS, combined positive score; PR, partial response; SD, stable disease; PD, progressive disease; NA, not applicable; pCR, pathological complete response; MPR, major pathological response; pPR, pathological partial response; pNR, pathological non-response.

stable disease (SD as per RECIST) corresponded to all types of pathological response including pNR, pPR, MPR, and pCR, indicating a wide range of pathological responses in patients with SD on radiological imaging (Figure S2B).

These data indicate that neoadjuvant camrelizumab combined with TPF chemotherapy exhibited a superior radiological and pathological response rate (Figures 2E and 2F; Table S5). Subgroup analysis revealed that majority of patients receiving

Table 2. Treatment-related adverse events (common terminology criteria for adverse events version 5.0) in 68 enrolled participants

	Arm Cam (n = 34) n (%)			Arm Cam + TPF (n = 34) n (%)		
	Grade 1 or 2	Grade 3	Grade 4	Grade 1 or 2	Grade 3	Grade 4
RCCEP	29 (85)	0	0	22 (65)	0	0
Anemia	7 (21)	0	0	23 (68)	0	0
Mucositis oral	6 (18)	0	0	8 (24)	0	0
Creatinine increased	5 (15)	0	0	17 (50)	0	0
Lymphopenia	4 (12)	0	0	16 (47)	3 (9)	0
GGT increased	4 (12)	0	0	6 (18)	0	0
ALT increased	3 (9)	1 (3)	0	12 (35)	0	0
Lactate dehydrogenase increased	2 (6)	0	0	10 (29)	0	0
AST increased	2 (6)	0	0	8 (24)	0	0
Neutrophilia	2 (6)	0	0	0	0	0
Diarrhea	1 (3)	0	0	8 (24)	0	1 (3)
Leukocytosis	1 (3)	0	0	0	0	0
Creatine kinase increased	0	1 (3)	0	10 (29)	0	0
Leukopenia	0	0	0	9 (26)	4 (12)	1 (3)
Neutropenia	0	0	0	7 (21)	4 (12)	3 (9)
Thrombocytopenia	0	0	0	3 (9)	0	0
Monocyte decreased	0	0	0	2 (6)	0	0
Monocyte increased	0	0	0	1 (3)	0	0
Red blood cell decreased	0	0	0	2 (6)	0	0
Alopecia	0	0	0	26 (76)	0	0
Vomiting	0	0	0	10 (29)	0	0
Creatine kinase isoenzyme increased	0	0	0	1 (3)	0	0
Diminution of vision	0	0	0	1 (3)	0	0

Abbreviation: RCCEP, reactive cutaneous capillary endothelial proliferation; GGT,gamma-glutamyl transpeptidase; ALT, alanine aminotransferase; AST, aspartate aminotransferase.

this combination therapy achieved PR and MPR (Figures 2G and 2H). As shown in Table S6, most patients in arm Cam did not experience significant changes in tumor staging, while 27 patients (79%) in the arm Cam+TPF experienced clinical to pathological downstaging post neoadjuvant therapy. It was observed that patients with positive PD-L1 expression (PD-L1 CPS > 1) had higher response rates in both two arms or in arm Cam, but the difference was not significant (Figures S3A and S3B).

Safety

Treatment-related adverse events (TRAEs) observed in the study are summarized in Table 2. In arm Cam, the most common TRAEs of any grade were reactive cutaneous capillary endothelial proliferation (RCCEP, 29, 85%), followed by anemia (7, 21%). In arm Cam+TPF, the most common TRAEs were alopecia (26, 76%), followed by anemia (23, 68%) and RCCEP (22, 65%). Two patients (6%) in arm Cam experienced grade 3–4 TRAEs. Eight patients (23%) in arm Cam+TPF experienced grade 3–4 TRAEs, with six of these patients having more than one grade 3–4 TRAEs. The grade 3–4 TRAEs observed in arm Cam+TPF were neutropenia (7, 21%), leukopenia (5, 15%), lymphopenia (3, 9%), and diarrhea (1, 3%). One patient in arm Cam+TPF experienced multiple episodes of vomiting during neoadjuvant treatment, and this cascade of events ultimately led to cardiac insufficiency, followed by anuria. Unfortunately, the patient subsequently passed away due to multiple organ dysfunction. Surgery-related adverse events were reported for 6 of 34 patients (17.6%) in arm Cam and 5 of 32 patients (15.6%) in arm Cam+TPF, most of which were grade 1 or 2 (Table S7). The most frequent surgery-related adverse events were fever (9%) in arm Cam and flap swelling (9%) in arm Cam+TPF. Two patients (1 in arm Cam and 1 in arm Cam+TPF) experienced flap vascular crisis after surgery. These results collectively indicated that both neoadjuvant camrelizumab alone and neoadjuvant camrelizumab plus TPF chemotherapy were considered feasible in patients with locally advanced OSCC.

Survival outcomes

At the time of data analysis, the median follow-up time was 32 months. From 2.5 months to 28.1 months after treatment initiation in arm Cam, 4 patients (4/34, 11.7%) experienced locoregional recurrences, and 1 patient succumbed to the disease. Four patients (4/34, 11.7%) experienced distant metastasis and succumbed to the disease. In arm Cam+TPF, 2 patients (2/34, 5.8%) experienced distant metastasis within 6–8 months after treatment initiation, and one of them later died (Figure 3A). Another patient in arm Cam+TPF died because of multiple organ dysfunction before surgery. The 2-year OS rate in arm Cam and Cam+TPF was 91.2% (95% CI 82.1%–100.0%) and 94.1% (95% CI 86.5%–100.0%) (Figure 3B), respectively. The 2-year DFS

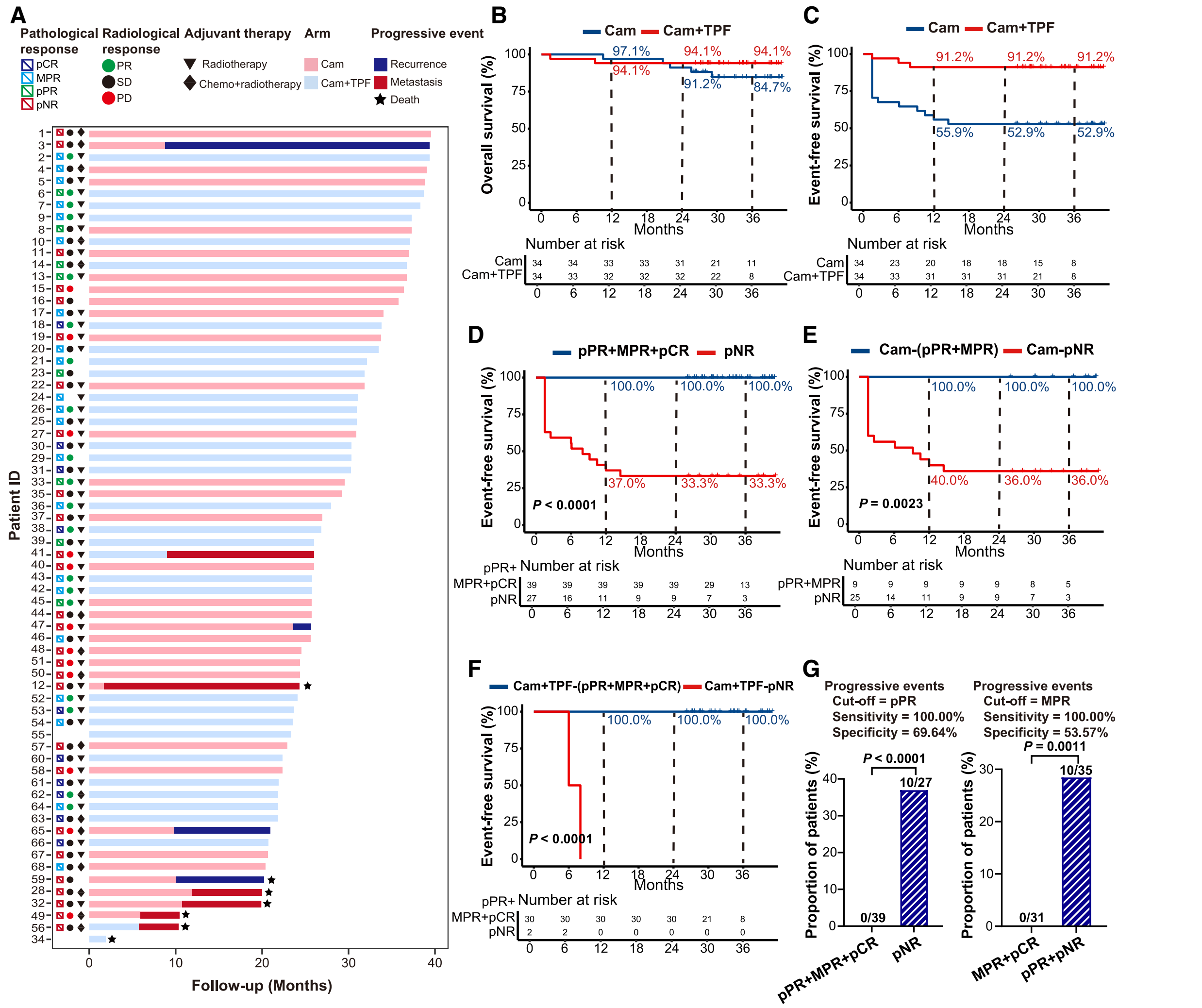

Figure 3. Follow-up and survival outcomes of patients

(A) Swimmer plot showing patients' survival outcomes.

(B) Kaplan-Meier curve of overall survival for the patients in arm Cam and arm Cam+TPF.

(C) Kaplan-Meier curve of event-free survival for the patients in arm Cam and arm Cam+TPF.

(D) Kaplan-Meier curve of event-free survival for the pathological responders (pPR+MPR+pCR) and non-responders (pNR). Log rank *p* value was used to estimate the significance among groups.

(E) Kaplan-Meier curve of event-free survival for the pathological responders (pPR+MPR) and non-responders (pNR) in arm Cam. Log rank *p* value was used to estimate the significance among groups.

(F) Kaplan-Meier curve of event-free survival for the pathological responders (pPR+MPR+pCR) and non-responders (pNR) in arm Cam+TPF. Log rank *p* value was used to estimate the significance among groups.

(G) Proportion of patients who had progressive events in different subgroups. *p* value was determined by two-sided Fisher's exact test. PR, partial response; SD, stable disease; PD, progressive disease; pCR, pathological complete response; MPR, major pathological response; pPR, pathological partial response; pNR, pathological non-response.

rate and 2-year event-free survival (EFS) rate in arm Cam+TPF were 93.9% (95% CI 86.1%–100.0%) and 91.2% (95% CI 82.1%–100.0%) (Figures S4A and 3C). These data demonstrate that the incorporation of TPF chemotherapy with camrelizumab in arm Cam+TPF led to durable survival benefits in patients with locally advanced OSCC.

It was noticed that all the patients experiencing progressive events were pathological non-responders (Figure 3A). Consistently, the comparative analysis of outcomes among patients achieving different pathological responses revealed that those who pathologically responded to neoadjuvant immunotherapy or immunochemotherapy (pPR or MPR, pCR) had significantly higher EFS rates compared to non-responders (pNR) (Figure S4B). It was further observed that if pPR was set as the cutoff value, patients who achieved a pathological response (pPR or MPR, pCR) had a significant improvement in EFS (Figure 3D). By virtue of the two-arm design, we found that patients with pPR, either in arm Cam (Figures 3E and S4C) or arm Cam+TPF

CellPress
OPEN ACCESS

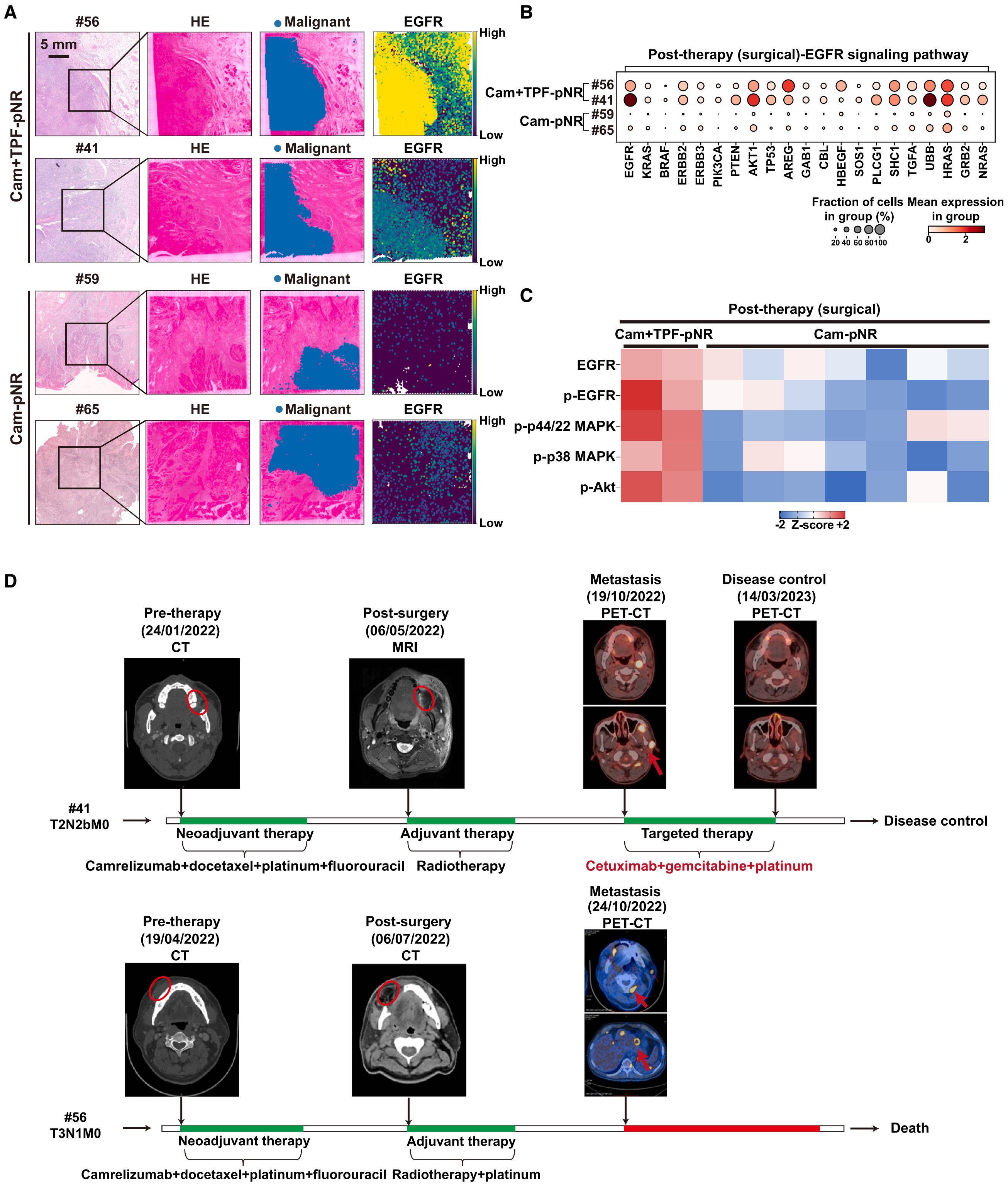

Figure 4. Worse prognosis of non-responders in arm Cam+TPF

(A) Spatial heatmaps showing expression intensity of *EGFR*, including Cam+TPF-pNR group (n = 2) and Cam-pNR group (n = 2); scale bar, 5 mm.

(B) Dot plot showing expression levels of genes related to EGFR signaling pathway, including Cam+TPF-pNR group (n = 2) and Cam-pNR group (n = 2).

(legend continued on next page)

(Figures 3F and S4D), had similar EFS to patients achieving MPR in the first 3 years. These findings suggest that a pathological response (pCR, MPR, and pPR) to neoadjuvant therapy leads to a significant improvement in EFS. Notably, further analysis revealed that using pPR as the optimal cutoff value resulted in the same predictive performance in event probability between the two divided populations compared with MPR (Figure 3G). According to RECIST criteria, patients who suffered PD all had a terrible prognosis, while patients who achieved PR had a favorable prognosis (Figure S5A). As aforementioned, patients who achieved SD may have distinct pathological responses, so it was not sufficient enough to confer long-term survival benefit (Figure S5B). According to PD-L1 expression, patients seemed to benefit from positive PD-L1 expression in term of EFS, but the difference was not significant (Figures S6A and S6B). Taken together, these results demonstrate that pathological responders tend to have better EFS compared to non-responders. Additionally, achieving a pPR to neoadjuvant immunotherapy or immunochemotherapy may serve as a surrogate primary study endpoint, as it shows the same predictive value as MPR in patients with locally advanced OSCC.

High metastatic rate and poor prognosis of non-responders to neoadjuvant immunochemotherapy

Notably, there were still a few individuals who exhibited resistance to neoadjuvant immunochemotherapy. Even worse, non-responders in arm Cam+TPF suffered an extremely higher risk of postoperative metastasis (2/2, 100%). Moreover, non-responders in arm Cam+TPF suffered even worse prognosis compared with patients who received standard of care in terms of DFS (2-year DFS 0% vs. 63%–73%[15,25]) and OS (2-year OS 50% vs. 60%–68%[6,25,26]). Therefore, our study emphasizes the clinical importance of close monitoring and early intervention of the non-responders who underwent neoadjuvant immunochemotherapy in case of progressive events.

To investigate the underlying mechanisms and identify potential signatures of non-responders to neoadjuvant immunochemotherapy, we performed an analysis using spatial transcriptomics. The results revealed elevated epidermal growth factor receptor (EGFR) signaling pathway at the tumor borders, primarily in tumor cells, in the two non-responders (#41 and #56) in arm Cam+TPF compared to the non-responders (#59 and #65) in arm Cam (Figures 4A and 4B). Consistently, strong staining of EGFR, phosphorylated EGFR (p-), and downstream molecules were observed in both the biopsy and surgical specimens (Figures S7 and S8). Further quantitative analysis showed that the aforementioned molecules were obviously overexpressed in non-responders in arm Cam+TPF compared with arm Cam before and after the therapy (Figures 4C and S9).

However, the two non-responders in arm Cam+TPF experienced distinctive prognosis due to the difference in following treatment. After being diagnosed with metastasis after surgery, patient #41 received the therapy of cetuximab plus gemcitabine and platinum. Strikingly, the size of metastatic lesion rapidly reduced, and the disease was effectively controlled. However, patient #56 refused to receive salvage therapy and unfortunately passed away (red circle indicates primary lesion and red arrow indicates metastatic lesion) (Figure 4D). These aforementioned results suggest that EGFR hyperactivation may play an important role in resistance to neoadjuvant immunochemotherapy.

Decision-making strategy

To facilitate pre-treatment screening of patients toward precision immunotherapy in the future, the patients in our clinical trial were classified into four groups based on their long-term survival, progressive events (recurrence/metastasis), and severe adverse events (grade 3–5 adverse events, SAEs) (Figure S10): A, responders to neoadjuvant immunotherapy; B, responders to neoadjuvant immunochemotherapy; C, non-responders to neoadjuvant immunotherapy; and D, non-responders to neoadjuvant immunochemotherapy. Among the four groups of patients, we think group A is the most preferred, followed by group B, while group D is the most undesired. An ideal screening process is that we first select patients who might respond to neoadjuvant mono-immunotherapy (group A), and then the remaining patients all turn into neoadjuvant immunochemotherapy, but before that, we should exclude the potential non-responders to neoadjuvant immunochemotherapy (group D). For this purpose, we conducted exploratory subgroup analyses to find the potential signatures of patients in group A according to baseline clinical characteristics and a panel of checkpoint molecules and immunocytes examined in pre-therapy biopsy tissues. There was no significant difference between groups based on clinical characteristics (Table S8). Patients with a high infiltration level of $CD68^+$ cells and $Foxp3^+$ cells in tumor tissues are likely to benefit from neoadjuvant mono-immunotherapy (Table S9). As aforementioned, patients with hyperactivated EGFR are potential non-responders to neoadjuvant immunochemotherapy.

DISCUSSION

In this study, we reported a randomized, two-arm clinical trial exploring the efficacy and safety of neoadjuvant immunochemotherapy and neoadjuvant immunotherapy in patients with locally advanced OSCC. Consistent with previous studies, neoadjuvant immunotherapy was effective in only a small portion of patients, and some individuals experienced disease progression during the neoadjuvant immunotherapy period.[11] In contrast, neoadjuvant immunochemotherapy yielded an extremely higher MPR rate (76.4%) and led to durable survival benefit (2-year OS 94.1%, 2-year EFS 91.2%) with acceptable safety profiles. Additionally, compared with the patients who received standard of care, neoadjuvant immunochemotherapy led to improved OS (2-year OS 94.1% vs. 60%–68%[6,25,26]), indicating the potential value for further clinical expansion.

(C) Heatmap showing the quantitative immunohistochemical results of post-therapy EGFR and downstream proteins in non-responders in arm Cam+TPF compared with arm Cam, including Cam+TPF-pNR group (*n* = 2) and Cam-pNR group (*n* = 7).

(D) Follow-up of the two non-responders in arm Cam+TPF. Red circle indicates primary tumor, red arrow indicates tumor metastasis. EGFR, epidermal growth factor receptor; CT, computed tomography; MRI, magnetic resonance imaging; PET-CT, positron emission tomography and computed tomography.

CellPress
OPEN ACCESS

Prior studies have indicated that combining neoadjuvant immunotherapy with chemotherapy can enhance response rates in HNSCC. For instance, Zhang et al. reported an MPR rate of 74.1% and a pCR rate of 37% among patients with HNSCC, with 93.3% of cases classified as OPSCC and 6.7% as OSCC.[18] Similarly, Wu et al. found an MPR rate of 63% and a pCR rate of 55.6% in HNSCC, with 66.7% of cases as OPSCC and 33.3% as OSCC.[20] The higher pCR rates observed in these studies may be attributed to the higher proportion of OPSCC cases, which are generally more sensitive to chemotherapy.

Our trial revealed an inconsistent objective response rate (ORR) between the primary sites and metastatic LNs. The ORR at primary sites was as high as 47%, whereas it was significantly lower at 2.9% for metastatic LNs. This pattern aligns with findings from other studies, which reported MPR rates of up to 60% in primary tumors compared to only 17% in lymph nodes.[19] Additionally, a separate study on neoadjuvant anti-PD-1 therapy in HNSCC indicated better pathological responses in LNs than at primary sites.[27] The current literature lacks a systematic evaluation of the regression rates in primary tumors versus metastatic LNs in neoadjuvant therapy for HNSCC. Future research should prioritize the assessment of metastatic LNs in the context of neoadjuvant therapies to better understand these discrepancies and optimize treatment strategies.

Previously, the IMCISION study indicated that only patients who achieved MPR to neoadjuvant nivolumab plus ipilimumab experienced improved outcomes in terms of EFS and OS based on limited enrolled patients.[15] In our study, by virtue of the two-arm design, we found that patients with pPR, either in the arm of neoadjuvant immunotherapy or neoadjuvant immunochemotherapy, had a similar 2-year EFS compared with MPR (100% vs. 100%), whereas those with pNR had poor EFS (33.3%). These results suggested that, different from previously reported MPR, pathological response (pCR, MPR, and pPR) to neoadjuvant immunotherapy or neoadjuvant immunochemotherapy was sufficient to confer excellent EFS in patients with OSCC. A recent single-arm study conducted by Wise-Draper revealed that patients with pathological response (tumor regression rate >20%) to neoadjuvant pembrolizumab had a significantly higher DFS in the first year compared to patients who did not respond (93% vs. 72%).[10] But there were still a few patients who developed progressive events after surgery using the aforementioned criteria. Compared with the single-arm study, we identified that pPR (tumor regression rate > 50%) was more sufficient to confer excellent EFS in either neoadjuvant immunotherapy or immunochemotherapy.

Similar to other studies, it is easily understood that therapy resistance could occur in both the neoadjuvant immunotherapy group and the neoadjuvant immunochemotherapy group.[9,10,19] However, our study revealed that non-responders to neoadjuvant immunochemotherapy suffered an extremely higher risk of metastasis and worse outcomes compared to patients who received standard of care. These results emphasize the clinical importance of close monitoring and early intervention of the non-responders who underwent neoadjuvant immunochemotherapy. Due to the limited sample size, the finding should be examined further in other cohorts. This also reminds us to screen the patients who will respond or resist to the therapy at an early stage. According to our signature analysis, the results suggest that patients with a high infiltration level of $CD68^+$ cells and $Foxp3^+$ cells in tumor tissues are likely to benefit from neoadjuvant mono-immunotherapy, and patients with hyperactivated EGFR signaling are potential non-responders to neoadjuvant immunochemotherapy.

In the neoadjuvant camrelizumab plus TPF chemotherapy arm, the most common grade 3–4 adverse events were leukopenia and neutropenia. These are known side effects of TPF chemotherapy[6,7,28] and can be effectively managed through the administration of recombinant human granulocyte stimulating factor. The higher incidence rate of grade 3–4 adverse events did not increase the probability of surgical complications or cause any delays in surgery. Despite a relatively higher incidence rate of SAEs observed in arm neoadjuvant immunochemotherapy, our results still provide evidence that this treatment is considered feasible in patients with locally advanced OSCC. Of note, RCCEP was the most common side effect observed in either two arms. This is thought to be related to the potential pro-angiogenic function of the drug.[29,30]

In summary, neoadjuvant camrelizumab plus TPF chemotherapy led to durable survival benefit with a high pathological response rate and acceptable safety profile in locally advanced OSCC, and pPR appeared as a surrogate primary endpoint. However, non-responders to neoadjuvant immunochemotherapy seemed to have a terrible prognosis and require close monitoring and early intervention, which should be reported timely to benefit the future clinical trials and practices. Also, further evaluation of survival benefit of neoadjuvant immunochemotherapy in randomized, large-scale and multi-center clinical trials is warranted in the future.

Limitations of the study

This trial has several limitations, including an imbalance in N stage between the groups, a small sample size, and a non-comparative two-arm design. First, we acknowledge the imbalance in N stage distribution, which may be attributed to the relatively small number of participants. Notably, the patient cohort in arm Cam+TPF had more advanced N stages, typically associated with poorer outcomes. Second, while our findings suggest that the combination of anti-PD-1 camrelizumab and TPF chemotherapy may offer superior efficacy, definitive conclusions should be drawn from randomized controlled studies, as this study lacks an appropriate control group. It is important to note that neoadjuvant therapy is not currently included in the standard treatment for resectable oral cancer. Considering the current study design and small sample size, we cannot draw definitive conclusions, especially regarding EFS and OS. Ultimately, definitive conclusions regarding neoadjuvant immunotherapy will dependent on well-designed and rigorously controlled randomized clinical trials.

RESOURCE AVAILABILITY

Lead contact

Further information and requests for resources and reagents should be directed to and will be fulfilled by the lead contact, Gang Chen (geraldchan@whu.edu.cn).

Materials availability

This study did not generate new unique reagents.

Data and code availability

- The raw sequence data reported in this paper have been deposited in the Genome Sequence Archive (GSA) at the National Genomics Data Center, China National Center for Bioinformation/Beijing Institute of Genomics, and are publicly accessible at https://ngdc.cncb.ac.cn/gsa-human. Spatial transcriptomic data for patients #41 and #56 are available from GSA (HRA009397). Spatial transcriptomic data for patients #59 and #65 are available from GSA (HRA009391). All other relevant detailed clinical data could be available upon reasonable request from the lead contact.
- No custom computer codes are reported in this paper. The code for data analysis is available upon request.
- Any additional information required to reanalyze the data reported in this work paper is available from the lead contact upon request.

ACKNOWLEDGMENTS

This study was supported by National Natural Science Foundation of China (82341023 and 81922038), School and Hospital of Stomatology, Wuhan University (LYZX202001), the Innovative Research Team of High-level Local Universities in Shanghai (SHSMU-ZLCX20212300), the Fundamental Research Funds for the Central Universities (2042022dx0003 and 2042023kfyq02), and Jiangsu Hengrui Pharmaceuticals. We thank the patients and their families and all members of the collaborative group.

AUTHOR CONTRIBUTIONS

G.C., B.Liu, and J.J. designed the study. G.C. was the principle investigator. H.-M.L., J.-L.Z., W.Z., and B.Li conducted the statistical design and analyses. X.-P.X., Z.-L.Y., Z.S., and J.J. recruited patients. X.-P.X., Z.S., J.J., B.Liu, G.-L.C., H.-Y.G., and Y.-H.Z. treated patients. H.-M.L., X.-P.X., Z.-L.Y., G.-L.C., Z.S., Y.-T.L., X.-X.W., Q.-Y.F., X.-X.C., W.Z., and J.L. collected and analyzed the data. Z.-L.Y., G.-L.C., H.-Y.G., W.Z., and Y.-H.Z. drafted the manuscript. Y.-H.Z., W.Z., and G.C. wrote the manuscript. All authors approved the manuscript.

DECLARATION OF INTERESTS

The authors declare no competing interests.

STAR★METHODS

Detailed methods are provided in the online version of this paper and include the following:

- KEY RESOURCES TABLE
- EXPERIMENTAL MODELS AND STUDY PARTICIPANT DETAILS
  - Human subjects and ethical approval
  - Randomization and masking
  - Sample size justification
- METHOD DETAILS
  - Study design
  - Endpoints and clinical study assessment
  - Tumor histology
  - Spatial transcriptomics data generation
- QUANTIFICATION AND STATISTICAL ANALYSIS
- ADDITIONAL RESOURCES

SUPPLEMENTAL INFORMATION

Supplemental information can be found online at https://doi.org/10.1016/j.xcrm.2025.101930.

Received: July 2, 2024
Revised: October 31, 2024
Accepted: January 3, 2025
Published: January 28, 2025

REFERENCES

1. Gatta, G., Botta, L., Sánchez, M.J., Anderson, L.A., Pierannunzio, D., and Licitra, L.; EUROCARE Working Group: (2015). Prognoses and improvement for head and neck cancers diagnosed in Europe in early 2000s: The EUROCARE-5 population-based study. Eur. J. Cancer *51*, 2130–2143. https://doi.org/10.1016/j.ejca.2015.07.043.
2. Bernier, J., Domenge, C., Ozsahin, M., Matuszewska, K., Lefèbvre, J.-L., Greiner, R.H., Giralt, J., Maingon, P., Rolland, F., Bolla, M., et al. (2004). Postoperative irradiation with or without concomitant chemotherapy for locally advanced head and neck cancer. N. Engl. J. Med. *350*, 1945–1952. https://doi.org/10.1056/NEJMoa032641.
3. Cooper, J.S., Pajak, T.F., Forastiere, A.A., Jacobs, J., Campbell, B.H., Saxman, S.B., Kish, J.A., Kim, H.E., Cmelak, A.J., Rotman, M., et al. (2004). Postoperative concurrent radiotherapy and chemotherapy for high-risk squamous-cell carcinoma of the head and neck. N. Engl. J. Med. *350*, 1937–1944. https://doi.org/10.1056/NEJMoa032646.
4. Zanoni, D.K., Montero, P.H., Migliacci, J.C., Shah, J.P., Wong, R.J., Ganly, I., and Patel, S.G. (2019). Survival outcomes after treatment of cancer of the oral cavity (1985-2015). Oral Oncol. *90*, 115–121. https://doi.org/10.1016/j.oraloncology.2019.02.001.
5. Chow, L.Q.M. (2020). Head and Neck Cancer. N. Engl. J. Med. *382*, 60–72. https://doi.org/10.1056/NEJMra1715715.
6. Zhong, L.-p., Zhang, C.-p., Ren, G.-x., Guo, W., William, W.N., Sun, J., Zhu, H.-g., Tu, W.-y., Li, J., Cai, Y.-l., et al. (2013). Randomized phase III trial of induction chemotherapy with docetaxel, cisplatin, and fluorouracil followed by surgery versus up-front surgery in locally advanced resectable oral squamous cell carcinoma. J. Clin. Oncol. *31*, 744–751. https://doi.org/10.1200/JCO.2012.43.8820.
7. Licitra, L., Grandi, C., Guzzo, M., Mariani, L., Lo Vullo, S., Valvo, F., Quattrone, P., Valagussa, P., Bonadonna, G., Molinari, R., and Cantù, G. (2003). Primary chemotherapy in resectable oral cavity squamous cell cancer: a randomized controlled trial. J. Clin. Oncol. *21*, 327–333. https://doi.org/10.1200/JCO.2003.06.146.
8. Burtness, B., Harrington, K.J., Greil, R., Soulières, D., Tahara, M., de Castro, G., Psyrri, A., Basté, N., Neupane, P., Bratland, Å., et al. (2019). Pembrolizumab alone or with chemotherapy versus cetuximab with chemotherapy for recurrent or metastatic squamous cell carcinoma of the head and neck (KEYNOTE-048): a randomised, open-label, phase 3 study. Lancet *394*, 1915–1928. https://doi.org/10.1016/S0140-6736(19)32591-7.
9. Uppaluri, R., Campbell, K.M., Egloff, A.M., Zolkind, P., Skidmore, Z.L., Nussenbaum, B., Paniello, R.C., Rich, J.T., Jackson, R., Pipkorn, P., et al. (2020). Neoadjuvant and Adjuvant Pembrolizumab in Resectable Locally Advanced, Human Papillomavirus-Unrelated Head and Neck Cancer: A Multicenter, Phase II Trial. Clin. Cancer Res. *26*, 5140–5152. https://doi.org/10.1158/1078-0432.CCR-20-1695.
10. Wise-Draper, T.M., Gulati, S., Palackdharry, S., Hinrichs, B.H., Worden, F.P., Old, M.O., Dunlap, N.E., Kaczmar, J.M., Patil, Y., Riaz, M.K., et al. (2022). Phase II Clinical Trial of Neoadjuvant and Adjuvant Pembrolizumab in Resectable Local-Regionally Advanced Head and Neck Squamous Cell Carcinoma. Clin. Cancer Res. *28*, 1345–1352. https://doi.org/10.1158/1078-0432.CCR-21-3351.
11. Knochelmann, H.M., Horton, J.D., Liu, S., Armeson, K., Kaczmar, J.M., Wyatt, M.M., Richardson, M.S., Lomeli, S.H., Xiong, Y., Graboyes, E.M., et al. (2021). Neoadjuvant presurgical PD-1 inhibition in oral cavity squamous cell carcinoma. Cell Rep. Med. *2*, 100426. https://doi.org/10.1016/j.xcrm.2021.100426.
12. Ferris, R.L., Spanos, W.C., Leidner, R., Gonçalves, A., Martens, U.M., Kyi, C., Sharfman, W., Chung, C.H., Devriese, L.A., Gauthier, H., et al. (2021).

Neoadjuvant nivolumab for patients with resectable HPV-positive and HPV-negative squamous cell carcinomas of the head and neck in the CheckMate 358 trial. J. Immunother. Cancer *9*, e002568. https://doi.org/10.1136/jitc-2021-002568.

13. Schoenfeld, J.D., Hanna, G.J., Jo, V.Y., Rawal, B., Chen, Y.H., Catalano, P.S., Lako, A., Ciantra, Z., Weirather, J.L., Criscitiello, S., et al. (2020). Neoadjuvant Nivolumab or Nivolumab Plus Ipilimumab in Untreated Oral Cavity Squamous Cell Carcinoma: A Phase 2 Open-Label Randomized Clinical Trial. JAMA Oncol. *6*, 1563–1570. https://doi.org/10.1001/jamaoncol.2020.2955.

14. Ferrarotto, R., Bell, D., Rubin, M.L., Hutcheson, K.A., Johnson, J.M., Goepfert, R.P., Phan, J., Elamin, Y.Y., Torman, D.K., Warneke, C.L., et al. (2020). Impact of neoadjuvant durvalumab with or without tremelimumab on CD8+ tumor lymphocyte density, safety, and efficacy in patients with oropharynx cancer. Clin. Cancer Res. *26*, 3211–3219. https://doi.org/10.1158/1078-0432.CCR-19-3977.

15. Vos, J.L., Elbers, J.B.W., Krijgsman, O., Traets, J.J.H., Qiao, X., van der Leun, A.M., Lubeck, Y., Seignette, I.M., Smit, L.A., Willems, S.M., et al. (2021). Neoadjuvant immunotherapy with nivolumab and ipilimumab induces major pathological responses in patients with head and neck squamous cell carcinoma. Nat. Commun. *12*, 7348. https://doi.org/10.1038/s41467-021-26472-9.

16. Hanna, G.J., O'Neill, A., Shin, K.Y., Wong, K., Jo, V.Y., Quinn, C.T., Cutler, J.M., Flynn, M., Lizotte, P.H., Annino, D.J., Jr., et al. (2022). Neoadjuvant and Adjuvant Nivolumab and Lirilumab in Patients with Recurrent, Resectable Squamous Cell Carcinoma of the Head and Neck. Clin. Cancer Res. *28*, 468–478. https://doi.org/10.1158/1078-0432.CCR-21-2635.

17. Ju, W.-T., Xia, R.-H., Zhu, D.-W., Dou, S.-J., Zhu, G.-P., Dong, M.-J., Wang, L.-Z., Sun, Q., Zhao, T.-C., Zhou, Z.-H., et al. (2022). A pilot study of neoadjuvant combination of anti-PD-1 camrelizumab and VEGFR2 inhibitor apatinib for locally advanced resectable oral squamous cell carcinoma. Nat. Commun. *13*, 5378. https://doi.org/10.1038/s41467-022-33080-8.

18. Zhang, Z., Wu, B., Peng, G., Xiao, G., Huang, J., Ding, Q., Yang, C., Xiong, X., Ma, H., Shi, L., et al. (2022). Neoadjuvant Chemoimmunotherapy for the Treatment of Locally Advanced Head and Neck Squamous Cell Carcinoma: A Single-Arm Phase 2 Clinical Trial. Clin. Cancer Res. *28*, 3268–3276. https://doi.org/10.1158/1078-0432.CCR-22-0666.

19. Huang, Y., Sun, J., Li, J., Zhu, D., Dong, M., Dou, S., Tang, Y., Shi, W., Sun, Q., Zhao, T., et al. (2023). Neoadjuvant immunochemotherapy for locally advanced resectable oral squamous cell carcinoma: a prospective single-arm trial (Illuminate Trial). Int. J. Surg. *109*, 2220–2227. https://doi.org/10.1097/JS9.0000000000000489.

20. Wu, D., Li, Y., Xu, P., Fang, Q., Cao, F., Lin, H., Li, Y., Su, Y., Lu, L., Chen, L., et al. (2024). Neoadjuvant chemo-immunotherapy with camrelizumab plus nab-paclitaxel and cisplatin in resectable locally advanced squamous cell carcinoma of the head and neck: a pilot phase II trial. Nat. Commun. *15*, 2177. https://doi.org/10.1038/s41467-024-46444-z.

21. Huang, X., Liu, Q., Zhong, G., Peng, Y., Liu, Y., Liang, L., Hong, H., Feng, W., Yang, S., Zhang, Y., et al. (2022). Neoadjuvant toripalimab combined with gemcitabine and cisplatin in resectable locally advanced head and neck squamous cell carcinoma (NeoTGP01): An open label, single-arm, phase Ib clinical trial. J. Exp. Clin. Cancer Res. *41*, 300. https://doi.org/10.1186/s13046-022-02510-2.

22. Tan, S., Zhang, H., Chai, Y., Song, H., Tong, Z., Wang, Q., Qi, J., Wong, G., Zhu, X., Liu, W.J., et al. (2017). An unexpected N-terminal loop in PD-1 dominates binding by nivolumab. Nat. Commun. *8*, 14369. https://doi.org/10.1038/ncomms14369.

23. Na, Z., Yeo, S.P., Bharath, S.R., Bowler, M.W., Balıkçı, E., Wang, C.-I., and Song, H. (2017). Structural basis for blocking PD-1-mediated immune suppression by therapeutic antibody pembrolizumab. Cell Res. *27*, 147–150. https://doi.org/10.1038/cr.2016.77.

24. Markham, A., and Keam, S.J. (2019). Camrelizumab: First Global Approval. Drugs *79*, 1355–1361. https://doi.org/10.1007/s40265-019-01167-0.

25. Rades, D., Meyners, T., Kazic, N., Bajrovic, A., Rudat, V., and Schild, S.E. (2011). Comparison of radiochemotherapy alone to surgery plus radio(-chemo)therapy for non-metastatic stage III/IV squamous cell carcinoma of the head and neck: A matched-pair analysis. Strahlenther. Onkol. *187*, 541–547. https://doi.org/10.1007/s00066-011-2262-2.

26. Rylands, J., Lowe, D., and Rogers, S.N. (2016). Outcomes by area of residence deprivation in a cohort of oral cancer patients: Survival, health-related quality of life, and place of death. Oral Oncol. *52*, 30–36. https://doi.org/10.1016/j.oraloncology.2015.10.017.

27. Merlino, D.J., Johnson, J.M., Tuluc, M., Gargano, S., Stapp, R., Harshyne, L., Leiby, B.E., Flanders, A., Zinner, R., Axelrod, R., et al. (2020). Discordant Responses Between Primary Head and Neck Tumors and Nodal Metastases Treated With Neoadjuvant Nivolumab: Correlation of Radiographic and Pathologic Treatment Effect. Front. Oncol. *10*, 566315. https://doi.org/10.3389/fonc.2020.566315.

28. Inhestern, J., Schmalenberg, H., Dietz, A., Rotter, N., Maschmeyer, G., Jungehülsing, M., Grosse-Thie, C., Kuhnt, T., Görner, M., Sudhoff, H., et al. (2017). A two-arm multicenter phase II trial of one cycle chemoselection split-dose docetaxel, cisplatin and 5-fluorouracil (TPF) induction chemotherapy before two cycles of split TPF followed by curative surgery combined with postoperative radiotherapy in patients with locally advanced oral and oropharyngeal squamous cell cancer (TISOC-1). Ann. Oncol. *28*, 1917–1922. https://doi.org/10.1093/annonc/mdx202.

29. Cheng, H., Zong, L., Kong, Y., Wang, X., Gu, Y., Cang, W., Zhao, J., Wan, X., Yang, J., and Xiang, Y. (2021). Camrelizumab plus apatinib in patients with high-risk chemorefractory or relapsed gestational trophoblastic neoplasia (CAP 01): a single-arm, open-label, phase 2 trial. Lancet Oncol. *22*, 1609–1617. https://doi.org/10.1016/S1470-2045(21)00460-5.

30. Huang, J., Xu, J., Chen, Y., Zhuang, W., Zhang, Y., Chen, Z., Chen, J., Zhang, H., Niu, Z., Fan, Q., et al. (2020). Camrelizumab versus investigator's choice of chemotherapy as second-line therapy for advanced or metastatic oesophageal squamous cell carcinoma (ESCORT): a multicentre, randomised, open-label, phase 3 study. Lancet Oncol. *21*, 832–842. https://doi.org/10.1016/S1470-2045(20)30110-8.

31. Provencio, M., Nadal, E., Insa, A., García-Campelo, M.R., Casal-Rubio, J., Dómine, M., Majem, M., Rodríguez-Abreu, D., Martínez-Martí, A., De Castro Carpeño, J., et al. (2020). Neoadjuvant chemotherapy and nivolumab in resectable non-small-cell lung cancer (NADIM): an open-label, multicentre, single-arm, phase 2 trial. Lancet Oncol. *21*, 1413–1422. https://doi.org/10.1016/S1470-2045(20)30453-8.

32. Shu, C.A., Gainor, J.F., Awad, M.M., Chiuzan, C., Grigg, C.M., Pabani, A., Garofano, R.F., Stoopler, M.B., Cheng, S.K., White, A., et al. (2020). Neoadjuvant atezolizumab and chemotherapy in patients with resectable non-small-cell lung cancer: an open-label, multicentre, single-arm, phase 2 trial. Lancet Oncol. *21*, 786–795. https://doi.org/10.1016/S1470-2045(20)30140-6.

STAR★METHODS

KEY RESOURCES TABLE

REAGENT or RESOURCE	SOURCE	IDENTIFIER
**Antibodies**		
EGFR	Cell Signaling Technology	Cat #4267; RRID: AB_2246311
p-EGFR	Cell Signaling Technology	Cat #2234; RRID: AB_331701
p-p44/42 MAPK	Cell Signaling Technology	Cat #4370; RRID: AB_2315112
p-p38 MAPK	Cell Signaling Technology	Cat #4511; RRID: AB_2139682
p-Akt	Cell Signaling Technology	Cat #4060; RRID: AB_2315049
CD3ε	Cell Signaling Technology	Cat #85061; RRID: AB_2721019
CD4	Abcam	Cat #ab133616; RRID: AB_2750883
CD8α	Cell Signaling Technology	Cat #85336; RRID: AB_2800052
CD14	Abcam	Cat #ab182032; RRID: AB_3086692
CD68	Cell Signaling Technology	Cat #76437; RRID: AB_2799882
CD11b	Cell Signaling Technology	Cat #49420; RRID: AB_2799357
CD11c	Abcam	Cat #ab52632; RRID: AB_2129793
CD20	Abcam	Cat #ab64088; RRID: AB_1139386
Foxp3	Cell Signaling Technology	Cat #98377; RRID: AB_2747370
CD56	Cell Signaling Technology	Cat #3576; RRID: AB_2149540
TIM-3	Cell Signaling Technology	Cat #45208; RRID: AB_2716862
TIGIT	Abcam	Cat #ab243903; RRID: AB_2943164
PD-1	Cell Signaling Technology	Cat #43248; RRID: AB_2728836
PD-L1	Cell Signaling Technology	Cat #29122; RRID: AB_2798970
**Biological samples**		
Pre-treatment biopsy samples	This study	N/A
Post-treatment resected samples	This study	N/A
**Deposited data**		
Spatial transcriptomic data	This study	GSA: HRA009397, HRA009391
**Software and algorithms**		
GraphPad Prism (Version 8.0)	GraphPad Software, La Jolla California, USA	www.graphpad.com
R (Version 4.1.2)	R Development Core Team, 2008	www.r-project.org
Python (Version 3.9)	Python Software Foundation. Python Language Reference	www.python.org
Aperio digital pathology	Leica, Wetzlar, Germany	www.leicabiosystems.com
**Other**		
Nano Zoomer Digital Pathology scanner	Hamamatsu	Cat #C13239-01

EXPERIMENTAL MODELS AND STUDY PARTICIPANT DETAILS

Human subjects and ethical approval

Eligible patients, both male and female, were aged 18 to 70 years old and had not received prior treatment. They were newly diagnosed histologically proven oral squamous cell carcinoma, clinically staged as III-IVA (T1-2N1-2M0 or T3-4aN0-2M0 according to American Joint Committee on Cancer, AJCC 8th edition). All patients underwent chest CT and whole-body bone scintigraphy, ensuring a comprehensive assessment to exclude the presence of distant metastasis. All eligible patients had ECOG performance status of 0–1 with normal function of major organs and tolerance to the prescribed treatment regimen. The main exclusion criteria were as follows: patients who had experienced immune-related adverse events; those who had previously received antibody or drug targeting T cells or immune checkpoint pathways; and those requiring systemic steroid treatment due to autoimmune diseases. The detailed inclusion and exclusion criteria could be found in supplemented Method S1. After screening, sixty-eight eligible patients were randomly assigned (1:1) to receive camrelizumab (arm Cam) or camrelizumab plus TPF chemotherapy (arm Cam+TPF).

The study was done in accordance with the International Conference on Harmonization Guidelines on Good Clinical Practice and the Declaration of Helsinki. Written informed consent was provided by all study participants. Ethics approval was obtained from the Ethics Committee of the School and Hospital of Stomatology, Wuhan University ([2020] Ethics No.2).

Randomization and masking

Eligible patients were randomly assigned (1:1) to receive camrelizumab (arm Cam) or camrelizumab plus TPF chemotherapy (arm Cam+TPF). The randomization sequence was generated by an independent statistician using PASS 15.0 (NCSS LLC., Kaysville, U.T., USA). The treatment allocation was implemented via opaque, sealed envelopes. Patients, clinicians and study investigators were not masked to treatment assignments.

Sample size justification

The study was an exploratory study with two parallel groups with 1:1 randomization. The MPR rate of the camrelizumab group is expected to be 10%.[9,10] We estimated a sample size of 27 cases would ensure that the precision of 95% confidence interval for the observed MPR is between 2.5% and 24.9%. Considering 20% dropout, the required sample size is 34 cases. According to two relevant single-arm clinical trials published in 2020 that investigated neoadjuvant immunotherapy combined with chemotherapy in non-small cell lung cancer (NSCLC),[31,32] the reported MPR rates for squamous cell carcinoma in these studies were 68.7% and 80%. Given both non-small cell lung cancer (squamous cell carcinoma subtype) and oral squamous cell carcinoma fall under the same histological category, we hypothesized the MPR rate of the camrelizumab combined with TPF chemotherapy group is 70%. The precision of 95% confidence interval of the observed MPR is expected to be between 52.5% and 84.0% if 27 cases were enrolled in the group. Considering 20% dropout, the required sample size is 34 cases. Thus, the required total sample size is 68 cases.

METHOD DETAILS

Study design

This investigator-initiated, single-center, open-label, two-arm, randomized, non-comparative phase II trial was done at the School and Hospital of Stomatology, Wuhan University (NCT04649476). The whole treatment schedule is shown in Figure S1. Enrolled patients were randomized to either arm Cam (neoadjuvant immunotherapy) or arm Cam+TPF (neoadjuvant immunotherapy combined with chemotherapy) to receive indicated treatment. In brief, all patients received 200 mg camrelizumab intravenously on days 1, 15 and 29. For TPF chemotherapy, patients received 75 mg/m^2 docetaxel (T), 75 mg/m^2 cisplatin (P) and 750 mg/m^2 5-fluorouracil (F) intravenously on days 1–5 and days 22–26. All patients received surgery 14 days after the last dose of camrelizumab. The former and final pre-operative imaging was performed 1–3 days before the randomization and surgery, which was used for radiological evaluation. The resection range of surgery was determined based on the pre-treatment tumor bed. Tattoo ink was applied to mark at least four points along the margins of the primary tumors, specifically on the longest and shortest axes. These markings were made 0.5 cm away from the palpable lesion margins. The resection range was determined 1.0 cm away from the markers. The deep margins were primarily determined based on CT imaging. By evaluating the anatomical locations reached by the lesion through CT imaging (for example, oral tongue cancer, whether it involves the lingual septum), the deep surgical margins were guided by the anatomical boundaries during the surgery. Intraoperative rapid frozen section biopsy of the tumor margins was performed to ensure complete resection of the tumor. Patients received postoperative risk-adaptive adjuvant therapy within 4–6 weeks after surgery. Patients with high-risk pathology features such as positive tumor margin (less than 5 mm or tumor cells observed on the margin), extracapsular lymph node metastasis, facial invasion (cheek) were supplemented with platinum-based chemotherapy concurrently with radiotherapy. All adverse events experienced by patients were collected from the first study intervention, throughout the whole study process, and at least 90 days after the last treatment.

Endpoints and clinical study assessment

The primary endpoint was MPR rate. Secondary endpoints were radiological response, 2-year event-free survival (EFS) rate, 2-year OS rate and treatment-related adverse events (TRAEs). The evaluation process of pathological response rate was shown in Figure S11. The entire tumor bed and all sampled lymph nodes were examined histologically in patients who had pathological complete response (pCR), which was defined as the absence of viable tumor in all slides. MPR was defined as the presence of 10% or less viable residual tumor in the resected tumor specimens. Pathological partial response (pPR) was defined as presence of more than 10% and less than 50% viable residual tumor and pathological non-response (pNR) was defined as presence of more than 50% viable residual tumor in the resected tumor specimens. The radiological response was evaluated by radiographic examinations and defined by Response Evaluation Criteria in Solid Tumors (RECIST) version 1.1. Adverse events were assessed according to the National Cancer Institute Common Terminology Criteria for Adverse Events (CTCAE) version 5.0 and Clavien-Dindo classification of surgical complications. Event-free survival was defined as the time from randomization to the first occurrence of disease progression or recurrence, or death from any cause. Disease-free survival was defined as the time from surgery to disease recurrence or death from any cause.

Tumor histology

To detect the expression of programmed cell death-ligand 1 (PD-L1), immunohistochemical staining was carried out using anti-PD-L1 (CST, 405.9A11, 29122S, 1:200). Combined positive score (CPS) was defined as the total number of PD-L1-stained cells (including tumor cells, tumor-associated lymphocytes, and macrophages) divided by the total number of viable tumor cells, multiplied by 100.

Tumor biopsy and surgical samples were collected and examined for a panel of checkpoint molecules, immunocyte subsets and epidermal growth factor receptor (EGFR) signaling proteins using immunohistochemistry, including EGFR (CST, D38B1, 4267S, 1:400), *p*-EGFR (Tyr1068) (CST, 2234S, 1:200), *p*-p44/42 MAPK (Erk1/2) (Thr202/Tyr204) (CST, D13.14.4E, 4370S, 1:200), *p*-p38 MAPK (Thr180/Tyr182) (CST, D3F9, 4511S, 1:200), *p*-Akt (Ser473) (CST, D9E, 4060S, 1:200), CD3ε (CST, D7A6E, 85061S, 1:200), CD4 (Abcam, ab133616, 1:400), CD8α (CST, D8A8Y, 85336S, 1:400), CD14 (Abcam, ab182032, 1:400), CD68 (CST, D4B9C, 76437S, 1:400), CD11b (CST, D6X1N, 49420S, 1:200), CD11c (Abcam, ab52632, 1:200), CD20 (Abcam, ab64088, 1:200), Foxp3 (CST, D2W8E, 98377S, 1:100), CD56 (CST, 123C3, 3576S, 1:100), TIM-3 (CST, D5D5R, 45208S, 1:50), TIGIT (Abcam, ab243903, 1:200), PD-1 (CST, EH33, 43248S, 1:100). Slides were scanned at ×20 magnification on Nano Zoomer Digital Pathology scanner (Hamamatsu) and analyzed using Aperio Digital Pathology software (Leica).

Spatial transcriptomics data generation

We conducted spatial transcriptomics using Visium technology from 10x Genomics on tissue samples from patients #41, #56, #59, and #65. RNA was carefully extracted from 10 μm sections of formalin-fixed paraffin-embedded (FFPE) tissue using the Qiagen RNeasy FFPE Kit. The quality of the extracted RNA was evaluated using the Agilent RNA 6000 Pico Kit, selecting blocks with a DV200 value above 50% for further analysis. Each tissue section was precisely positioned within the capture area of a Visium Spatial Gene Expression Slide (PN-1000188, 10x Genomics). The tissue sections underwent deparaffinization, staining, and decrosslinking, followed by hybridization, ligation, release, and extension processes. Spatial gene expression libraries from FFPE samples were constructed using the Visium Human Transcriptome Probe Kit (PN-1000363) and the Visium FFPE Reagent Kit (PN-1000363), following the prescribed protocols. These libraries were sequenced using the Illumina NovaSeq 6000 systems, targeting a minimum of 75,000 read pairs per spot and the detection of approximately 2,000 genes per spot. Subsequent analysis was carried out using the Scanpy package in python.

QUANTIFICATION AND STATISTICAL ANALYSIS

All the statistical analyses were conducted using GraphPad Prism v.8.0 (GraphPad Software, Inc., San Diego, CA, USA) and R version 4.1.2 (R Foundation for Statistical Computing, Vienna, Austria). Statistical parameters were reported in the figure legend. Continuous variables were expressed as median (range) and compared between the two groups using the Student's t test or Mann-Whitney test. Categorical variables were expressed as frequency (percentage) and compared between the two groups using chi-square test or Fisher exact test. Survival was estimated using the Kaplan-Meier method and p value was calculated through log-rank test. All reported p-value were 2-tailed, and the statistical significance was defined as $p < 0.05$.

ADDITIONAL RESOURCES

This clinical trial has been registered on https://clinicaltrials.gov (NCT04649476).

Cell Reports Medicine, Volume *6*

Supplemental information

Neoadjuvant immunotherapy with or without chemotherapy in locally advanced oral squamous cell carcinoma: Randomized, two-arm, phase 2 trial

Hai-Ming Liu, Xue-Peng Xiong, Zi-Li Yu, Zhe Shao, Gai-Li Chen, Yu-Tong Liu, Xin-Xin Wang, Qiu-Yun Fu, Xiao-Xia Cheng, Jing Li, Jia-Li Zhang, Bo Li, Hong-Yun Gong, Ya-Hua Zhong, Wei Zhang, Jun Jia, Bing Liu, and Gang Chen

Document S1. Figures S1-S11.

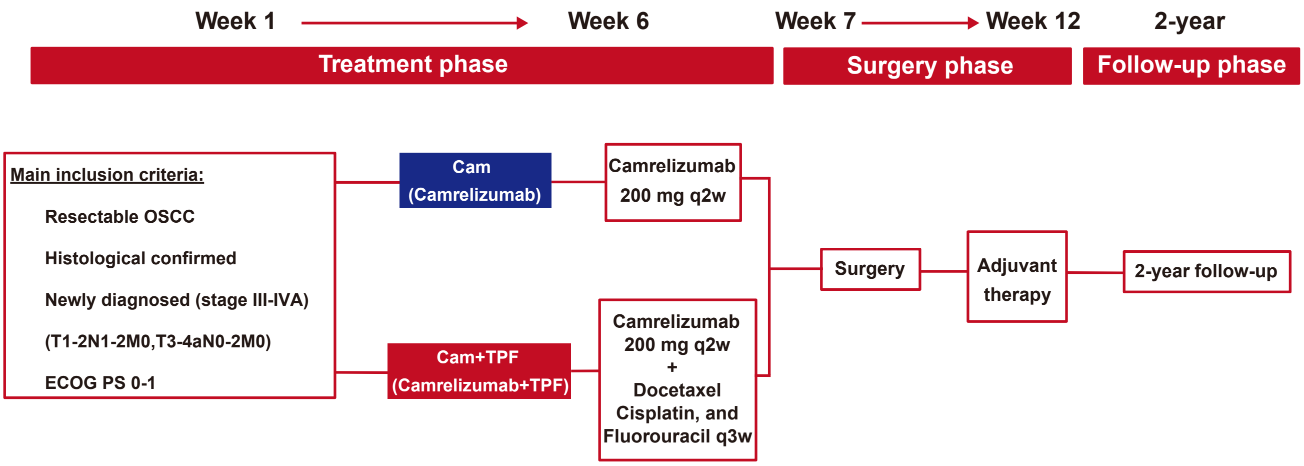

Figure S1. Therapeutic schedule. Related to Figure 1 and STAR methods.

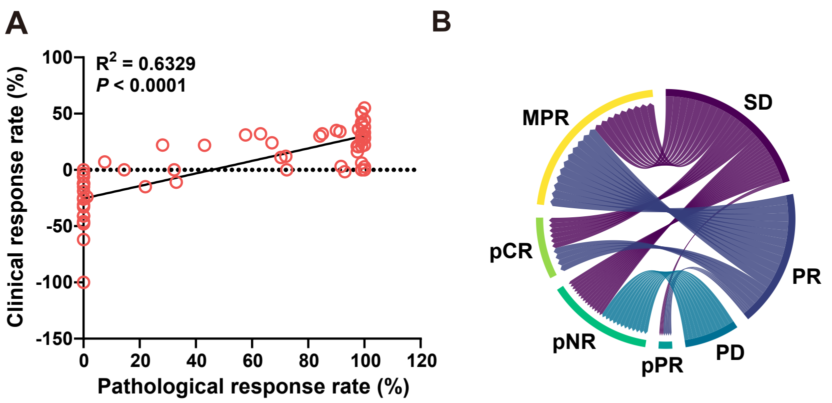

Figure S2. Correlation between radiological response and pathological response. Related to Figure 2.
(A) Correlation between radiological response rate and pathological response rate. Correlation was analyzed by Pearson's correlation.
(B) Correlation between ORR per RECIST and pathological response. ORR, objective response rate;
RECIST, response evaluation criteria in solid tumors.

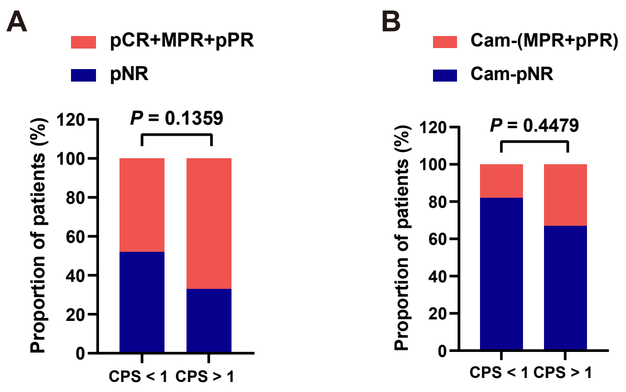

Figure S3. Correlation between CPS of PD-L1 and pathological response. Related to Figure 2.
(A) Correlation between CPS of PD-L1 and pathological response in arm Cam and arm Cam+TPF. *P* value was determined by two-sided Fisher's exact test.
(B) Correlation between CPS of PD-L1 and pathological response in arm Cam. CPS, combined positive score.

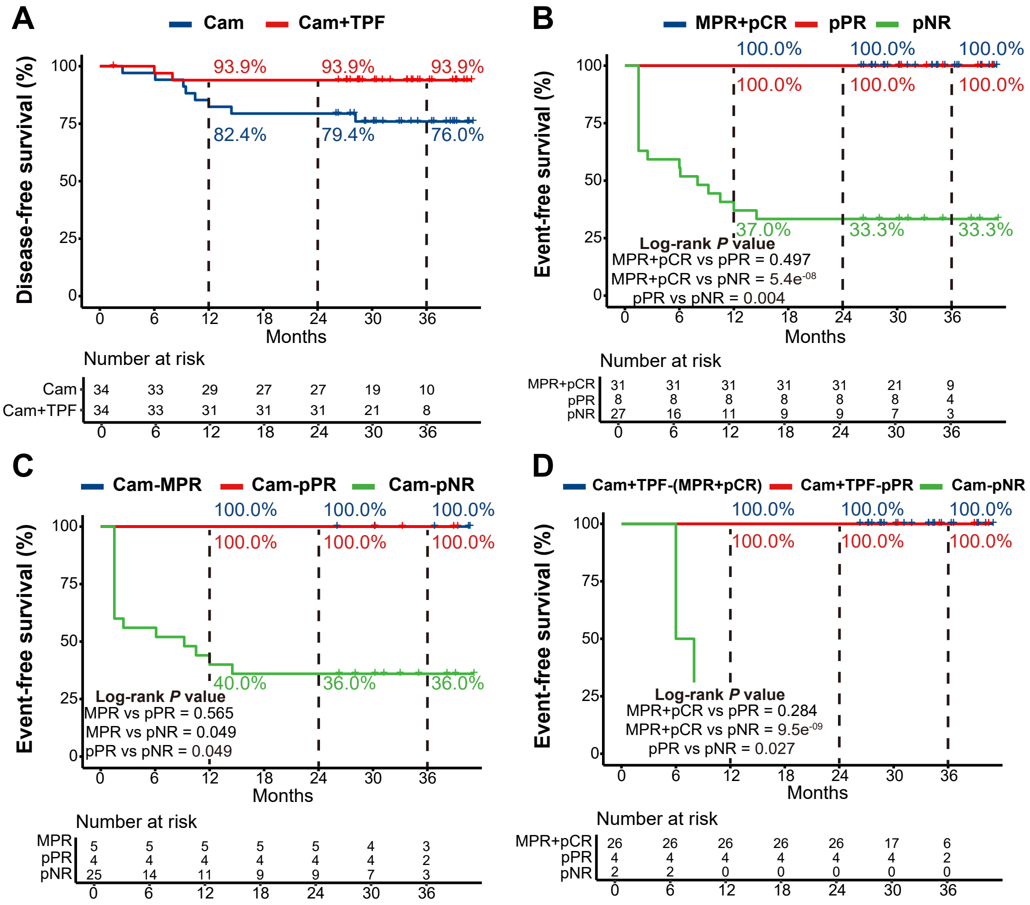

Figure S4. Survival outcomes of patients. Related to Figure 3.
(A) Kaplan-Meier curve of disease-free survival for the patients in arm Cam and arm Cam+TPF.
(B) Kaplan-Meier curve of event-free survival for the patients grouped by different pathological response.
(C) Kaplan-Meier curve of event-free survival for the patients in arm Cam grouped by different pathological response.
(D) Kaplan-Meier curve of event-free survival for the patients in arm Cam+TPF grouped by different pathological response.
pCR, pathological complete response; MPR, major pathological response; pPR, pathological partial response; pNR, pathological non-response. Log rank *P* value was used to estimate the significance among groups.

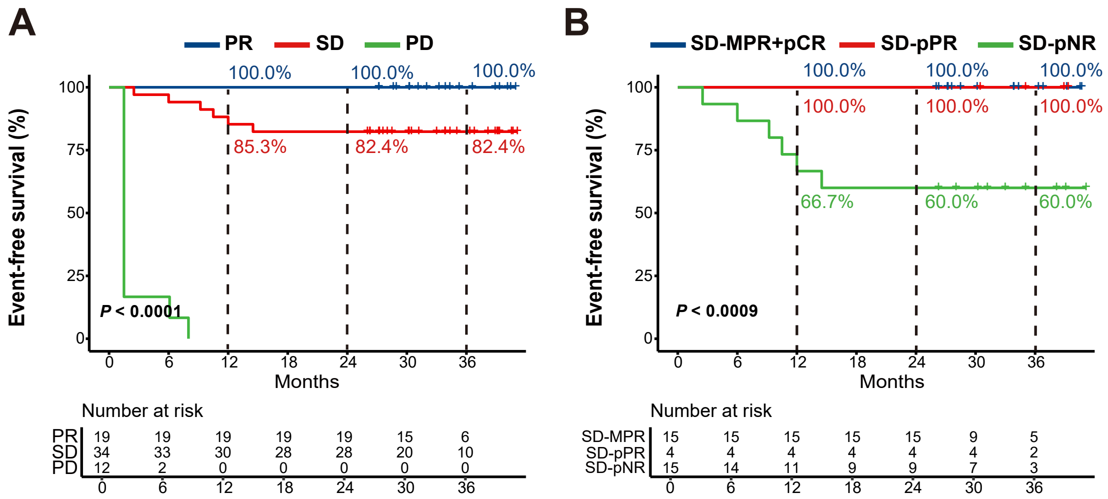

Figure S5. Survival outcomes of patients. Related to Figure 3.

(A) Kaplan-Meier curve of event-free survival for the patients grouped by RECIST criteria.

(B) Kaplan-Meier curve of event-free survival for the patients achieved stable disease grouped by pathological response.

PR, partial response; SD, stable disease; PD, progressive disease; pCR, pathological complete response;

MPR, major pathological response; pPR, pathological partial response; pNR, pathological non-response.

Log rank *P* value was used to estimate the significance among groups.

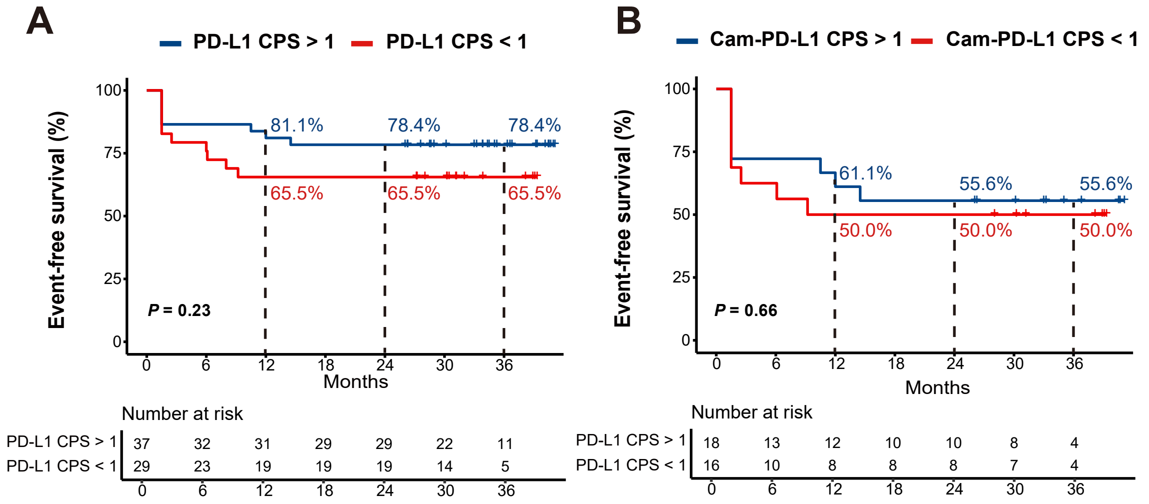

Figure S6. Survival outcomes of patients. Related to Figure 3.

(A) Kaplan-Meier curve of event-free survival for the patients grouped by CPS of PD-L1 in arm Cam and arm Cam+TPF.

(B) Kaplan-Meier curve of event-free survival for the patients grouped by CPS of PD-L1 in arm Cam.

CPS, combined positive score. Log rank *P* value was used to estimate the significance among groups.

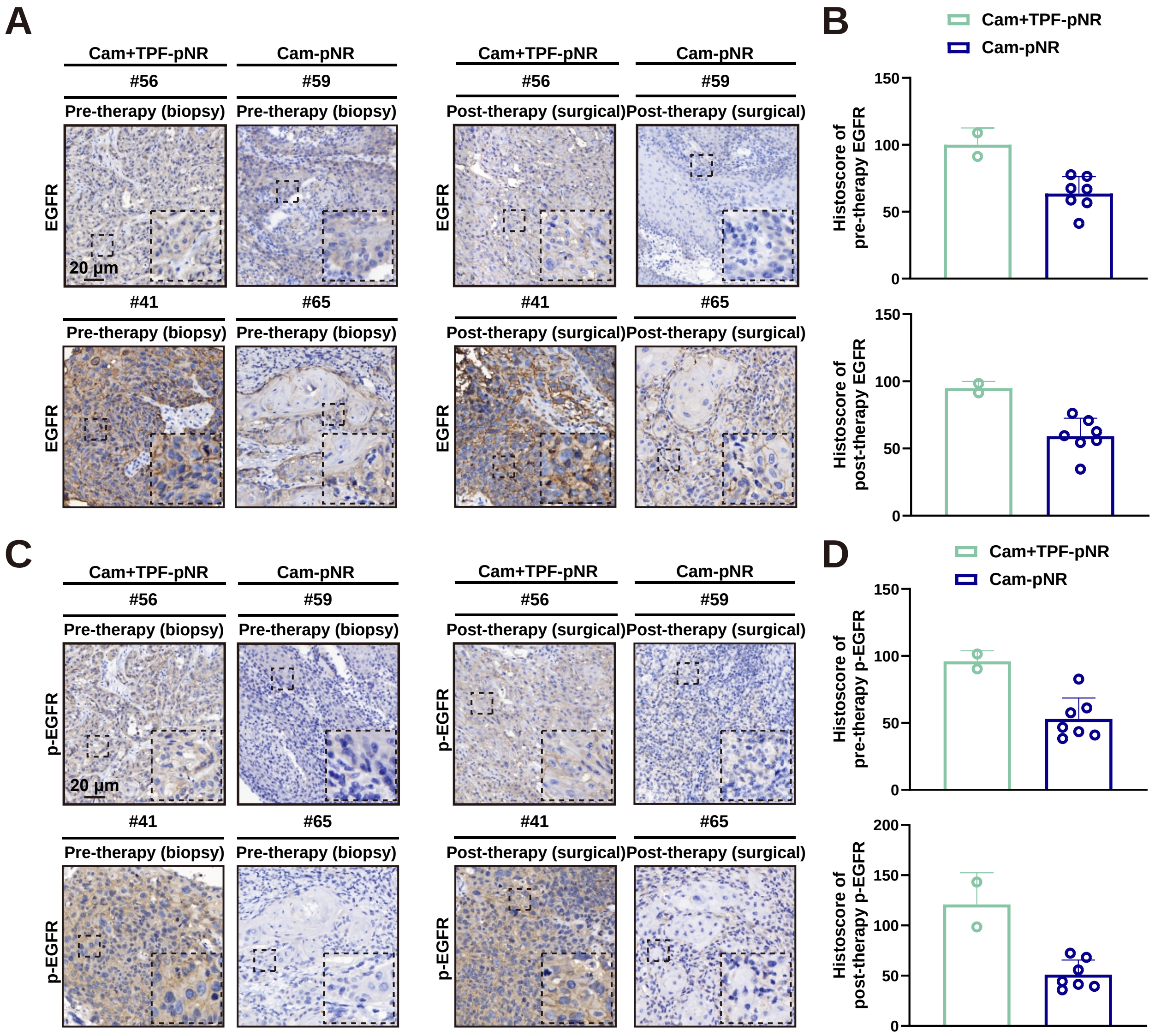

Figure S7. Immunohistochemical staining of EGFR and p-EGFR of pathological non-responders in arm Cam and Cam+TPF. Related to Figure 4.

(A) Immunohistochemical staining of pre-therapy and post-therapy EGFR of pathological non-responders in arm Cam and Cam+TPF, including Cam+TPF-pNR group (n = 2) and Cam-pNR group (n = 2), scale bar, 20 µm.

(B) Histogram of quantification of pre-therapy and post-therapy EGFR expression of pathological non-responders in arm Cam and Cam+TPF, including Cam+TPF-pNR group (n = 2) and Cam-pNR group (n = 7), data are represented as mean ± SD.

(C) Immunohistochemical staining of pre-therapy and post-therapy p-EGFR of pathological non-responders in arm Cam and Cam+TPF, including Cam+TPF-pNR group (n = 2) and Cam-pNR group (n = 2), scale bar, 20 µm.

(D) Histogram of quantification of pre-therapy and post-therapy p-EGFR expression of pathological non-responders in arm Cam and Cam+TPF, including Cam+TPF-pNR group (n = 2) and Cam-pNR group (n = 7), data are represented as mean ± SD.

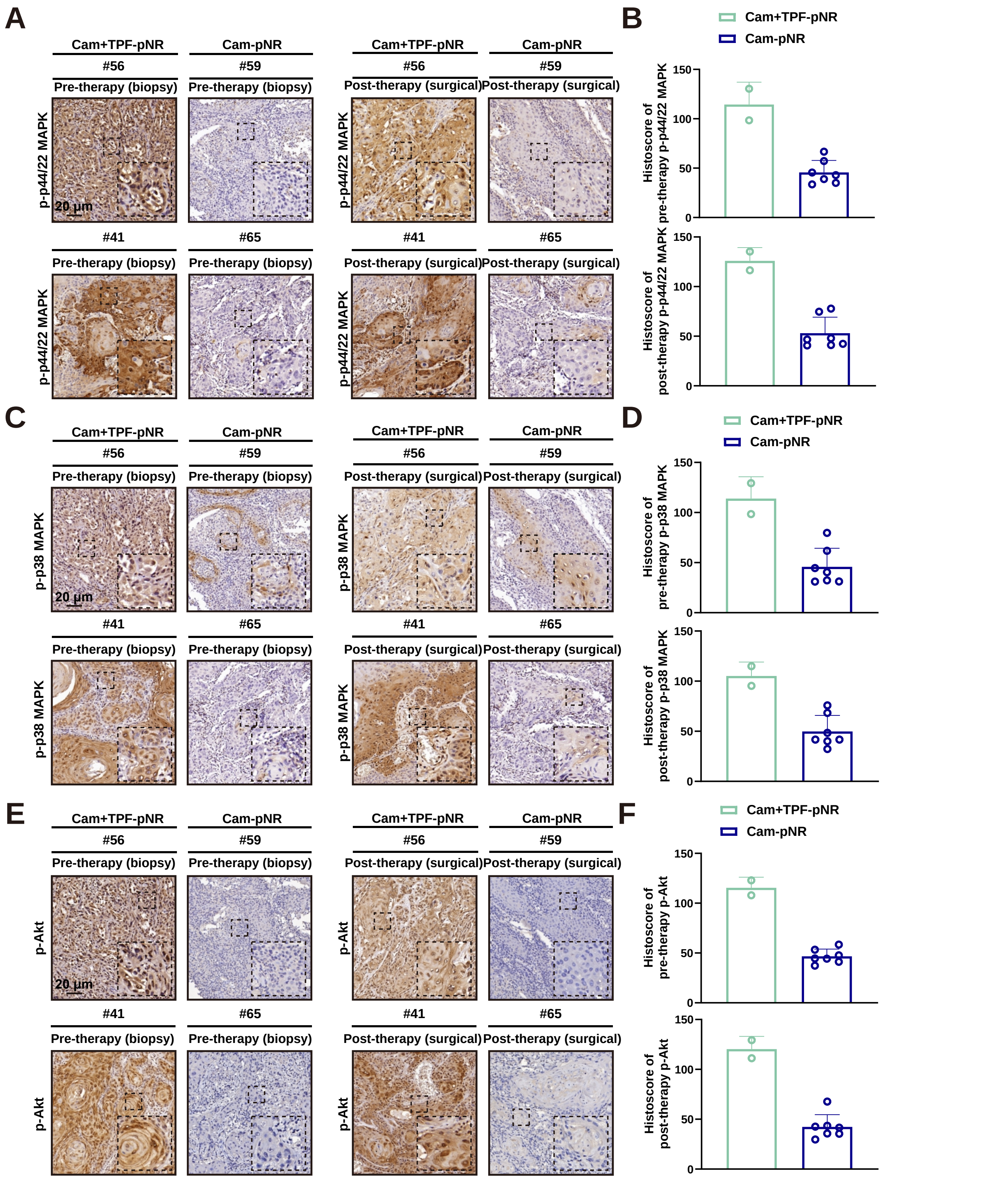

Figure S8. Immunohistochemical staining of EGFR downstream proteins of pathological non-responders in arm Cam and Cam+TPF. Related to Figure 4.

(A) Immunohistochemical staining of pre-therapy and post-therapy p-p44/22 MAPK of pathological non-responders in arm Cam and Cam+TPF, including Cam+TPF-pNR group (n = 2) and Cam-pNR group (n = 2), scale bar, 20 μm.

(B) Histogram of quantification of pre-therapy and post-therapy p-p44/22 MAPK expression of pathological non-responders in arm Cam and Cam+TPF, including Cam+TPF-pNR group (n = 2) and Cam-pNR group (n = 7), data are represented as mean ± SD.

(C) Immunohistochemical staining of pre-therapy and post-therapy p-p38 MAPK of pathological non-responders in arm Cam and Cam+TPF, including Cam+TPF-pNR group (n = 2) and Cam-pNR group (n = 2), scale bar, 20 μm.

(D) Histogram of quantification of pre-therapy and post-therapy p-p38 MAPK expression of pathological non-responders in arm Cam and Cam+TPF, including Cam+TPF-pNR group (n = 2) and Cam-pNR group (n = 7), data are represented as mean ± SD.

(E) Immunohistochemical staining of pre-therapy and post-therapy p-Akt of pathological non-responders in arm Cam and Cam+TPF, including Cam+TPF-pNR group (n = 2) and Cam-pNR group (n = 2), scale bar, 20 μm.

(F) Histogram of quantification of pre-therapy and post-therapy p-Akt expression of pathological non-responders in arm Cam and Cam+TPF, including Cam+TPF-pNR group (n = 2) and Cam-pNR group (n = 7), data are represented as mean ± SD.

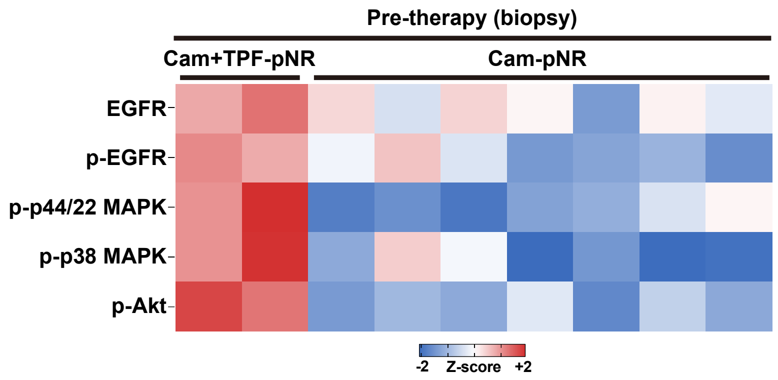

Figure S9. Heatmap showing quantitative immunohistochemical staining of pre-therapy EGFR downstream proteins of pathological non-responders in arm Cam+TPF compared with arm Cam, including Cam+TPF-pNR group (n = 2) and Cam-pNR group (n = 7). Related to Figure 4.

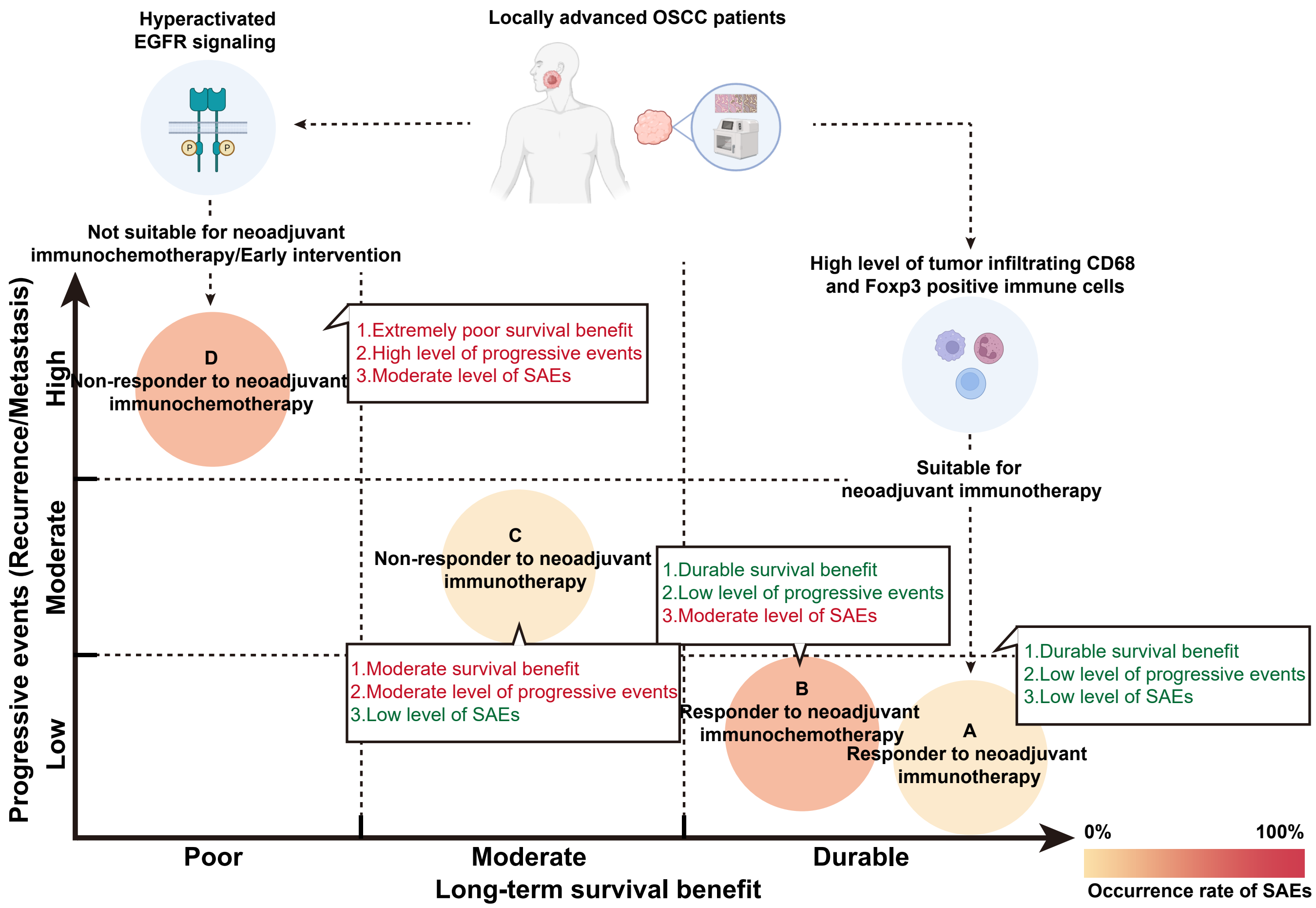

Figure S10. Decision making strategy for patients received neoadjuvant immunotherapy or immunochemotherapy. Related to Figure 3 and Figure 4.

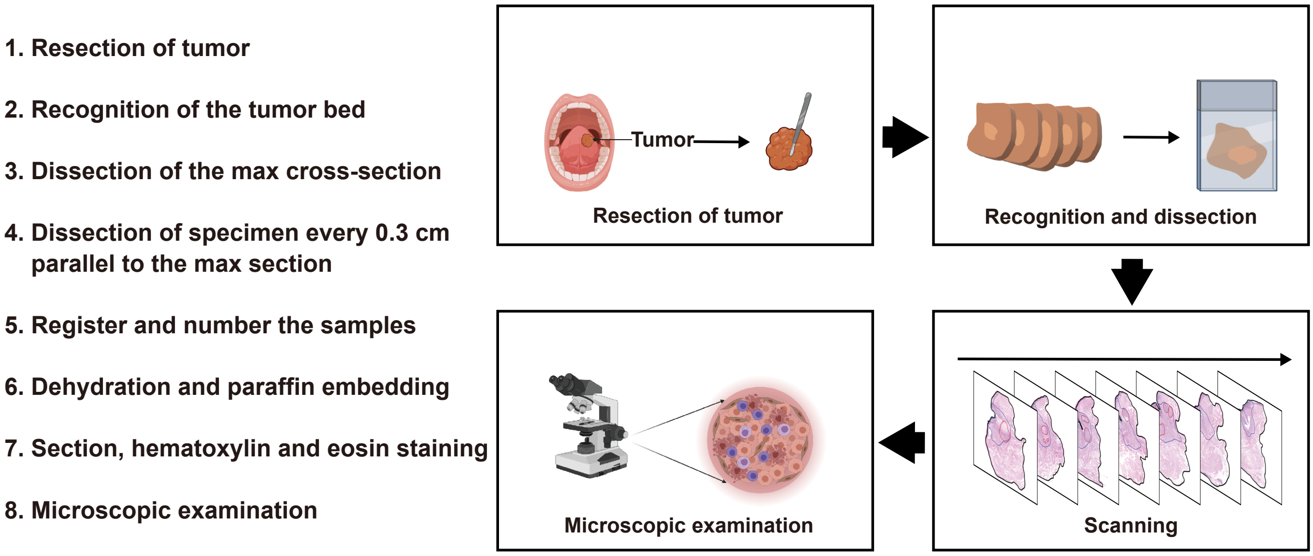

Figure S11. Work flow of pathological evaluation. Related to STAR methods.

Method S1. Study protocol. Related to STAR methods.

Clinical trial protocol information

Study Title: Neoadjuvant PD-1 Blockade in Resectable Oral Squamous Cell Carcinoma

Official Title: A Randomized Phase II Study of Neoadjuvant PD-1 Blockade Alone or Plus TPF Induction Chemotherapy for Resectable Local Advanced Oral Squamous Cell Carcinoma

NCT registration number: **NCT04649476**

Center: **Hospital of Stomatology, Wuhan University**

Supplied agents: **Camrelizumab, Jiangsu HengRui Medicine Co., Ltd**

Principal investigator: **Gang Chen, Professor**

Date: **3/25/2022**

Protocol version: **1.2**

Contents

Study title: Neoadjuvant PD-1 blockade in resectable oral squamouscell carcinoma
Contents
1. Background and rational
    1.1. Background
    1.2. Cancer immunotherapy
    1.3. Expressions of PD-L1 in human
    1.4. Research of recurrent or metastatic head and neck squamous cell carcinoma
    1.5. Treatment of local advanced OSCC
    1.6. Rational for preoperative neoadjuvant immunotherapy in OSCC
    1.7. Clinical trials of immunotherapy in OSCC
    1.8. Development of Camrelizumab
    1.9. Clinical experience with Camrelizumab
2. Purpose
3. Objective
    3.1. Primary endpoint
    3.2. Secondary endpoint
    3.3. Exploratory endpoint
4. Study design and treatment plan
    4.1. Recruitment
    4.2. Determination of Eligibility
    4.3. Study design and toxicity assessments
    4.4. Diagnostic and surgical evaluation
    4.5. Adjuvant therapy
    4.6. Perioperative safety assessment
5. Laboratory research
    5.1. Tissue specimen
    5.2. Handling of biopsy specimen
    5.3. Handling of surgical tumor specimen
    5.4. Handling of blood
    5.5. Handling of saliva
6. Screening and discontinued /withdrawal
    6.1. Inclusion criteria
    6.2. Exclusion Criteria
    6.3. Withdrawal from study
7. Study procedures
    7.1. Clinical operation schedule
    7.2. Specimen processing schedule
8. Study drug
    8.1. Drug description
    8.2. Pharmacokinetics of camrelizumab
    8.3. Safety summary and side effects of camrelizumab
    8.4. Disposal and distribution of camrelizumab

8.5. Injection method of camrelizumab
8.6. Destruction of research drugs
9. Safety evaluation
9.1. Safety summary
9.2. Adverse event
9.3. Sorting and reporting of serious adverse events
9.4. Non-serious adverse events
9.5. Abnormal laboratory test results
9.6. Gestation
9.7. Overuse
9.8. Other safety precautions
10. Effectiveness evaluation
11. Exploratory study
11.1. Tissue and body fluid analysis
11.2. Screening of immune response markers for anti-PD-1 therapy
11.3. Indicators to be detected
12. Management of adverse events
12.1. Infusion reaction
12.2. Immune-related adverse events
13. Research Management
13.1. Amendments to the Scheme
13.2. Informed Consent
13.3. Code of Ethics and Practice for the Management of Drug Clinical Trials
13.4. Regulatory authorities
13.5. Principal Investigator (PI) Responsibilities
14. Data Management and Security Controls
14.1. Data collection
14.2. Data curation
14.3. Discussion Meetings
14.4. Quality Control
15. Statistical analysis plan
15.1. Study design
15.2. Data Analysis Plan
16. Prospective research plan
17. Informed consent
References

1. Background and rational

1.1Background

Oral squamous cell carcinoma (OSCC) is the most common malignant tumor in the oral and maxillofacial region, with a high incidence and a poor prognosis. The 5-year survival rate of patients with OSCC is about 50%. Among them, local advanced patients have a lower 5-year survival rate, only about 30%, due to high tumor burden, recurrence and metastasis. Currently, radical surgery combined with postoperative radiotherapy or chemoradiotherapy is the main treatment for local advanced OSCC, but its effect is not ideal. In recent years, researchers have tried the treatment of TPF induction chemotherapy (Docetaxel-cisplatin-5-fluorouracil) for patients with local advanced OSCC, but these results failed to increase the 5-year overall survival rate. Therefore, how to improve the treatment effect for patients with local advanced OSCC is a major challenge currently faced by oral and maxillofacial-head and neck tumor surgeons.

In recent years, the rapid rise of immunotherapy that won the 2018 Nobel Prize has brought hope to patients with advanced cancer. Among them, PD-L1/PD-1 blockade is currently a representative drug for tumor immunotherapy. It has been proven to have a significant and long-lasting effect in the treatment of patients with recurrent or metastatic malignant melanoma, lung cancer and head and neck squamous cell carcinoma who have lost the opportunity for surgery. What's more, the results of two clinical trials recently completed also suggest that the effect is much surprising if PD-1 monoclonal antibody is used as neoadjuvant immunotherapy for operable malignant tumors. Forde et al. performed two cycles of PD-1 monoclonal antibody neoadjuvant immunotherapy on patients with operable non-small cell lung cancer before surgery, and found that 45% (9/20) of the patients had pathological remission, and 3 of them had complete Pathological remission, and only 15% of patients have recurrence or metastasis 1 year after surgery, and the overall survival rate is as high as 90%. At the same time, Huang et al. treated patients with stage III/IV operable malignant melanoma with one PD-1 monoclonal antibody treatment before surgery, which resulted in 30% (8/27) patients with complete or overall pathological remission. The annual progression-free survival rate was 63%, and the overall two-year survival rate reached 93%. Taken together, PD-1 blockade is very promising as a neoadjuvant treatment to improve the efficacy of operable local advanced OSCC patients.

Of note, immunotherapy is not effective for every patient, less than 30% of patients can benefit from PD-1 blockade, whether it is used for the treatment of inoperable recurrent or metastatic tumors or for the preoperative adjuvant treatment of operable tumors. Therefore, further elucidating the precise regulation mechanism of tumor immune escape, exploring the molecular mechanism of immunotherapy tolerance, and finding biomarkers that can predict the efficacy in an early stage are important for achieving precise tumor immunotherapy and effectively prolonging the survival time and improving the quality of life of patients with malignant tumors. Our research has found that tumor cells can exert immunosuppressive effects by secreting PD-L1^{+} extracellular vesicles. More importantly, the concentration level of PD-L1^{+} extracellular vesicles in the peripheral blood of patients with metastatic malignant melanoma and its change trend and amplitude in the early stage of immunotherapy can effectively evaluate the patient’s treatment effect. Based on the above research, we propose a liquid biopsy strategy based on the level of circulating PD-L1^{+} extracellular vesicles in the peripheral blood of tumor patients to predict the effectiveness of immunotherapy, which provides a fast and accurate method for early prediction of the efficacy of tumor immunotherapy. It has obvious advantages when compared with traditional tissue biopsy. At present, the research results have applied for international invention patents. However, whether this liquid biopsy strategy based on extracellular vesicles can be used to predict the efficacy of neoadjuvant immunotherapy, especially when used in the treatment of patients with local advanced OSCC, remains to be studied.

1.2 Cancer immunotherapy

Cancer immunotherapy is to restore the body's normal tumor immune response by reinvigorating the tumor-immune cycle, so as to achieve the purpose of eliminating tumors. Cancer immunotherapy mainly include monoclonal antibody immune checkpoint inhibitors (CTLA-4 inhibitors, PD-1/PD-L1 inhibitors, etc.), therapeutic antibodies (anti-CD20 monoclonal antibodies, anti-CD30 monoclonal antibodies, anti-Her2 monoclonal antibodies, etc.), cancer vaccines, cell therapy (CAR-T therapy, etc.) and small molecule inhibitors, etc. Among them, PD-1/PD-L1 inhibitors are currently the most studied and fastest-growing immunotherapy. Immune checkpoint inhibitors. Since the US FDA approved the first PD-1 inhibitor Keytruda (Pembrolizumab) for the treatment of malignant melanoma in 2014, there have been many PD-1/PD-L1 inhibitors in the

market. The indications involve non-small cell lung cancer, melanoma, renal cell carcinoma, urothelial carcinoma, Hodgkin's lymphoma, hepatocellular carcinoma, advanced head and neck squamous cell carcinoma, etc. At present, the FDA has approved Pembrolizumab, Nivolumab, Atezolizumab, Duralumab, Avelumab, etc. for clinical treatment, and domestic PD-1/PD-L1 inhibitors such as Camrelizumab, Sintilimab, Toripalimab, etc.

In the current clinical trials, PD-1/PD-L1 inhibitors show obvious effects in patients with advanced unresectable malignant tumors, and many studies have confirmed that they are used as neoadjuvant immunotherapy in the local advanced stages.

1.3 Expressions of PD-L1 in human

Constitutive PD-L1 expression is normally limited to macrophage-lineage cells, although expression of PD-L1 can be is generally expressed in cells of the macrophage cell line. When malignant tumors appear, PD-L1 is expressed in most malignant tumor cells. The expression of PD-L1 in patients with OSCC is related to the depth of tumor invasion, which further suggests that the expression of PD-L1 is related to tumor invasion and metastasis. Although there is still some controversy as to whether the expression of PD-L1 can evaluate the prognosis of OSCC, it has been confirmed that PD-L1 is related to poor prognosis in other tumor models.

1.4 Research of recurrent or metastatic head and neck squamous cellcarcinoma

Nivolumab and Pembrolizumab have been approved for the treatment of recurrent or metastatic head and neck squamous cell carcinoma (R/MHNSCC). The clinical trial of Pembrolizumab (NCT01848834) confirmed that the overall response rate of imaging examination was 18%, and the patients could well tolerate the side effects. In another phase 3 clinical trial of R/MHNSCC (NCT02105636), the overall survival of patients treated with Nivolumab was longer than those with standard of care (7.5 months versus 5.5 months), and the one-year survival rate was also significantly improved (36.0% versus 16.6%)), the incidence of serious side effects of grade 3 to 4 is relatively low (13.1% versus 35.1%).

1.5 Treatment of local advanced OSCC

At present, surgical resection combined with postoperative adjuvant treatment is still the main treatment for local advanced OSCC. Though surgical skills and radiotherapy and chemotherapy techniques have made great progress in the past 2 decades, the five-year survival rate of patients

with OSCC remains between 50% and 60%. In view of the high recurrence rate and metastasis rate, TPF inductive chemotherapy had been applied for local advanced OSCC, but the results show that it cannot effectively improve the survival rate of patients. The NCCN guidelines point out that for patients with local advanced OSCC, in addition to conventional treatment, it is recommended to conduct clinical trials to explore better treatment options.

1.6 Rational for preoperative neoadjuvant immunotherapy in OSCC

The main purpose of preoperative neoadjuvant immunotherapy for OSCC is to eliminate residual imaging and invisible lesions after surgical resection, and to prevent recurrence or metastasis caused by deep infiltration or scattered micro-metastasis. However, after a major surgical trauma, the patient's general condition is more difficult to tolerate the side effects of adjuvant therapy. On the other hand, it is difficult for chemotherapy drugs to enter the tumor microenvironment through the blood supply. Neoadjuvant immunotherapy can solve these problems. What’s more, there are literature reports confirming that PD-1/PD- L1 inhibitors can reduce the pathological stage of tumors and bring better prognostic effects to patients.

1.7 Clinical trials of immunotherapy in OSCC

Oral squamous cell carcinoma has been shown to be an immunosuppressive disease. Studies have found that there is a decrease in infiltrating lymphocytes in oral squamous cell carcinoma, while inhibitory regulatory T-cell proliferation promotes tumor immune escape. Disruption of the function and antigen presentation capacity of natural killer cells is a manifestation of impaired immune cell function. The expression of PD-L1 in tumor tissues has a close relationship with the depth of tumor invasion and lymph node metastasis, and the depth of tumor infiltration and lymph node metastasis are closely related to the patient's poor prognosis.

Ferris et al, studied PD-1 inhibitors for recurrent head and neck squamous cell carcinoma. The result showed that PD-1 inhibitors achieved satisfactory results compared with cisplatin chemotherapy. PD-1 inhibitors significantly improved overall survival and one-year survival, and the incidence of serious adverse events was also found to be lower in patients treated with PD-1 inhibitors than in patients treated with cisplatin chemotherapy.

1.8 Development of Camrelizumab

Camrelizumab is a high-affinity IgG4 (kappa) anti-programmed cell death 1 (PD-1)

monoclonal antibody. By competing with PD-L1 or PD-L2 to bind PD-1 molecules to block the PD-1/PD-L1 axis signaling pathway to increase tumor antigen-specific T cells and restart the tumor-immune cycle. After a single injection of different doses of Camrelizumab, the T cell receptor binding rates of subjects at 60, 200, and 400 mg doses were 81%, 85%, and 88%, respectively.

Since the first phase I clinical trial in Australia in 2015, the research of Camrelizumab on Hodgkin's lymphoma, B cell lymphoma, esophageal squamous cell carcinoma, gastric-esophageal junction cancer, liver cancer has been launched in the Cancer Hospital of the Chinese Academy of Medical Sciences, the Cancer Center of Sun Yat-sen University, and Peking University Cancer Hospital.

1.9 Clinical experience with Camrelizumab

The results of 9 clinical trials published by Jiangsu HengRui Medicine Co., Ltd show that the immune-related adverse event (irAE) rate is 97%, most of the irAEs are mild to moderate including skin reactions, fever, hypothyroidism, fatigue, cough, and decreased appetite. 24% of subjects have irAEs ≥ grade 3. The overall adverse reaction is slighter than conventional chemotherapy. In a phase II clinical trial of patients with relapsed/refractory classic Hodgkin's lymphoma who failed of two or more systemic treatments, the subjects are scheduled to be injected with Camrelizumab every two weeks. The response rate is 31.8%. In another study of nasopharyngeal carcinoma treated with Camerelizumab alone or plus gemcitabine and cisplatin, both treatments show satisfactory results. In Camerelizumab group, the overall progression rate is 34%, and the half-year progression-free survival rate is 48.2%. In Camrelizumab plus gemcitabine and cisplatin group, in which the subjects are treated with 6 courses of 200 mg of Camrelizumab, 1 g/m^2 of gemcitabine, and 80 mg/m^2 of cisplatin and followed by 200 mg of Camrelizumab, the overall progression rate is 91% and half-year progression-free survival rate is 86.4%. The above results indicate that Camrelizumab is safe as well as feasible.

2. Purpose

The purpose of this study is to investigate the safety and feasibility of neoadjuvant PD-1 blockade alone or neoadjuvant PD-1 blockade plus TPF induction chemotherapy in subjects with resectable local advanced OSCC. And on this basis, we will explore the changes of the profiles and functions of immune cells within tumors, lymph nodes and peripheral blood after theexperimental

interventions, as well as their correlation with the patients' response and prognosis.

3. Objective

3.1 Primary endpoint

To investigate the major pathological response of resected tumors and lymph nodes to neoadjuvant PD-1 blockade alone or neoadjuvant PD-1 blockade plus TPF induction chemotherapy.

3.2 Secondary endpoint

To investigate the clinical response of tumors and lymph nodes to neoadjuvant PD-1 blockade alone or neoadjuvant PD-1 blockade plus TPF induction chemotherapy, as evaluated by radiographic examinations and defined by RECIST 1.1.

To investigate the two-year event-free survival (EFS) on each treatment group. EFS is the time from the date of randomization to the date of first record of disease progression as defined by RECIST 1.1.

To investigate the two-year overall survival on each treatment group.

To investigate the irAE rate. Number of participants experiencing any sign, symptom, disease, or worsening of preexisting conditions temporally associated with the experimental interventions or irrespective of the experimental interventions.

3.3 Exploratory endpoint

3.3.1 To explore the changes of the profiles and functions of immune cells within tumors, lymph nodes and peripheral blood after the experimental interventions, as well as their correlation with the patients' response and prognosis.

3.3.2 To evaluate the changes of the profiles and functions of extracellular vesicles within tumors, saliva and peripheral blood after the experimental interventions, as well as their correlation with the patients' response and prognosis.

3.3.3 To investigate the sensitivity and specificity of specific subsets of extracellular vesicles in saliva or peripheral blood of patients and their key biomolecules as liquid biopsy markers to predict the efficacy of neoadjuvant therapy or to dynamically monitor its efficacy.

4. Study design and treatment plan

4.1 Recruitment

Subjects will be recruited through the Department of Oncology, Wuhan University

Stomatological Hospital after being approved by the Ethics Committee of Wuhan University Stomatological Hospital and completing the procedures of clinical trial platform registration and Hubei Provincial Health Commission registration. Each subject will be assigned an ordered trial number (A-001, A-002, etc.) randomized into two groups by the electronic data acquisition system.

4.2 Determination of Eligibility

After eligibility is established, the study staff will register participants. The following are required to be submitted for successful registration:

a) Registration form;

b) Copy of informed consent;

c) Copy of pathological diagnosis report;

d) CT or PET/CT report, copies of chest CT and ECT;

e) Copy of clinical data:

-Routine blood examination including hemoglobin, red blood cell count, white blood cell count, granulocyte count, lymphocyte count, platelet count;

-Blood biochemistry including alkaline phosphatase, urea nitrogen, creatinine, fasting blood glucose, potassium, sodium, and chlorine;

-Hepatitis B virus surface antigen, hepatitis B surface antibody, hepatitis B e antigen, hepatitis B e antibody, hepatitis B core antibody;

-Serum thyroid hormone determination: Thyroid-stimulating hormone (TSH), patients with endocrine abnormalities should receive care or additional examinations according to the normal program.

4.3 Study design and toxicity assessments

4.3.1 Study design and sample collection

This study is a single-center, dual-arm, randomized open clinical trial, which will be conducted in the Department of Oral and Maxillofacial-Head and Neck Tumor Surgery, Wuhan University Stomatological Hospital. 68 people will be enrolled in this trial, and the subjects included are mainly from the oral and maxillofacial-head and neck tumor surgery. 68 Subjects will be randomly and averagely divided into two groups, Camrelizumab alone group (hereinafter referred to as the single group) and Camrelizumab plus TPF chemotherapy group (hereinafter referred to as the combined

group):

Single group: Camrelizumab injection, surgical resection, radiotherapy/chemoradiotherapy.

Combined group: Camrelizumab injection plus TPF induction chemotherapy, surgical resection, radiotherapy/chenmoradiotherapy.

For Camrelizumab injection, subjects will receive 200 mg Camrelizumab each 2-week cycle for 3 cycles prior to surgery. And for TPF induction chemotherapy, subjects will receive 75 mg/m2 docetaxel (T), 75 mg/m^2 cisplatin (P) and 750 mg/m^2 5-Fluorouracil (F) each 3-week cycle for 2 cycles.

Serial blood and saliva collection of the subjects will be performed prior to each Camerelizumab injection, once prior to surgery, once prior to radiotherapy, and each follow-up. Preoperative tumor biopsy and postoperative tumor lesion will be obtained for further study. The tissue biopsy specimens should be of a certain size to meet the volume requirements of rapid frozen examination and genomic analysis. The surgically resected specimen should contain tumor tissue and normal mucosa adjacent to the tumor. If there is cervical lymph node metastasis, it should contain cervical lymph nodes. The tissue should be embedded and fixed as soon as possible, or frozen in liquid nitrogen for subsequent analysis.

4.3.2 Toxicity assessments

Researchers will evaluate the safety of the subjects' toxicity from the start of the injection to 90 days following the last dose of Camrelizumab. No dose delays due to toxicity will be permitted for patients enrolled on this study. If toxic side effects occur after the first injection of the drug and the patient cannot receive the second injection on time, the patient will begin to undergo surgical evaluation and begin subsequent treatment. Before surgery, make sure that the patient is in a irAE grade $\leqslant$ 1.

4.4 Diagnostic and surgical evaluation

4.4.1 Diagnostic evaluation and pre-surgicalworkup

Subjects should obtain tissue biopsy to determine the nature of the disease before receiving drug treatment, and the specimen for tissue biopsy should be of a certain size to meet the volume requirements for rapid freezing examination and genomic analysis at the same time.

The surgically resected specimen should include tumor tissue and normal mucosa adjacent to

the tumor. If there is cervical lymph node metastasis, the cervical lymph node should be included. Tissue should be embedded and fixed as soon as possible, or frozen in a liquid nitrogen tank for subsequent analysis.

4.4.2 Diagnostic evaluation and pre-surgical workup

All subjects included in the experimental study must be patients with clinical stage III to IVb OSCC. The patient will undergo imaging tests before surgery to assess the progression of the disease and the possibility of surgical resection. The surgeon or anesthesiologist evaluates the risk of surgery based on the patient's examination report within 7 days before surgery. Surgery for patients enrolled on this protocol will be according to generally accepted standards of care.

4.4.3 Principle of surgery

Tumor resection should strictly follow the NCCN Head and Neck Cancer Guidelines (Second Edition, 2018) for the resection of the primary oral squamous cell carcinoma and neck lymph node dissection, and follow-up treatment based on the results of surgery and pathological examination (Figure 1).

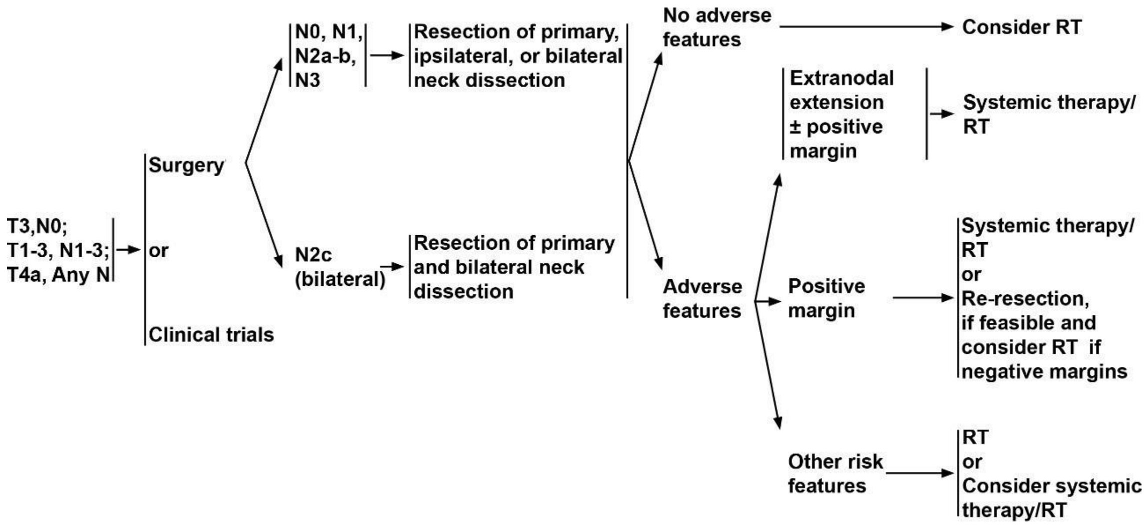

Fig 1. NCCN guideline, version 2, 2018. Cancer of the oral cavity.

4.5 Adjuvant therapy

4.5.1 Postoperative radiotherapy

All subjects will receive postoperative adjuvant radiotherapy within 4-6 weeks after surgery. Radiotherapy should be started as soon as possible for patients with high risk of recurrence, unless the patient's general condition is poor and cannot tolerate radiotherapy. Even if the wound is partially healed, the skin flap is poorly healed and the tracheal intubation or nasogastric tube is not removed,

the radiotherapy time should not be postponed, so as not to affect the prognosis. The dose of radiotherapy should be adjusted according to the site of cancer and lymph node metastasis.

4.5.2 Postoperative chemotherapy

After surgery, patients with the following high-risk and adverse prognostic factors should be supplemented with chemotherapy concurrently with radiotherapy: positive tumor margin (less than 5 mm or tumor cells are seen on the margin); extracapsular lymph node metastasis. Of note, postoperative chemotherapy should not be started until camrelizumab-related toxicity is less than grade 2.

4.6 Perioperative safety assessment

From the start of treatment to the last injection of camrelizumab within 90 days, or within 30 days after the completion of the operation, the patient’s medical records should be reviewed once a week to evaluate the complications of the operation, guide whether to delay the operation and assess potential immune-related side effects. irAE will be discussed in regular meetings held every one to two weeks, and the discussion time and content of the meeting will be recorded. If the subject refuses to continue his or her perioperative treatment due to personal reasons, every attempt will be made to collect this information either by direct contact or through communication with the subjects outside physician.

5. Laboratory research

5.1 Tissue specimen

Specimens of subjects will be collected in unified standards. At least 0.5 cm^3 of tissue biopsy should be incised to meet the requirements of frozen biopsy and other examinations. 10 mL of blood and 10 mL of saliva should be collected in the morning without eating. The time points of blood and saliva collection include: the day on each Camrelizumab injection, 3 days (+/-2 days) before surgery, 3 days (+/-2 days) before radiotherapy, every follow-up. The surgically resected specimens should retain at least 1 cm^3 of fresh tumor tissue and 0.5 cm^3 of adjacent mucosal tissues, and cryopreserved in -80°C refrigerator for subsequent research.

5.2 Handling of biopsy specimen

The tumor tissue biopsy specimen is divided into three pieces, one is used for frozen biopsy to diagnose the nature and pathological classification of the disease, one is used for

immunohistochemical to detect markers of cell and molecular, and the last one is stored in a liquid nitrogen tank for future RNA or DNA analysis.

The tumor tissue biopsy specimen should be fixed in 10% formalin or 4% paraformaldehyde solution within 30 minutes after being isolated for no less than 12 hours, and the tissue should be embedded within 24 hours. The slice thickness is 4 μm, and at least 15 slices should be cut. The slice should be clearly marked with the patient's study number, date and time, and consecutive sample numbers.

5.3 Handling of surgical tumor specimen

The surgical tumor specimen should be processed within 30 minutes and be divided into two pieces of tissue not less than 0.5 cm*0.5 cm in size: one piece is immediately lysed into a single cell suspension, and stored frozen according to the corresponding experimental manual; the other part tissue directly frozen in liquid nitrogen tank for RNA sequencing. The remaining tissues and a part of the lymph nodes are embedded, sliced and labeled according to the method of fixing tissue biopsy specimens and sent to the tissue chip.

5.4 Handling of blood

Whole blood will be collected and processed per manufacturer's instructions of PMBC. Aliquot the upper plasma into 500 μL/tube and store it at -80 °C for later ELISA or Western Blot analysis; Aliquot the middle PBMC into 500 μL/tube and freeze it in liquid nitrogen for subsequentstudy.

5.5 Handling of saliva

Saliva will be collected and centrifugated at 2,600 g, for 15 min, the supernatant will be centrifugated at 2,600 g for 15 min. Aliquot the supernatant into 500 μL/tube and store it in -80 °C nitrogen for subsequent study.

6. Screening and discontinued /withdrawal

This trial is open to all races and ethnicities, and this clinical trial does not discriminate against gender or race.

6.1 Inclusion criteria

1. Histologically documented oral squamous cell carcinoma (biopsy required).
2. Local advanced oral squamous cell carcinoma (clinical stage T1-2N1-2M0, T3-4aN0-2M0) with resection option for potential cure, as assessed by a faculty surgeon at Hospital of Stomatology, Wuhan University.
3. Distant metastasis was excluded by chest CT and emission computed tomograph.

4. Adequate organ function as follows:

1) Leukocyte count ⩾ 2,000/mm3
2) Absolute neutrophil count ⩾ 1,000/mm3
3) Platelet count ⩾ 100,000/mm3
4) Hemoglobin ⩾ 90 g/Ld
5) Serum albumin ⩾ 30 g/L
6) Total bilirubin ⩽ 1.5 × upper limit of normal (ULN)
7) AST (SGOT) and ALT (SGPT) < 2.5 ×ULN
8) ALP ⩽ 2.5 × ULN
9) Prothrombin time-international normalized ratio ⩽ 1.5
10) Serum creatinine ⩽ 1.5 × ULN
11) INR/PT⩽ 1.5
12) TSH ⩽ ULN.

5. ECOG performance status 0-1.

6. Female patient tested HCG negative in serum or urine within 7 days prior to the start of investigational product. Both patient and partner must agree to use contraception prior to study entry and for the duration of study participation and for up to 120 days after the last dose of PD-1 blockade.

Patient understands the study regimen, its requirements, risks and discomforts and is able and willing to sign the informed consent form.

6.2 Exclusion Criteria

1. History of ⩾ 3 grade immune related adverse events (irAEs) or have notrecovered to ⩽ 1 grade irAEs from previous treatment.
2. History of other treatments for cancer, including surgery, chemotherapy, radiotherapy or molecular targeted therapy within past 5 years.
3. Previous therapy with anti-PD-1, anti-PD-L1, anti-PD-L2, anti-CTLA-4 or any other antibody targeting T cell co-regulatory pathways.
4. Active autoimmune disease or history of refractory autoimmune disease.
5. Active systemic infection requiring therapy.
6. Patients who are receiving psychotropic drug or alcohol/drug abuse.
7. Subjects with concurrent other active malignancies.
8. HIV or untreated active HBV or HCV infections, or vaccinated (HBV, flu, varicella, etc) within 4 weeks before recruitment.
9. Uncontrollable systemic diseases, including diabetes, hypertension, etc.
10. History of stroke or transient ischemic attack within past 6 months.

6.3 Withdrawal from study

1. The reasons for withdrawal from the study include:
2. The subject withdraws the informed consent and asks to withdraw;
3. Those who cannot tolerate the toxicity of TPF chemotherapy or Camrelizumab immunotherapy;

4. The subjects have poor compliance;

5. The subject is lost to follow-up or lost contact;

6. The subject has a sudden illness or other situation during the experiment, and the situation will greatly affect the evaluation of the clinical state or the study treatment needs to be stopped;

7. Subjects are followed up for two years;

8. The researcher believes that it is necessary to withdraw the subject from the research.

Note: If subjects experience ultra-progressive imaging or serious immune-related adverse reactions after the first neoadjuvant immunotherapy, the second neoadjuvant immunotherapy should be stopped, and the patient's status should be adjusted directly before surgery and subsequent treatment. For subjects who are discontinued/withdrawn, the investigator must make every effort to complete the validity and safety inspections when launching the trial specified in the protocol, complete the safety follow-up, comprehensively record the adverse events and outcomes; complete the survival follow-up and record follow-up treatment plan and subject's survival status. The reason for treatment interruption should be carefully recorded in the medical record. Researchers can suggest or provide new or alternative therapies to subjects based on the patient's condition. If the subject interrupts or withdraws from the study, the researcher should collect blood, saliva and other specimens as much as possible with the consent of the subject.

7. Study procedures

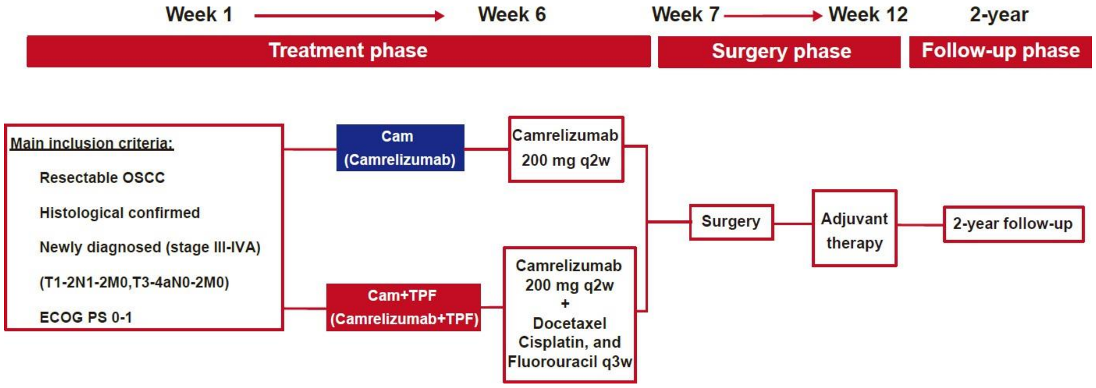

7.1 Clinical operation schedule

7.1.1 Screening stage, 1-7 days prior to enrollment

1. Clinical examination and taking pictures to record the lesions, the lesion should meet T1-2N1-3M0, T3-4aN0-3M0;

2. Systemic examination to exclude the contraindications of surgery;

3. Take enhanced CT/MRI records of head and neck region to record the tumor status, and take emission computed tomography to exclude distant metastasis;

4. Tumor biopsy;

5. 10 ml of blood and saliva collection;

6. Subject should sign the informed consent form, then researcher submit the basic information to the electronic data collection system and divide the subject into a treatment group through the randomization system.

7.1.2 Neoadjuvant stage, day 1-40 (+/-7 days) after enrollment

1. Neoadjuvant immunotherapy

200 mg of Camrelizumab injection at the time points of day 1, day 15 and day 29.

2. TPF induction chemotherapy

Day 1: Deep venous catheterization (no chemotherapy drug injection on the same day, only for camrelizumab).

Day 2:

a. 5% GNS 1000 ml + 10% KCl 10 ml iv gtt (6-8 h prior toCDDP);

b. Normal saline 50 ml + 5-HT3 receptor antagonist (such as ondansetron 8 mg or glastron 3 mg or tropisetron 5 mg) iv gtt (30 minutes prior to chemotherapy, additional one administration is needed if severe nausea occurs);

c. Dexamethasone 5 mg iv + Promethazine 25 mg im (docetaxel pretreatment);

d. Normal saline 500 mg + docetaxel 75 mg/m^2 iv gtt for 2-3hours;

e. Normal saline or glucose solution 500 ml + 10% KCl 10 ml + dexamethasone 5 mg iv gtt for 3 hours;

f. 20% mannitol 50 ml iv (before cisplatin use);

g. 5% glucose solution 500 ml + CDDP 75 mg/m^2 iv gtt for 1-2h;

h. 20% mannitol 50 ml iv q1h×5 times;

i. Normal saline or glucose solution or Ringer's solution 1000 ml + 10% KCl 10 ml +

dexamethasone 5 mg iv gtt for 3 hours;

j. Normal saline 500 ml + 5-FU 750mg/m^2/d×5 d iv gtt;

k. Ringer's solution 500 ml + vitamin C 2 g + vitamin B6 200 mg ivgtt;

l. Other auxiliary drugs such as hepatinica.

Day 3:

a. 1500 ml normal saline or glucose solution + 10% KCl 20 ml + dexamethasone 10 mg iv gtt;

b. 20% mannitol 50 ml iv q1h×3 times;

c. Normal saline 50 ml + central nervous antiemetics (5-HT3 receptor antagonists, such as ondansetron 8 mg or granisetron 3 mg or tropisetron 5 mg) iv gtt (1-2 times a day or according to the severity of nausea and vomiting);

d. Ringer's solution 500 ml + vitamin C 2 g + vitamin B6 200 mg ivgtt;

e. Other auxiliary drugs such as hepatinica.

Day 4-6:

a. Normal saline 50 ml + central nervous antiemetics iv gtt;

b. Ringer's solution 500 ml + vitamin C 2 g + vitamin B6 200 mg ivgtt;

c. Other auxiliary drugs such as hepatinica.

Days 7-21: Rest (the patient needs to return to the hospital for the second camrelizumab injection on day 15).

Day 22-26: Repeat the content of Days 1-6 and perform a second course of TPF chemotherapy.

Day 27-42: Rest (return to the hospital on day 29 for the 3rd camrelizumabinjection)

7.1.3 Surgery stage

Preoperative examination, 1-3 days prior to surgery.

1. Clinical examination and taking pictures to record the lesions;

2. Take enhanced CT/MRI records of head and neck region to record the tumor status, and take emission computed tomography to exclude distant metastasis;

3. 10 ml of blood and saliva collection;

Surgery day

1. Take pictures to record the operation method and the number of separated lymph nodes.

2. Collect tumor specimen and store it as described above.

7.1.4 Adjuvant therapy stage, 30 days (+/-10 days) postoperation

Prior to radiotherapy

1. Clinical examination and enhanced CT/MRI examination.

2. 10 ml of blood and saliva collection.

3. Design the plan of radiotherapy according to surgical records, silver clip markers and pathology reports.

4. Concurrent chemoradiotherapy:

a. Subjects with the following high-risk and poor prognostic factors: positive resection margin (less than 5mm or visible tumor cells on the resection margin); extracapsular invasion of lymph nodes.

b. The concurrent chemoradiotherapy plan: The subject will receive CDDP 80 mg/m^2 administration each 3-week cycle for 2 cycles.

7.1.5 Follow-up stage

The first follow-up will start at 90 days (+/-10 days) after the end of radiotherapy with an interval of 3 months (+/-10 days) for a total of 7 follow-ups. The contents of follow-up include:

①Clinical examination.

②10 ml of blood and saliva collection.

③Head and neck enhanced CT/MRI examination, performed at the 1, 3, 5, and 7 visits.

④Assessment, management and record of the irAEs, and analyze the correlation between the irAEs and the study drug. Once identified as irAE, it should be submitted to National Medical Products Administration in time.

It is worth noting that if the patient cannot be followed up on schedule, the patient should return to hospital within two months, and the follow-up will continue as scheduled. If the patient cannot be followed up for three consecutive times, it should be considered as drop-out, and the reason for drop-out should be recorded. Though the data of drop-out patient cannot be used for the analysis of laboratory indicators for dynamic monitoring of the patient's condition, it can still be used to evaluate the safety and effectiveness of neoadjuvant immunotherapy.

7.2 Specimen processing schedule

7.2.1 Screening stage

①Collect the biopsy specimen and divide it into two pieces, one piece will be frozen with RNAlater nucleic acid preservation solution in liquid nitrogen, and the other one will be used for frozen biopsy and then taken out to be re-fixed with formalin and embed in paraffin;

②Collect 10 ml blood (EDTA anticoagulation vacuum tube) and 10 ml saliva from the subject before eating in the morning. Whole blood will be collected and processed per manufacturer's instructions of PMBC. Aliquot the upper plasma into 500 μL/tube and store it at -80 °C for later analysis; aliquot the middle PBMC into 500 μL/tube and freeze it in liquid nitrogen for subsequent study. Saliva will be collected and centrifugated at 2,600 g, for 15 min, the supernatant will be centrifugated at 2,600 g for 15 min. Aliquot the supernatant into 500 μL/tube and store it in -80 °C nitrogen for subsequent study.

7.2.2 Surgery stage

On the first day after admission, collect the patient's blood and saliva, the method is described as 7.2.1;

On the surgery day, collect about 1×1×1 cm^3 of the most representative tumor and divide it into 3 pieces. One piece is prepared for single cell suspension following the, then frozen in a liquid nitrogen tank, and one piece is fixed with formalin, embedded in paraffin, add a piece of RNAlater nucleic acid preservation solution and freeze it in a liquid nitrogen tank.

7.2.3 Adjuvant chemoradiotherapy stage

On the first day after admission, collect the patient's blood and saliva, the method is described as 7.2.1.

7.2.4 Follow-up stage

After adjuvant radiotherapy, patients will have regular follow-up visits every 3 months for a total of 7 visits for two years. Blood and saliva will be collected every time, the method is described as above as 7.2.1, but fasting collection is not required.

8. Study drug

8.1 Drug description

Camrelizumab will be ordered from Hengrui Pharmaceutical Co., Ltd. Each bottle of 200mg can only be used once. The drug label is shown in Table 1.

Table 1				
Product description	Specifications	Size	Appearance	Storage
Lyophilized powder of camrelizumab	200 mg	20mL injection bottle	Transparent glass bottle, white powder	2-8 ℃, keep away from light and freezing

8.2 Pharmacokinetics of camrelizumab

A phase I study evaluated the pharmacokinetics of camrelizumab in 49 patients with advanced solid tumors in China at different doses (1 mg/kg, 3 mg/kg, 10 mg/kg and 200 mg/time). The study showed that the in vivo exposure (Cmax) of camrelizumab increased with the increase of dosage in the range of 1-10 mg/kg after a single administration. After multiple administration (once every 2 weeks), the average steady-state accumulation ratio calculated according to Cmax on the 15th day of the fourth cycle is between 1.11-1.35.

8.3 Safety summary and side effects of camrelizumab

The comprehensive treatment measures of potential camrelizumab related toxic and side effects are recorded in detail in the annex and the researcher's manual. Combined with previous clinical trials, more than 10% of the side effects were mainly reactive capillary hemangioma (79.3%), anemia, fever, fatigue, hypothyroidism, proteinuria and cough; 1% - 10% of side effects include anemia, hypernatremia, lung infection, elevated aspartate aminotransferase level, elevated glutamyltransferase level, elevated blood bilirubin level, elevated bilirubin level, abnormal liver function, decreased neutrophil count, decreased leukocyte count, thrombocytopenia, decreased lymphocyte count, hypokalemia, increased alanine aminotransferase level, pneumonia, increased blood alkaline phosphatase level and increased lipase level.

8.4 Disposal and distribution of camrelizumab

Camrelizumab is managed by a specially assigned person set up by the main investigator of the clinical trial and stored in a separate refrigerator for the clinical trial. The drug administrator shall ensure that the drug is stored in a suitable environment (temperature, light, humidity). If there is any doubt about the quality and appearance of the drug, the supply company should be contacted directly. The bottles of camrelizumab should be stored in a refrigerator at 2-8 ℃ away from light and avoid freezing. If it is stored in a glass cabinet refrigerator, it should be stored in the packing box. It is recommended to wear work clothes and gloves when transporting or preparing drugs.

When distributing drugs, the drug receiving form shall be signed by two persons in duplicate. The clinical research unit and the application department shall supervise the supply, use and storage of drugs and the treatment process of remaining drugs.

8.5 Injection method of camrelizumab

The patients in the test group will be injected with carrelizumab once on day 1, day 15 and day 29, with 200 mg each time. Dilute the drug into 100 ml glucose or sodium chloride injection. Infusion should be completed within 30-60 minutes.

Camrelizumab does not contain preservatives, so aseptic operation is needed. Each bottle of camrelizumab for injection should be re dissolved with 5 ml of sterile water for injection. Avoid directly spraying the water droplets on the surface of the powder, and slowly add it along the bottle wall, slowly swirl it to dissolve, and put it to the foam to dissipate, and do not shake violently. The solution shall be colorless or yellowish liquid. If visible particles are observed, stop the injection and replace the drug. Transfer 5 ml of the solution to the infusion bag containing 100 ml of glucose injection (5%) or sodium chloride injection (0.9%), and conduct intravenous infusion through a sterile, pyrogen free and low protein binding 0.2 μM filter.

Camrelizumab should be dissolved and diluted immediately after removal from the refrigerator. Store the diluted solution at room temperature for no more than 6 hours

(including infusion time): store it under refrigeration (2-8 ℃) for no more than 24 hours. If the diluted solution is stored under refrigeration, it should be restored to room temperature before use. Note that this drug should not be administered simultaneously with other drugs.

8.6 Destruction of research drugs

The investigator shall ensure that the drug is destroyed and archived in accordance with relevant regulations, guidelines, systems and procedures.

9. Safety evaluation

9.1 Safety summary

This study will use the description and grading scale of adverse event reports in the revised National Cancer Institute Common Terminology Standard for adverse events (CTCAE) version 5.0. Information on all adverse events, whether voluntarily provided by the subject, found through the inquiry of the investigator, or detected through physical examination, laboratory test or other means,

will be collected, recorded and tracked.

All adverse events experienced by subjects will be collected from the first medication, throughout the whole study process, and at least 90 days after the last treatment. Subjects who still have toxic and side effects after discontinuing the study drug should be further evaluated and followed up until the toxic and side effects disappear or are determined to be irreversible. Any preoperative and postoperative adverse events should be carefully recorded.

9.2 Adverse event

9.2.1 Definition of adverse events

Adverse event refers to any new unpleasant medical event or deterioration of previous symptoms during the use of the drug by the patient or subject, and the drug does not necessarily have a causal relationship with the treatment. Therefore, adverse events can be any adverse and unexpected manifestations (including abnormal laboratory results), symptoms or diseases temporarily related to the study drug.

Adverse events can be reported or described in the process of open inquiry, examination or evaluation at the same time. To prevent reporting bias, subjects should not ask about the specific circumstances of one or more adverse events. The medical condition / disease occurring before the start of the study treatment is considered an adverse event only when the condition worsens after the start of the study treatment (any step in the treatment procedure). Although it happened before treatment, the adverse events after signing the informed consent should also be recorded. Abnormal laboratory results or adverse events caused by them constitute adverse events only when they cause clinical symptoms or need treatment.

9.2.2 Serious adverse events (SAE)

① Serious adverse events refer to the following adverse clinical consequences:

a) Death (excluding death caused by tumor recurrence or metastasis);

b) Life-threatening (it means that the subject is in danger of life, not that it will be life-threatening if it worsens further);

c) Need to be hospitalized or extended;

d) Cause persistent or significant disability or dysfunction;

e) Important clinical events (Although it will not immediately endanger life, cause death or

cause hospitalization, according to reasonable clinical diagnosis, the event will endanger the subject's life or require intervention, otherwise the consequences listed above will occur. These events include, but are not limited to, intensive treatment for allergic bronchospasm in the emergency or at home, vasospasm or convulsion without hospitalization. Potential drug-induced liver injury is also listed as a serious medical event)

② The following hospitalizations are not serious adverse events:

a) Not more than 24 hours in emergency or other medical departments, except for "important medical events" or life-threatening events;

b) Scheduled surgery before signing informed consent;

c) Treatment or surgery according to the trial process;

d) Routine medical examination (such as routine colonoscopy);

e) Admission treatment planned before enrollment, but its purpose is not to treatdiseases;

f) The subjects were hospitalized due to the impact of their personal living environment (inability to housing, lack of economic capacity, etc.).

9.2.3 Unexpected adverse events

Any adverse events not covered in the informed consent form are unexpected adverse events.

9.2.4 Expected adverse events

The adverse events involved in the investigator's manual and informed consent documents are considered as expected adverse events.

9.3 Sorting and reporting of serious adverse events

After the subject signs the informed consent to participate in the clinical trial study, whether it is related to the study drug or not, all serious adverse events should be sorted and collected, including those that may be related to the treatment procedure.

Within 24 hours after the occurrence of serious adverse events, the researcher shall report to the ethics committee, the provincial drug administration, Safety Supervision Department and Registration Department of the State Drug Administration and the local health administrative department in time. All reports shall have written materials for inspection and verification, and shall be archived as test data.

9.4 Non-serious adverse events

Non-serious adverse events refer to the types of adverse events that do not belong to serious adverse events.

All non-serious adverse events should be recorded during treatment and at least 90 days after the last injection treatment. Non-serious adverse events should be tracked to whether they are resolved and whether the symptoms persist or turn into serious adverse events. Non-serious adverse events leading to the interruption or withdrawal of study drugs or adverse events at the end of treatment also need to be followed up as appropriate. All non-serious adverse events need to be carefully recorded in the paper or electronic medical record system.

9.5 Abnormal laboratory test results

For adverse events and laboratory abnormalities found during the study, the case report form should be improved. Serious adverse event case report (paper or electronic) shall be reported as appropriate.

a) Laboratory test results of any serious or serious adverse events;

b) Any abnormal index of laboratory results requiring drugwithdrawal;

c) Any abnormal index of laboratory results requiring special treatment;

Researchers should try to use clinical terms rather than laboratory terms, such as anemia and low hemoglobin.

9.6 Gestation

If the subject finds pregnancy after the start of the study, including within six half-lives after drug injection, the subject will permanently stop the clinical trial. Unless pregnancy is contraindicated, the subject must undergo the prescribed study suspension and follow-up procedures. If necessary, other appropriate pregnancy follow-up procedures should be considered. The researcher should report to the ethics committee of School and Hospital of Stomatology Wuhan University and Hubei Food and Drug Administration within 24 hours.

Follow up information on the pregnancy process, including perinatal and neonatal outcomes, must be reported on the pregnancy monitoring form. Any pregnancy that occurred in a female partner of a male subject should also be reported to the investigator. The information about this pregnancy should be reported to the ethics committee of School and Hospital of Stomatology Wuhan University.

9.7 Overuse

Overuse refers to conscious or unconscious overuse. All overuse should be reported as serious adverse events.

9.8 Other safety precautions

The deterioration found in the mid-term or final physical examination, ECG, X-ray examination and other potential risk assessment (whether it belongs to the examination in the test process or not) shall be recorded as non-serious or serious adverse events and reported as appropriate.

10. Effectiveness evaluation

The objective remission rate (imaging and pathological remission rate), recurrence rate, progression free survival rate, one-year survival rate and overall survival rate were evaluated.

In this clinical trial, all subjects underwent imaging examination at baseline, before operation and every two months after operation, and all subjects underwent pathological biopsy and surgical pathological biopsy. The clinical tumor imaging evaluation was carried out through the solid tumor efficacy evaluation standard version 1.1 (see the research manual), and the pathological grading was carried out by hematoxylin eosin staining of pathological sections.

All patients' imaging examinations (enhanced CT or MRI) were confirmed by independent imaging experts in a blind manner. All subjects with curative effect evaluation of CR, PR, SD and PD shall be reviewed by the person in charge first, and the imaging data of all curative effect evaluation (image film or image examination CD) shall be retained, and finally evaluated and confirmed by independent imaging experts. Similarly, the pathological examination of all patients is confirmed by independent pathologists in a blind manner. Surgical specimens included primary lesion and lymph nodes were collected and fixed by formalin. After recognition of the tumor bed, primary lesion was dissected every 3 mm parallel to the max section. Slices were numbered and registered from mesial to distal during sampling. Slices of lymph nodes were collected from every single lymph node and numbered. Hematoxylin and eosin (H&E)-stained slides of entire tumor and all sampled lymph nodes were scanned. For the subjects evaluated as pathological complete remission (pCR), pathological partial remission (pPR) and non-pathological remission (Non-resp), the person in charge shall review and retain all evaluated pathological data (microscope photos or pathological sections). Finally, they should be evaluated and confirmed by independent pathological experts. The exploratory research on the tissue, blood and saliva samples will be

carried out by the laboratory personnel in a blind state, and the blindness will be uncovered after the experimental data are obtained.

11. Exploratory study

11.1 Tissue and body fluid analysis

Tumor cells, infiltrating lymphocytes, and other cells in the tumor microenvironment (such as tumor-associated fibroblasts, lymphatic endothelial cells, vascular endothelial cells, and vascular endothelial cells) and their surface molecular changes were compared before and after treatment using immunohistochemistry, flow cytometry, and other methods.

Tumor samples were sequenced for DNA or RNA before and after therapy to assess tumor mutational burden (TMB), microsatellite instability (MSI), and mismatch repair (MMR).

Flow cytometry, ELISA, Western blotting, and other techniques were used to assess extracellular vesicles (EVs), lymphocytes, and the molecules they contain in blood and saliva, as well as to dynamically monitor the changes at various treatment phases.

11.2 Screening of immune response markers for anti-PD-1 therapy

The researchers looked at the associations between the patients' baseline markers, especially the PD-L1 composite positive score (PD-L1 CPS), MSI, MMR, and EVs subgroup molecules, and the efficacy of neoadjuvant treatment.

The changes of each indicator before and after treatment were studied, as well as the relationship between them and the efficacy of neoadjuvant therapy and the patients' prognosis.

Screen the indicators with clear clinical significance and possible biomarkers from the above data, and evaluate their sensitivity and specificity as tumor diagnostic markers or treatment effectiveness markers, as well as their practicality as biomarkers for predicting immunotherapy efficacy.

11.3 Indicators to be detected

This study's preliminary design includes the detection of tumor cells, immune cells, monocytes, extracellular vesicles, and their subgroups.

Tissue biopsies and surgically resected specimens, as well as drainage fluids, blood, and saliva from patients with oral squamous cell carcinoma, will be analyzed for immune response markers.All samples were analyzed using multicolor flow cytometry, immunohistochemistry and in situ hybridization techniques to look for candidate immune response markers related to anti-PD-1

therapy, including expression in peripheral blood lymphocytes, tumor-infiltrating lymphocytes, lymph nodes, and tumor cells.

Table 2 Protein molecular detection

Molecular	Molecular function
CD4, CD8	Involved in the interaction of T cell receptors with antigen presenting cells
CD25	IL-2 receptor, involved in suppressing antitumor immunity
HLA-DR	Expression of immune recognition antigens
CD45RO	Enhance anti-tumor immunity
FoxP3	It plays an important role in the formation of regulatory T cells and participates in the inhibition of anti-tumor immunity
LAP	Potential markers of activated regulatory T cells
PD-1/PD-L1	Inhibition of anti-tumor immunity, PD-1 is the therapeutic target of camrelizumab
LAG-3	Collaborate PD-1 suppresses anti-tumor immunity
ICOS	Stimulates T cells to act on tumor cells
CTLA-4	Immune checkpoint molecules that suppress antitumor responses

12. Management of adverse events

12.1 Infusion reaction

There have been reports of camrelizumab infusion reaction during injection, and clinical symptoms and signs should be closely monitored following treatment. Fever, chills, stiffness, headache, rash, pruritus, Alsace illness, hypotension, hypertension, bronchospasm, or other allergic-like events might happen as a result of infusion reactions. For patients with Grade 1 infusion reactions, therapy can be continued under close monitoring; If an infusion reaction of grade 2 occurs, the infusion rate can be slowed or the infusion can be stopped. Antipyretic and analgesic anti-inflammatory drugs, and also antihistamines, may be recommended. When the symptoms have subsided, the medicine can be restarted but must be closely monitored. Grade 3 or higher infusion responses must be stopped instantly and reported as serious adverse events (SAEs). Infusion reactions were graded according to the NCI CTCAE (version 5.0) guidelines. Recommendations for the treatment of infusion reactions are as follows:

Grade 1 infusion reaction： A mild reaction that does not need stopping the infusion. Keep an eye on things until the symptoms disappear. In the future, 30 minutes before re-dosing, take 50 mg diphenhydramine and（or） paracetamol/acetaminophen 325-1000 mg orally.

Grade 2 infusion reaction: A moderate reaction that necessitates treatment or suspension of

infusion, and requires symptomatic medication, such as antihistamines, nonsteroidal anti-inflammatory drugs, anesthetics, corticosteroids, intravenous infusion, and so on; preventive medication can be given up to 24 hours in advance. Stop the camrelizumab injection and replace it with intravenous saline, intravenous diphenhydramine 50 mg, or paracetamol/acetaminophen 325-1000 mg; monitor the patient attentively until the symptoms disappear. Corticosteroid treatment can also be applied in the proper situations. If the symptoms disappear， the injection can be resumed at half the initial rate of infusion. If no symptoms appear 30 minutes after the re-injection, the injection can be continued at the original infusion rate, although close monitoring is still necessary. If the infusion reaction occurs again, no further infusion treatments will be administered. In the future, 30 minutes before re-dosing, take 50 mg diphenhydramine and/or paracetamol/acetaminophen 325-1000 mg orally. Corticosteroid treatment can also be utilized as necessary.

Grade 3 or 4 Infusion reactions： Serious infusion reactions, Grade 3 reactions: symptoms that do not improve after long-term symptomatic treatment or infusion suspension; initial infusion reactions that reappear with the second infusion; other severe symptoms that require hospitalization [such as kidney damage, pulmonary infiltration].

12.2 Immune-related adverse events

Immune-related adverse events can occur during and after immunotherapy is ended, and then they can affect any tissue or organ and, in severe cases, result in the patient's death. Suspected immune-related adverse events should be fully examined to rule out other possible causes. The majority of immune-related adverse events were reversible and may be handled with drug discontinuation, corticosteroid injections, or supportive care. Most grade 2 and some specific grade 3 or 4 immune-related adverse events need drug discontinuation during therapy, and some grade 3 and 4 immune-related adverse events require permanent stop. Furthermore, if any recurring grade 3 immune-related adverse events after therapy, grade 2 or 3 immune-related adverse events that did not improve to grade 0-1 within 12 weeks from the last dose, the corticosteroid cannot be reduced to a 10 mg/day equivalent dose of prednisone within 12 weeks of the last dose, the drug should be stopped permanently. The descriptions here are based on the first version of the NCCN Guidelines for the Management of Immunotherapy-Related Toxicity, published in 2019. (Refer to the Research Manual for details).

12.2.1 Immune-Related Skin Adverse Events

Immune-related cutaneous adverse events occurred in 2.8% of camrelizumab patients, with 1.3 % having grade 1, 0.8% having grade 2, and 0.7% having grade 3. For grade 1 or 2 rash, symptomatic therapy or topical corticosteroids can be given. Camrelizumab treatment should be stopped, and symptomatic treatment or topical corticosteroids should be offered in the case of a grade 3 rash. If a grade 4 rash, Scottish-Johnson syndrome, or toxic epidermal necrolysis occurs, camrelizumab treatment should be discontinued completely.

12.2.2 Immune-related gastrointestinal adverse events

In previous treatment, the incidence of immune-related gastrointestinal adverse events was 0.9%, of which 0.7% had immune-related diarrhea, all grade 3, 0.1% had grade 3 colitis, and 0.1% had grade 5 colitis. Patients should be followed for signs and symptoms associated with immune-related gastrointestinal adverse events, such as stomach ache, diarrhea, mucus or bloody stools, and infection and disease-related causes should be eliminated in the case of immune-related gastrointestinal adverse events. Camrelizumab therapy should be deferred for grade 2 or 3 immune-related diarrhea or colitis, and should be completely discontinued for grade 4 or recurring grade 3 immune-related gastrointestinal adverse events. At the same time, the potential risk of intestinal perforation should be considered and confirmed by imaging and/or endoscopy if necessary.

12.2.3 Immune-related hepatic adverse events

On prior therapy, the incidence of immune-related hepatic adverse events was 9.1%, including 0.1% for grade 2, 7.0% for grade 3, 1.5% for grade 4, and 0.5% for grade 5. Changes in liver function and the related symptoms and signs of hepatitis should be followed once a month during regular treatment of patients, and infections and underlying disorders should be eliminated. If immune-related hepatic adverse events occur, liver function tests should be performed more often. Camrelizumab treatment should be discontinued for grade 2 immune-related hepatic adverse events. If there are grade 3 or 4 immune-related hepatic adverse events, camrelizumab treatment should be stopped completely.

12.2.4 Immune-related pancreatic adverse events

There were no immune-related pancreatic adverse events in prior therapy. The incidence of immune-related amylase or lipase increase was 1.3%. Lipase increase was 1.0%, with grade 3 at 0.6% and grade 4 at 0.4%. The amylase increase was 0.3%, all of which were grade 3.

12.2.5 Immune-related endocrine adverse events

①High blood sugar

On prior treatment, the incidence of elevated blood glucose was 1.5%, 0.8% for grade 1, 0.6% for grade 3, and 0.1% for grade 4. The incidence of diabetes was 0.2%, all of which were grade 3. Patients' blood glucose levels, as well as any related clinical signs and symptoms, should be closely monitored. Camrelizumab treatment should be deferred in type 1 diabetes patients with poor glycemic control, and insulin replacement therapy should be halted until symptoms disappear. Camrelizumab must be permanently stopped in patients with life-threatening grade 4 type 1 diabetes. Blood glucose levels should be evaluated in the future to ensure that insulin replacement treatment is effective.

②Thyroid changes

In previous treatment, the incidence of hypothyroidism was 20.5%, including 12.8% for grade 1 and 7.7% for grade 2; the incidence of hyperthyroidism was 6.7%, of which 5.7% for grade 1 and 1.0% for grade 2; The incidence of thyroiditis was 0.3%, with 0.2% for grade 1 and 0.1% forgrade 2. Patients should be closely monitored for changes in thyroid function and corresponding clinical symptoms and signs. For symptomatic grade 2-3 hypothyroidism, camrelizumab therapy should be withheld and thyroid hormone replacement therapy initiated as needed. Camrelizumab should be discontinued in patients with symptomatic grade 2-3 hyperthyroidism, and antithyroid medications should be used as needed. Camrelizumab withholding and hormonal treatment may be explored if acute thyroid inflammation is suspected. Camrelizumab treatment can be continued if symptoms of hypothyroidism or hyperthyroidism improve and thyroid function tests return to normal. If you have life-threatening hyperthyroidism or hypothyroidism, Camrelizumab should be stopped completely. Thyroid function should be evaluated in the future to ensure that hormone replacement treatment is effective.

③Adrenal gland changes

Adrenal insufficiency occurred in 0.4% of patients with previous treatment, with grade 1 in 0.1%, grade 2 in 0.2%, and grade 3 in 0.1%. Adrenal insufficiency symptoms should be closely monitored in patients. Camrelizumab treatment should be discontinued for symptomatic grade 2

adrenal insufficiency, and physiologic corticosteroid replacement therapy should be administered as clinically necessary until symptoms disappear. Camrelizumab must be stopped completely if patients have Grade 3-4 adrenal insufficiency. Adrenal function and hormone levels should be monitored continuously to ensure proper corticosteroid replacement treatment.

④Changes in the central system

Hypophysitis occurred in 0.1% of previous treatments and was grade 1. Patients with hypophysitis should be closely monitored for signs and symptoms (including hypopituitarism and secondary adrenal insufficiency) and other etiologies should be excluded. Camrelizumab should be withheld for symptomatic grade 2-3 hypophysitis, and hormone replacement treatment should be given as clinically necessary. Corticosteroids can be administered if acute hypophysitis is suspected. For life-threatening grade 4 hypophysitis, camrelizumab must be permanently discontinued. Adrenal function and corticosteroid levels should continue to be monitored to ensure appropriate corticosteroid replacement therapy.

12.2.6 Immune-related pulmonary adverse events

On prior treatment, the incidence of immune-related pulmonary adverse events was 2.7%, including 0.1% for grade 1, 0.9% for grade 2, 1.0% for grade 3, 0.3% for grade 4, and 0.4% for grade 5. Clinical symptoms and signs should be monitored during the treatment process, and suspected immune-related pneumonia cases should be confirmed by imaging examinations, with other causes being excluded out. If grade 2 immune-related pneumonitis develops, the drug should be stopped immediately, and if grade 3 or higher or recurring grade 2 immune-related pneumonitis occurs, the drug should be stopped permanently.

12.2.7 Immune-related adverse renal events

On prior therapy, the incidence of immune-related renal adverse events was 0.4%, including 0.1% for grade 2 and 0.3% for grade 3. Changes in renal function, as well as accompanying symptoms and indicators of nephritis, should be examined on a monthly basis during therapy. If immune-related nephritis develops, renal function tests should be performed more often. The vast majority of patients with high serum creatinine are asymptomatic. Other possible causes of renal impairment should be ruled out. Camrelizumab should be stopped if serum creatinine levels increase to a level of 2 or 3. A grade 4 serum creatinine increase should permanently discontinue

camrelizumab therapy.

12.2.8 Immune-related hepatic adverse events

On prior therapy, the incidence of immune-related hepatic adverse events was 9.1%, including 0.1% for grade 2, 7.0% for grade 3, 1.5% for grade 4, and 0.5% for grade 5. During the treatment, the changes of liver function and the corresponding symptoms and signs of hepatitis should be monitored every month, and infection and the cause related to the underlying disease should be excluded. If immune-related hepatitis develops, liver function tests should be performed more often. Camrelizumab treatment should be avoided in patients with grade 2 immune-related hepatitis. For grade 3 or 4 immune-related hepatitis, camrelizumab treatment should be permanently discontinued.

12.2.9 Immune-related adverse cardiovascular events

Immune-related platelet count reductions occurred in 1.7% of patients with prior therapy, including 1.2% for grade 3 and 0.5% for grade 4. Patients should be closely monitored for platelet levels and signs and symptoms of bleeding tendencies, and other causes of thrombocytopenia should be ruled out. When grade 3 thrombocytopenia occurs, camrelizumab treatment should be suspended , and supportive care should be given until the patient recovers to grade 0-1. Corticosteroid therapy should be administered based on clinical assessment and whether or not camrelizumab treatment may be begun. In the event of grade 4 thrombocytopenia, the drug should be permanently discontinued and symptomatic treatment should be taken actively, and corticosteroid therapy should be given if necessary.

The incidence of immune-related myocarditis was 0.3%, with grade 1 being 0.2% and grade 5 being 0.1%. Clinical signs and symptoms of myocarditis should be followed, and if immune-related myocarditis is suspected, an appropriate examination should be performed to confirm the etiology and rule out other possibilities, as well as necessary tests such as myocardial enzymes. Camrelizumab treatment should be stopped if grade 2 myocarditis occurs, and corticosteroid therapy should be started. The safety of continuing camrelizumab treatment after myocarditis has recovered to grade 0-1 is unknown. Patients with grade 3 or 4 myocarditis should be treated with corticosteroid medication for the rest of their lives, and their myocardial enzymes and cardiac function should be closely monitored.

12.2.10 Other immune-related adverse events

In the previous treatment, immune-related myositis (0.2%) and conjunctivitis (0.3%) also occurred. For other suspected immune-related adverse reactions, adequate evaluation should be performed to confirm the etiology and exclude other etiologies. Depending on the severity of the adverse events, camrelizumab treatment should be withheld for the first occurrence of a grade 2 or 3 immune-related adverse events. Camrelizumab treatment must be permanently discontinued for any recurrent grade 3 immune-related adverse reaction (excluding endocrine disease) and any grade 4 immune-related adverse reaction. Corticosteroids should be used only when clinicallyneeded.

13. Research Management

13.1 Amendments to the Scheme

The "Clinical Research Protocol", "Clinical Research Case Report Form" and "Investigator's Manual" are formulated by the principal investigator, and the principal investigator and all investigators discuss and formulate the clinical research plan. Any changes to the trial protocol were approved by the Institutional Review Board in the form of an amended protocol. The principal investigator will be responsible for the coordination and development of clinical trial protocol modifications.

13.2 Informed Consent

The investigator will explain to each subject the nature, purpose, procedures involved, expected duration, potential risks and benefits of the study. Each subject will be informed that participation in the study is voluntary, that personal data in the trial will be kept confidential, that he/she can withdraw from the study at any time, and that withdrawal of consent will not affect his/her subsequent treatment. Informed consent will be in the form of a standard written statement and submitted to the Institutional Review Board for approval prior to use. Only after signing the informed consent form can they be enrolled in the study. According to "Good Clinical Practice for Drugs", the contents of the signed informed consent form include the consent to authorize the subject's information to the relevant research institutions, including research sponsors, research institutions, regulatory agencies or ethics committees, and the subject's information Include all hospital admissions and medical history of the subject.

13.3 Code of Ethics and Practice for the Management of Drug ClinicalTrials

Clinical trial research shall comply with the management standards for drug clinical trials, and

follow the following contents:

a) "Good Clinical Practice for Drugs" promulgated by China Food and DrugAdministration;

b) "Management Measures for Clinical Trial Projects of Wuhan University Stomatological Hospital;

c) The International Ethical Guidelines for Human Biomedical Research issued by the International Committee of the Organization of Medical Sciences;

d) Declaration of Helsinki.

13.4 Regulatory authorities

This clinical trial was supervised by the Ethics Committee of Wuhan University Stomatological Hospital, the Clinical Research Center of Wuhan University Stomatological Hospital and the National Medical Products Administration (NMPA).

13.5 Principal Investigator (PI) Responsibilities

The principal investigator in charge of a clinical trial should be a clinical expert in the relevant specialty of the drug research, who must have the knowledge of the specialty and have extensive clinical experience in the specialty to deal with the medical problems that may occur in the subjects. The principal investigator is mainly responsible for the following tasks:

a) coordination, development, submission and acquisition of treatment protocols, and approval of subsequent amendments thereto;

b) Prepare the investigator's manual, develop standard operating procedures, and design case report forms;

c) ensure the use of the correct version scheme;

d) be responsible for the overall work of this research and supervise the progress of the research;

e) review and reporting of serious adverse events;

f) Review data of all departments, test data processing and statistics;

g) Assist in the writing of test reports.

14. Data Management and Security Controls

This clinical trial uses an electronic data capture system (EDC) to fill in an electronic case report form (eCRF).

14.1 Data collection

Researchers can only log on to the EDC system after they have been trained. The principal investigator (PI) or data entry staff (CRC) entered the data into the EDC system according to the eCRF filling guidelines. PI or CRC are allowed to revise the data in question, but the revision date, the signature of the reviser, and necessary explanations must be recorded truthfully, but the original records cannot be covered up.

After the database is locked, the investigator will receive a CD-ROM or file copy of the subject data. The original eCRF is preserved by Wuhan University Stomatological Hospital and is not allowed to be provided in any form except for representatives authorized by the state.

14.2 Data curation

The completed medical records and related research documents will be submitted to the research center for centralized review and data collation. The time and content of data submission are as follows:

When each subject participates in clinical trial registration:

a) Registration form

b) Signed informed consent

c) Eligibility Checklist

d) Source documentation related to eligibility and randomization

Within two weeks after registration is complete:

a) Baseline Study Case Report Form

b) Relevant source files

Within two weeks after preoperative adjuvant chemotherapy:

a) Study Case Report Form

b) Relevant source files

Investigators will be monitored, audited, and inspected by clinical trial-related bodies, including ethics committees, government regulators, and university quality management teams. Researchers should ensure that they have equipment capabilities for clinical trial research (eg pharmacy, laboratory, etc.).

14.3 Discussion Meetings

The study session will officially start once a subject has been enrolled. Study team members

participating in the trial included: study coordinators, data managers, study nurses, investigators, collaborators, and statisticians.

Key topics discussed at the meeting included: relative to expected enrollment rates, subject characteristics, subject compliance, data validity and completeness, toxicities, procedures for collecting blood samples and transferring to the laboratory and further research.

14.4 Quality Control

In order to ensure the quality of clinical trials, before the start of clinical trials, the Clinical Research Center of Wuhan University Stomatological Hospital and PI jointly discuss and formulate clinical research plans. Provide protocol training to all relevant investigators participating in clinical trials.

The PI requests Hengrui Medicine to arrange a clinical monitor. Clinical supervisors must follow the Good Clinical Practice (GCP) and standard operating procedures, conduct clinical supervision of research units on a regular basis or according to the actual situation, supervise the progress and progress of clinical trials, and check and confirm the records and reports of all data , The eCRF is filled in correctly and completely to ensure that the clinical trial is carried out in accordance with the clinical trial protocol, and the investigator should actively cooperate with the clinical supervisor. The clinical monitor or data manager will challenge the data in question to the PI or CRC in the form of an electronic challenge form, and the PI or CRC must respond to the challenge and make corrections or interpretations of the data in question, if necessary Questions are issued several times until the problem data is resolved. Medical leaders and data managers regularly perform consistent comparisons of SAEs.

At the end of the trial project, data managers and medical staff will conduct final quality control of all data in the database, summarize all protocol deviations and protocol violation events during the trial, and hold a data verification meeting. After the data in the database meets the quality requirements, the database will be locked and the test will be unblinded, and the data managers will export the data for data analysis by the statistical department.

15. Statistical analysis plan

15.1 Study design

The study was an exploratory study with two parallel groups with 1:1 randomization. The MPR rate of the camrelizumab group is expected to be 10%. We estimated a sample size of 27

cases would ensure that the precision of 95% confidence interval for the observed MPR is between 2.5%-24.9%. Considering 20% dropout, the required sample size is 34 cases. It is estimated that the MPR rate of the camrelizumab combined with TPF chemotherapy group is 70%. The precision of 95% confidence interval of the observed MPR is guaranteed to between 52.5%-84.0% if 27 cases enrolled in the group. Considering 20% dropout, the required sample size is 34 cases. In summary, the required total sample size is 68 cases.

15.2 Data Analysis Plan

15.2.1 Analysis of the basic characteristics of patients with locally advanced oral squamous cell carcinoma

The main statistics were the mean, standard deviation, median, maximum value, minimum value, gender, smoking, drinking, betel nut chewing and other data of the subjects' age, height, weight and other data, and the confidence interval.

15.2.2 Biosafety analysis of camrelizumab for preoperative treatment of advanced oral squamous cell carcinoma

Biosafety mainly analyzes the following indicators:

- Drug-related adverse events occurring within 90 days or 30 days (or longer) after the last dose of camrelizumab;

- Drug-related serious adverse events that occurred within 90 days or 30 days (or longer) after the last dose of camrelizumab;

- The most serious side effects in the laboratory results.

15.2.3 The efficacy of camrelizumab for preoperative treatment of advanced oral squamous cell carcinoma:

Pearson correlation coefficient was used to analyze the objective response rate (imaging, pathological response rate), recurrence rate, etc. and 95% confidence interval; progression-free survival rate, one-year survival rate, overall survival rate, median survival time and its 96% Confidence intervals were analyzed by Kaplan-Meier. Survival data were analyzed using Kaplan-Meier survival curves, Log-rank test and Cox proportional hazards model. Results of Cox proportional hazards model were reported as hazard ratios (HRs) with 95% confidence intervals (CIs). Descriptive analysis was used for other secondary indicators.

15.2.4 Exploratory Immune-Efficacy Correlation Analysis

For normally distributed data, Student's t-test and paired t-test were used for unpaired and paired data analysis, respectively. For normally distributed data, unpaired and paired analyses were performed using the nonparametric Mann-Whitney or Wilcoxon signed rank test. The correlation between continuous variables was analyzed by the Pearson correlation coefficient. Fisher's exact test was used to test the correlation between two categorical variables.

16. Prospective research plan

March 2021 to October 2022

Complete subject recruitment, formulate and complete all treatment plans, record subject information, adverse events, collect patient tissue, blood, and saliva samples, and follow up during the same period.

October 2022 to June 2023

Complete the follow-up of all subjects, record adverse events, disease outcomes, etc., collect blood and saliva samples from patients during the follow-up period, and complete laboratory research.

June 2023 to October 2023

Complete data sorting and analysis, and complete thesis writing and submission.

17. Informed consent

Clinical trial name: Neoadjuvant PD-1 Blockade, Camrelizumab, in Resectable Oral Squamous Cell Carcinoma

Research Institutions: Department of Oral and Maxillofacial Surgery, School and Hospital of Stomatology, Wuhan University

Dear patients:

We invite you to participate in a clinical trial here. This informed consent form provides you with information to help you decide whether to participate in this clinical trial. Please read the following carefully for a while. If there are any questions or terms that are unclear, you can discuss it with your doctor. This study has been reviewed by the Ethics Committee of School and Hospital of Stomatology, Wuhan University.

Background

Oral squamous cell carcinoma is the most common tumor in the world. After surgery combined with radiotherapy and chemotherapy, patients with local advanced oral squamous cell carcinoma (OSCC) still have high recurrence and metastasis rates. At present, neoadjuvant immunotherapy researchs have achieved good results in other cancers, and the experimental studies of PD-1 mAb have also shown that it can significantly improve the survival rate of patients with advanced inoperable OSCC. Camrelizumab is a PD-1 inhibitor approved in China and has achieved good results in classic Hodgkin's lymphoma, esophageal cancer, hepatocellular carcinoma, non-small cell lung cancer, nasopharyngeal carcinoma and other diseases. As a new adjuvant, Camrelizumab is

worthy of our in-depth study and evaluation of its role in resectable OSCC.

Objects

To clarify the safety and efficacy of camrelizumab combined with TPF chemotherapy as a neoadjuvant therapy for patients with local advanced OSCC, and to study the key molecular events and regulatory mechanisms in which patients can benefit from the above neoadjuvant therapy. To screen the liquid biopsy markers that can be used to accurately predict treatment effectiveness.

Research process

A total of 68 subjects will be enrolled in this trial were divided into two groups by randomization. One group received “camrelizumab + TPF chemotherapy, surgical resection and radiotherapy” sequence treatment; one group received “TPF chemotherapy, surgical resection and radiotherapy” sequence treatment. Follow-up will be performed every three months. If you have other special circumstances, you can return to the doctor at any time.

Relevant examinations involved during the entire treatment period include: blood, saliva and tissue biopsy samples, imaging studies during treatment; blood and saliva collection every three months and semi-annual imaging studies during follow-up

Risks and adverse reactions

Adverse reactions caused by camrelizumab: Common adverse reactions include reactive capillary hemangioma, anemia, fever, fatigue, hypothyroidism, proteinuria and cough. There may be some risks and adverse reactions that are currently unpredictable.

Adverse reactions caused by TPF chemotherapy: TPF chemotherapy may have myelosuppression, gastrointestinal reactions, liver and kidney dysfunction, cardiotoxicity, pulmonary fibrosis, and life-threatening if severe organ failure.

Adverse reactions caused by surgery: Due to the patient's condition (critical, complicated, poor systemic conditions), individual differences, sudden complications may occur during and after surgery, and multiple organ failure (such as heart failure, respiratory failure, liver failure, renal failure, DIC Etc.) or unpredictable changes in the condition can be life-threatening.

Adverse reactions caused by radiotherapy: There will be loss of appetite, fatigue, local edema, dry mouth, odorlessness, restricted mouth or neck, radiation or skin or mucous membrane reaction, skin pigmentation. Due to the difference in tumor sensitivity to radiation, there may be exist insensitive for radiotherapy, tumor residual, and tumor recurrence.

Benefits of participating

If you agree to participate in this study, you will likely receive direct medical benefits, but may not benefit. We hope that the information from this study will be instructive in the future for patients with the same condition.

In this study, you will receive a drug subsidy of up to 8,000 yuan and a transportation subsidy of 1,500 yuan. During this clinical study, if you are physically impaired or have a serious adverse event, you can get free treatment or get compensation according to the law.

You may choose not to participate in this study, or have the right to withdraw at any stage of the trial without any reason, and your medical treatment and benefits will not be affected as a result. In addition to participating in this study, you have the following options: Surgical treatment, radiation (chemotherapy) treatment sequence therapy.

Informed consent signature

If you fully understand the content of this clinical trial and agree to participate in the study, you will sign this informed consent, in duplicate, by the investigator and the patient or theclient.

1. I confirm that I have read and understood the informed consent form of this study. The

problems that may arise and the relative solutions have been explained to me, and I have the opportunity to ask my own questions. I have explicitly participated in the study as a voluntary act, and refusing to participate in the study will not harm any benefit I deserve.

2. I have learned and agreed that the person in charge of this work at the School and Hospital of Stomatology, Wuhan University and the Medical Ethics Committee of the Wuhan University Stomatological Hospital have the right to review the research records and case data. I agree to provide the samples needed for clinical research for clinical trial studies. I agree that the above-mentioned personnel can directly obtain my research records.

Full name of patient Date

Full name of legal representative Date

Full name of researcher Date

References

1. Uppaluri R, Zolkind P, Lin T, Nussenbaum B, Jackson RS, et al. Neoadjuvant pembrolizumab in surgically resectable, locally advanced HPV negative head and neck squamous cell carcinoma (HNSCC). J Clin Oncol. 2017; 35(15_suppl):6012-6012.

2. Wise-Draper TM, Old MO, Worden FP, O'Brien PE, Cohen EEW, et al. Phase II multi-site investigation of neoadjuvant pembrolizumab and adjuvant concurrent radiation and pembrolizumab with or without cisplatin in resected head and neck squamous cell carcinoma. J Clin Oncol. 2018; 36(15_suppl):6017-6017.

3. Zhong LP, Zhang CP, Ren GX, Guo W, William WN, Jr., et al. Randomized phase III trial of induction chemotherapy with docetaxel, cisplatin, and fluorouracil followed by surgery versus up-front surgery in locally advanced resectable oral squamous cell carcinoma. J Clin Oncol. 2013; 31(6):744-751.
